# Cerium-photocatalyzed aerobic oxidation of benzylic alcohols to aldehydes and ketones

**DOI:** 10.3762/bjoc.17.121

**Published:** 2021-07-23

**Authors:** Girish Suresh Yedase, Sumit Kumar, Jessica Stahl, Burkhard König, Veera Reddy Yatham

**Affiliations:** 1 School of Chemistry, Indian Institute of Science Education and Research, Thiruvananthapuram (IISER-TVM) 695551, India; 2 Institut für Organische Chemie, Fakultät für Chemie und Pharmazie, Universität Regensburg, Universitätstraße 31, D-93053 Regensburg, Germany

**Keywords:** alcohol, aldehydes, cerium, oxidation, ketones, visible light

## Abstract

We have developed a cerium-photocatalyzed aerobic oxidation of primary and secondary benzylic alcohols to aldehydes and ketones using inexpensive CeCl_3_·7H_2_O as photocatalyst and air oxygen as the terminal oxidant.

## Introduction

The selective oxidation of alcohols to carbonyl compounds [1–2] is an important process for producing a wide range of value-added fine chemicals [3–6]. In the traditional oxidation process stoichiometric amounts of oxidants such as Br_2_, MnO_2_, hypervalent iodine reagents, chromium-based reagents, activated dimethyl sulfoxide, KMnO_4_, OsO_4_, or metal-based catalysts and peroxide were used [7–17]. Most of these protocols produce harmful waste and some of the oxidizing reagents are considered toxic [7–17]. In order to overcome the limitations, various homogeneous and heterogeneous catalytic oxidation systems have been reported. Aerobic oxidation is particularly attractive as it allows the transformations under mild reaction conditions with molecular oxygen acting as the terminal oxidant [13–33]. Most aerobic oxidation reactions utilize either metal complexes and nanoparticles or persistent radical reagents as catalysts [21].

**Scheme 1:**
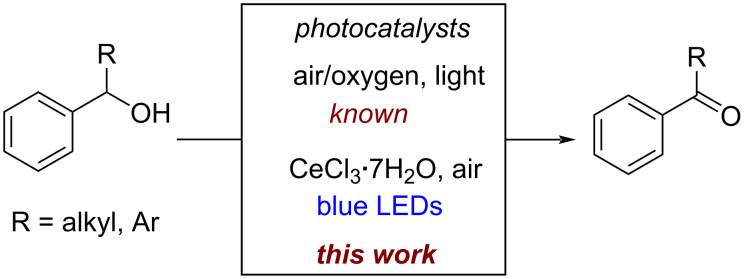
Photocatalyzed aerobic oxidation of aromatic alcohols.

In the past ten years, visible light-induced photocatalysis has emerged as an alternative to the classical conventional synthetic methods to construct carbon–carbon and carbon–heteroatom bonds [34–37]. As a mild, efficient, and environmentally friendly approach it has the potential to unlock unique reactions that are previously inaccessible under thermal conditions. Significant advances were made for the oxidation of benzylic alcohols by using metal-based photocatalysts [38–46] and metal-free photocatalysis [47–53] in combination with various oxidants, such as TBHP and DDQ [54–55]. However, the reported methods require either specific nanoparticle catalysts [39–42] or the catalytic method is limited to electron-rich or electron-neutral benzylic alcohols [56]. An operationally simple method avoiding waste and potentially toxic transition-metal catalysts that is able to convert any benzylic alcohol selectively to the aldehyde or ketone is still desirable. Recently, cerium photocatalysis was introduced as a robust alternative to generate oxygen or carbon-centered radicals under mild reaction conditions [57–64]. CeCl_3_ reacts via ligand-to-metal charge transfer generating oxygen-centered radicals, that lead to carbon-centered radicals through intra/intermolecular hydrogen atom transfer (HAT) processes, radical decarboxylative or radical deformylation [57–59]. In continuation of our research interest on visible-light-driven cerium photocatalysis [59,65], we herein report a mild aerobic photocatalytic oxidation of benzylic alcohols to aldehydes and ketones using 10 mol % CeCl_3_·7H_2_O (Scheme 1).

## Results and Discussion

A variety of reaction parameters was tested during the optimization of the reaction with 4-iodobenzyl alcohol (**1a**) as the model substrate and air as the oxidant (Table 1). The best results were found using 10 mol % CeCl_3_·7H_2_O as a photocatalyst and 10 mol % of NaHCO_3_ as a base in CH_3_CN under blue LED irradiation at 50 °C for 35 h giving compound **2a** in 65% isolated yield (Table 1, entry 1). The product formation was reduced upon employing other cerium salts (Table 1, entries 2 and 3). Also, replacing NaHCO_3_ by other bases such as K_2_CO_3_ and Na_2_CO_3_ resulted in lower yields (30–40%) of product **2a** (Table 1, entries 4 and 5). In the absence of a base the reaction afforded product **2a** in 40% yield (Table 1, entry 6). The reaction worked with similar efficiency in CHCl_3_ and DMF (Table 1, entries 7 and 8), while other solvents such as toluene and EtOAc gave **2a** in moderate yields (Table 1, entries 9 and 10). THF was found to be less effective in this oxidation reaction (Table 1, entry 11). Performing the reaction at 35 °C gave **2a** in a moderate yield of 35% (Table 1, entry 12). Employing an external oxidant such as (NH_4_)_2_S_2_O_8_ instead of air diminished the yield (Table 1, entry 13). The substitution of air with a balloon of oxygen afforded **2a** in 25% yield (Table 1, entry 14), while employing an argon atmosphere led to only trace amounts of the product (Table 1, entry 15). Additionally, control experiments indicated that catalytic amounts of the cerium salt, air atmosphere and light irradiation were necessary for the reaction to occur (Table 1, entries 16 and 17).

**Table 1:** Optimization of the reaction conditions.^a^

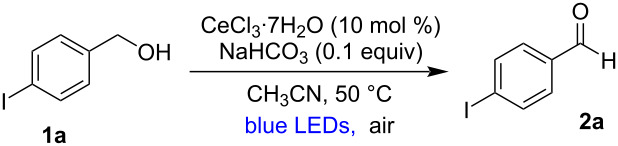		

entry	deviation from standard conditions	**2a** (%)^b^

1	none	70 (65)^c^
2	CeCl_3_ instead of CeCl_3_·7H_2_O	60
3	(*n*-Bu_4_N)_2_Ce^IV^Cl_6_ instead of CeCl_3_·7H_2_O	42
4	K_2_CO_3_ instead of NaHCO_3_	40
5	Na_2_CO_3_ instead of NaHCO_3_	30
6	without base	40
7	CHCl_3_ instead of CH_3_CN	56
8	DMF instead of CH_3_CN	60
9	toluene instead of CH_3_CN	30
10	EtOAc instead of CH_3_CN	21
11	THF instead of CH_3_CN	5
12	at 35 °C instead of 50 °C	35
13	with 2.0 equiv of (NH_4_)_2_S_2_O_8_ instead of air	28
14	with O_2_ balloon instead of air	25
15	under argon instead of air	trace
16	without CeCl_3_·7H_2_O	0
17	without light	trace

^a^Standard conditions: **1a** (0.2 mmol), CeCl_3_**^.^**7H_2_O (10 mol %), NaHCO_3_ (10 mol %), CH_3_CN (0.1 M) at 50 °C, 455 nm blue LED for 35 h. ^b^NMR yields using trimethoxybenzene as internal standard. ^c^Isolated yield.

**Scheme 2:**
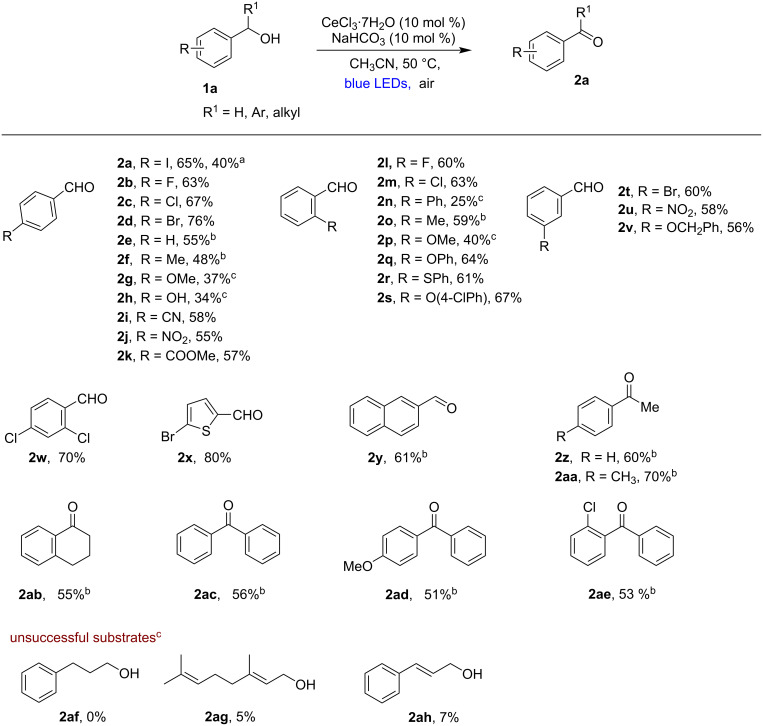
Substrate scope. Reaction conditions as given in Table 1 (entry 1). Yields are isolated yields, average of at least two independent runs. Notes: ^a^the reaction was carried using 4.3 mmol of **1a** and reaction time was 72 h; ^b^42 h reaction time; ^c^48 h reaction time.

With the optimized reaction parameters in our hands, we next explored the substrate scope of the reaction. As shown in Scheme 2, a broad range of primary and secondary benzylic alcohols was converted into the corresponding aldehydes and ketones. Various electron-withdrawing *para*-halo-substituted benzylic alcohols **1a–d** were tested under the optimized reaction conditions and gave the corresponding halo-substituted benzaldehydes **2a**–**d** in good yields. The oxidation of simple benzyl alcohol (**1e**) under our reaction conditions gave benzaldehyde (**2e**) in 55% yield. A variety of electron-donating *para*-substituted benzyl alcohols (**1f–h**) gave lower isolated yields of the corresponding benzaldehydes **2f–h**. Our methodology tolerates a variety of functional groups containing benzylic alcohols such as -OH (**1h**), -CN (**1i**), -NO_2_ (**1j**), methyl ester (**1k**), and benzyloxy (**1v**) to produce the corresponding aldehydes (**2h–k** and **2v**) in moderate yields. Next, electronically different *ortho*-substituted benzylic alcohols were tested and 2-fluoro (**1l**) and 2-chloro (**1m**) benzyl alcohols gave the aldehydes **2l** and **2m** in good yields. The *o*-phenyl-substituted benzylic alcohol (**1n**) afforded biphenyl-2-carbaldehyde (**2n**) in only low yield (25%) probably due to steric reasons. The *o*-methyl (**1o**) and *o*-methoxy (**1p**) benzylic alcohols yielded the corresponding benzaldehydes **2o** and **2p** in moderate yields and to our surprise we did not observe any oxidation of the methyl or methoxy groups via hydrogen atom transfer processes [57]. Interestingly, we found that a variety of *ortho*-phenoxy-substituted benzylic alcohols (**1q**, **1s**) were oxidized under our reaction conditions giving the corresponding aldehydes (**2q**, **2s**) in good yields. Also, the *meta*-substituted benzylic alcohols **1t–v** reacted to the corresponding benzaldehydes in good yields in our reaction conditions. *Ortho/para*-disubstituted benzylic alcohol **1w** gave 2,4-dichlorobenzaldehyde (**2w**) in 70% yield. The sulfur-containing compounds 4-(phenylthio)benzyl alcohol (**1r**) and the heterocyclic compound thiophene-2-ylmethanol (**1x**) gave the corresponding aldehydes **2r** and **2x** in 61 and 80% yield, respectively. Finally, 2-naphthylmethanol (**1y**) was subjected to the reaction conditions and gave 2-naphthaldehyde (**2y**) in good yield (61%).

**Scheme 3:**
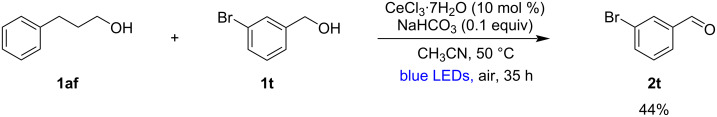
Selective oxidation of 3-bromobenzyl alcohol in the presence of 3-phenylpropanol. Compound **1af** was recovered unchanged from the reaction mixture.

Next, the scope of secondary benzylic alcohols was tested in our reaction conditions. Substituted 1-phenylethanols such as **1z**, **1aa**, tetralol (**1ab**), diphenyl methanol (**1ac**) and derivatives thereof with substituents of different electronic nature such as **1ad** and **1ae** gave the ketones **2z**, **2aa**, **2ab**, **2ac**, **2ad**, and **2ae** in good yields. However, the primary aliphatic alcohol 3-phenylpropanol (**1af**) did not provide the desired aldehyde at all, and allylic alcohols such as geraniol (**1ag**) and cinnamyl alcohol (**1ah**) afforded the aldehydes **2ag** and **2ah** in very low yields (5 and 7%, respectively). In addition, when the mixture of 3-phenylproponol (**1ah**) and 3-bromobenzylic alcohol (**1t**) was subjected to the standard reaction conditions, we observed the selective oxidation of the benzylic alcohol giving the expected product in 44% yield (Scheme 3).

**Figure 1:**
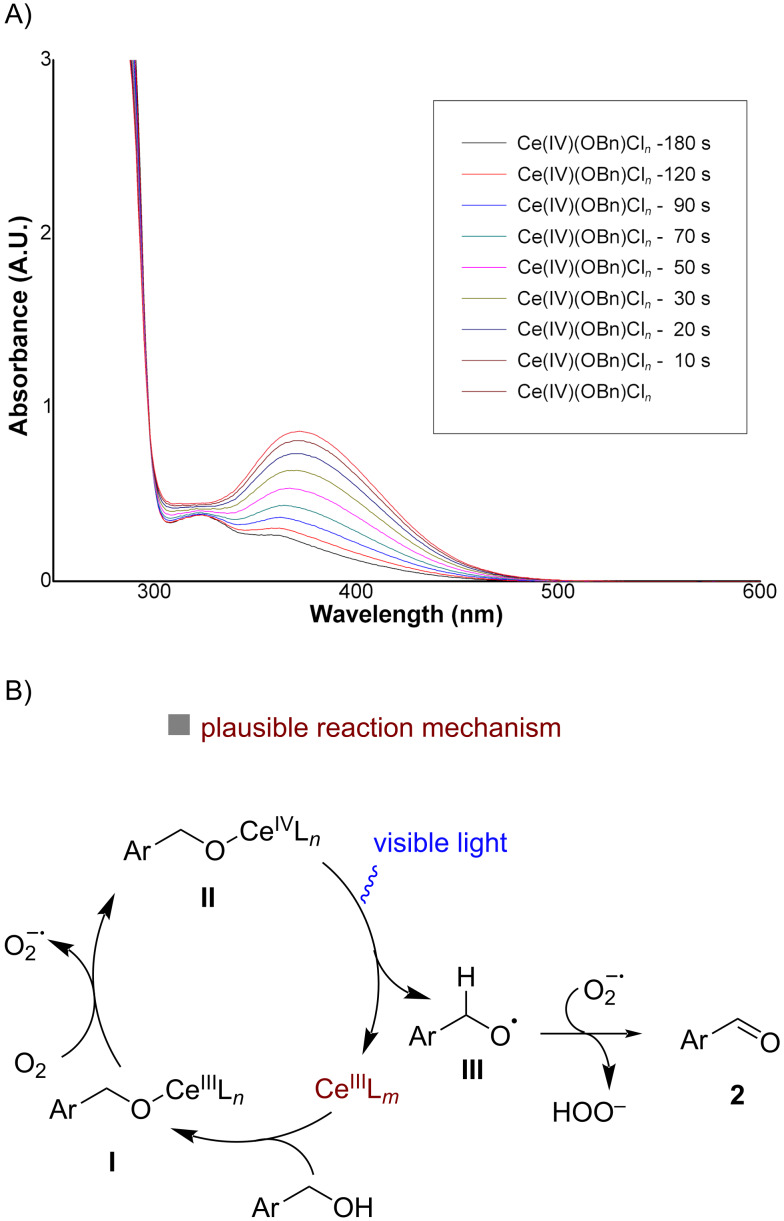
Mechanistic studies. (A): UV–vis spectra of the Ce^IV^(OBn)Cl*_n_* complex in CH_3_CN under blue light irradiation (0–180 s); (B): plausible reaction mechanism.

The efficiency of this cerium-photocatalyzed aerobic oxidation of alcohols prompted us to conduct some preliminary mechanistic studies (Figure 1). As anticipated, the ON/OFF irradiation experiments confirmed that our reaction required a continuous blue light irradiation (see Supporting Information File 1). The inhibition of the catalytic cycle upon the addition of TEMPO revealed that the reaction proceeds through radical intermediates. Next, we carried out UV–vis monitoring experiments in order to verify whether the interaction with the substituted benzyl alcohols and Ce^IV^ could lead to a ligand-to-metal charge transfer (LMCT) process, which reduces the Ce^IV^ species to Ce^III^, similarly as reported by Zuo and co-workers [57]. We chose (*n*-Bu_4_N)_2_Ce^IV^Cl_6_ as the Ce^IV^ source to ensure a sufficient solubility in organic solvents and to facilitate the detection of the species. The Ce^IV^(OBn)Cl*_n_* complex was prepared by mixing the (*n*-Bu_4_N)_2_Ce^IV^Cl_6_ complex with BnOH under basic conditions. The UV–vis spectra of the Ce^IV^(OBn)Cl*_n_* complex displayed a band resembling the LMCT band of known cerium–alkoxide complexes, showing considerable overlap with the blue LED region, thus suggesting that the Ce^IV^(OBn)Cl*_n_* species could be photoexcited (Figure 1A). We then analyzed UV–vis spectra of the Ce^IV^(OBn)Cl*_n_* complex recorded after irradiation with blue light at different time intervals. As shown in Figure 1A, the absorption spectrum of the Ce^IV^(OBn)Cl*_n_* complex gradually shifted from λ_max_ = 375 nm to λ_max_ = 325 nm upon irradiation, which indicates a photoinduced Ce^IV^–OBn homolytic cleavage to generate a Ce^III^ complex and a benzyloxy radical. Although the exact catalytic cycle of our reaction remains to be elucidated, we propose a plausible reaction mechanism based on our observations and known literature precedents (Figure 1B) [57,59,66–69]. Under aerobic conditions the catalytic Ce^III^(OBn)L*_n_* species **I** (in situ derived by the reaction of CeCl_3_ (Ce^III^L*_n_*) with the substrate benzyl alcohol, BnOH) could be oxidized to L*_n_*Ce^IV^–OBn complex **II** [67–69]. During this process O_2_ is converted into a superoxide radical anion O_2_^•−^. Photolysis of the Ce^IV^–OBn complex (**II**), leads to the formation of the corresponding benzyloxy radical (**III**) and regenerates the Ce^III^ species. A further abstraction of a benzylic hydrogen atom by the peroxide radical then generates the final product **2** [48]. However, at this moment we cannot exclude the involvement of possible intermolecular HAT or 1,2-HAT from the intermediate **III** to generate the product **2**.

## Conclusion

In summary, we have developed a catalytic aerobic oxidation of benzylic alcohols to the corresponding aldehydes without further oxidation and formation of benzoic acids. A variety of primary and secondary benzylic alcohols were converted into the corresponding aldehydes and ketones in good to moderate yields using commercially available and inexpensive CeCl_3_**·**7H_2_O as a photocatalyst and air as an oxidant.

## Supporting Information

File 1 Full experimental details, compound characterization, and copies of NMR spectra.
