# Supplementary material for: Cerium-photocatalyzed aerobic oxidation of benzylic alcohols to aldehydes and ketones
Source: Beilstein J Org Chem. 2021 Jul 23;17:1727–32. doi: 10.3762/bjoc.17.121 (PMC8313980; doi:10.3762/bjoc.17.121)
Supplement: File 1 — Full experimental details, compound characterization, and copies of NMR spectra. [file Beilstein_J_Org_Chem-17-1727-s001.pdf]

BEILSTEIN JOURNAL OF ORGANIC CHEMISTRY

Supporting Information

for

Cerium-photocatalyzed aerobic oxidation of benzylic alcohols to aldehydes and ketones

Girish Suresh Yedase, Sumit Kumar, Jessica Stahl, Burkhard König and Veera Reddy Yatham

Beilstein J. Org. Chem. **2021,** *17,* 1727–1732. doi:10.3762/bjoc.17.121

Full experimental details, compound characterization, and copies of NMR spectra

License and Terms: This is a supporting information file under the terms of the Creative Commons Attribution License (https://creativecommons.org/licenses/by/4.0). Please note that the reuse, redistribution and reproduction in particular requires that the author(s) and source are credited and that individual graphics may be subject to special legal provisions.
The license is subject to the *Beilstein Journal of Organic Chemistry* terms and conditions: (https://www.beilstein-journals.org/bjoc/terms)

Table of contents

General considerations....................S2

Optimization details....................S3

General procedure for the oxidation of benzylic alcohols....................S5

Characterization of products....................S6

Mechanistic studies....................S18

References....................S22

NMR spectra....................S23

General considerations

Unless otherwise stated, all commercial reagents and solvents were purchased from commercial sources (Sigma-Aldrich, Merck, TCI, ACROS Organics, Spectrochem) and used as received without additional purification. All reactions were conducted in 10 mL crimp glass vials. Analytical thin layer chromatography (TLC) was performed on precoated silica gel 60 F254 plates. Visualization via TLC was achieved by the use of UV light (254 nm). Column chromatography was undertaken on silica gel (100–200 mesh) using a proper eluent system. NMR spectra were recorded in chloroform-*d* at 500 MHz for ^{1}H NMR spectra and 125 MHz for ^{13}C NMR spectra. ^{19}F NMR NMR spectra were recorded in chloroform-*d* at 377 MHz. Chemical shifts are quoted in parts per million referenced to the appropriate solvent peak. The following abbreviations were used to describe peak splitting patterns when appropriate: s, singlet; d, doublet; t, triplet; q, quartet; sept, septet; dd, doublet of doublets; td, triplet of doublets; m, multiplet. Coupling constants, *J*, are reported in hertz. For ^{13}C NMR, chemical shifts are reported in parts per million referenced to the center of the triplet at 77.0 ppm of chloroform-*d*. Benzyl alcohols **1i**, **1n**, **1x,** and **1y** were synthesized according to the literature.[1a] Benzyl alcohols **1q, 1r,** and **1s** were synthesized according to the literature.[1b] Diphenyl methanol derived compounds **1ad** and **1ae** were synthesized according to the literature.[1c]

Photochemical reactions were irradiated with 455 nm LEDs (OSRAM Oslon® SSL 80 royal-blue LEDs (λ_{max} = 455 nm (± 15 nm), 3.5 V, 700 mA), which were installed on a passive cooling system at the bottom (7 mm from the bottom plane of the vials) of a custom-made 6-vials reactor (aluminium), which was equipped with a liquid cooling system (see Figure S1).

Optimization details

General procedure for screening reactions: A 10 mL glass vial was charged with benzylic alcohol (0.2 mmol), Ce photocatalyst (10 mol %), $NaHCO_3$ (10 mol %) and a PTFE-coated stirring bar, and the glass vial was sealed with a PTFE septum. Solvent (2 mL) was added and the reaction was opened to air via a needle. The reactions were placed in a pre-programed temperature (50 °C) controlled blue LED reactor (as shown in Figure S1) and the reaction mixture was irradiated with a 455 nm blue LED. After 35 hours, a sample of the solution was analyzed by ^{1}H NMR using trimethoxybenzene as the internal standard to determine the yield.

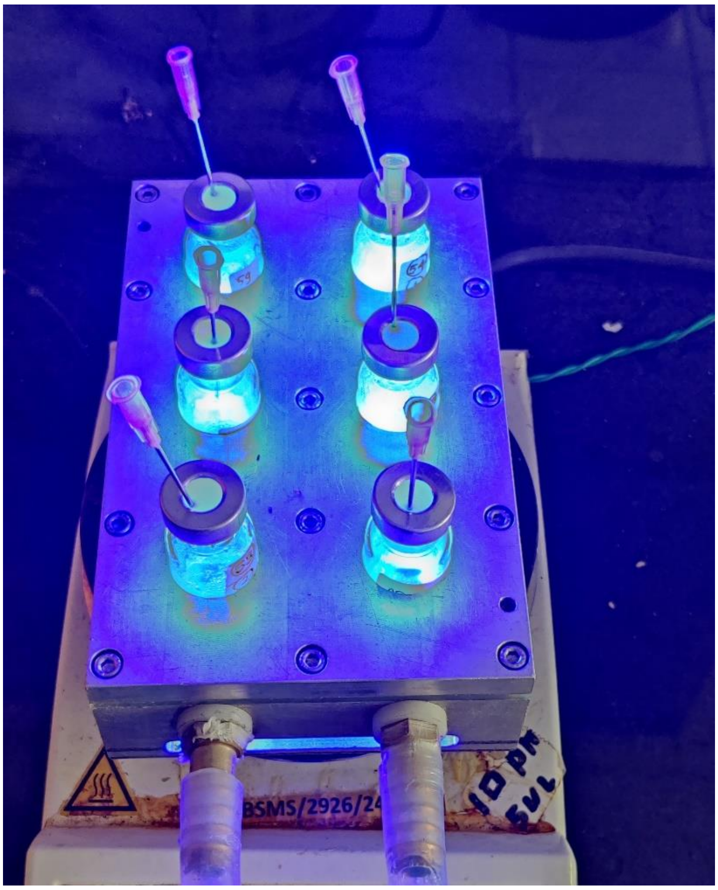

Figure S1: Blue LED reactor with magnetic stirring plate.

Table S1: Optimization of the reaction conditions (**1a** (0.2 mmol), $CeCl_3 \cdot 7H_2O$ (10 mol %), solvent (2 mL) at 50 °C, 455 nm blue LED for 35 h).

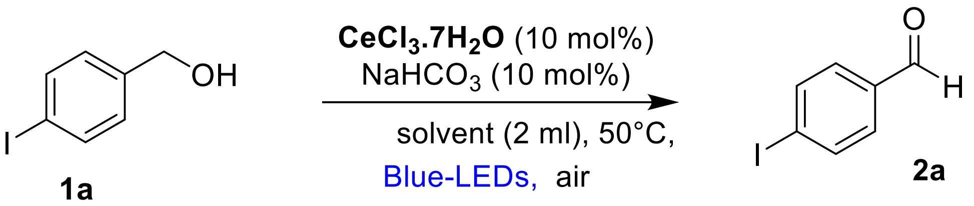

Entry	Deviation from standard conditions	**2a** (%)[a]
1	***none***	***70***(*65*)[b]
2	**$CeCl_3$** instead of **$CeCl_3 \cdot 7H_2O$**	60
3	**$(^nBu_4N)_2Ce^{IV}Cl_6$** instead of **$CeCl_3 \cdot 7H_2O$**	42
4	**K_2CO_3** instead of **$NaHCO_3$**	40
5	**Na_2CO_3** instead of **$NaHCO_3$**	30
6	**Toulene** instead of **CH_3CN**	30
7	**$CHCl_3$** instead of **CH_3CN**	56
8	**EtOAc** instead of **CH_3CN**	21
9	**DMF** instead of **CH_3CN**	60
10	**THF** instead of **CH_3CN**	5
11	**(1,4) dioxane** instead of **CH_3CN**	14
12	**DMA** instead of **CH_3CN**	10
13	**DMSO** instead of **CH_3CN**	0
14	**DCM** instead of **CH_3CN**	36
15	At 35 °C instead of 50 °C	35
16	with 2.0 eq. of **$(NH_4)_2S_2O_8$** instead of air	28
17	with **O_2** balloon instead of air	25
18	with **Ar** balloon instead of air	trace
19	without **$CeCl_3 \cdot 7H_2O$**	0
20	without base	40
21	without light	trace
22	Blue LED (400 nm)	65
23	Green LED (560 nm)	0

[a]NMR yields using trimethoxybenzene as internal standard. [b]Isolated yield.

General procedure for the oxidation of benzylic alcohols

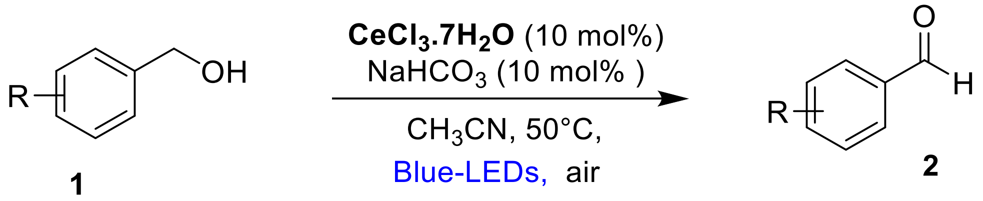

General procedure (GP1): A 10 mL glass vial equipped with a teflon-coated stirring bar was charged with benzylic alcohol **1** (0.2 mmol), $CeCl_3 \cdot 7H_2O$ (10 mol %), and $NaHCO_3$ (10 mol %). The glass vial was sealed with a PTFE septum. Then, solvent (2 mL) was added and the reaction was opened to air via a needle. The reaction was placed in a pre-programed temperature (50 °C) controlled blue LED reactor (as shown in Figure S1) and the reaction mixture was irradiated with a 455 nm blue LED. After 35–48 hours, the reaction mixture was concentrated under reduced pressure. Product **2** was purified by flash chromatography on silica using hexane and AcOEt.

Note: At same time we run two independent reactions for each substrate and the obtained yield is the combined average yield of these two independent runs.

Characterization of products

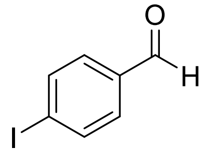

4-Iodobenzaldehyde (2a): Following the general procedure **GP1**, two parallel reactions of **1a** (0.2 mmol each one, 46.8 mg) were conducted. After 35 h the reaction mixture was concentrated under reduced pressure. The product **2a** was purified by flash chromatography on silica using hexane/AcOEt 98:2 afforded as light yellow oil (60 mg, 65% yield). ^{1}H NMR (500 MHz, $CDCl_3$) δ 9.95 (s, 1H), 7.91 (d, J = 8 Hz, 2H), 7.59 (d, J = 8 Hz, 2H). ^{13}C NMR (125 MHz, $CDCl_3$) δ 191.55, 138.56, 135.71, 130.94, 102.96. The analytical data are consistent with published ones. [2]

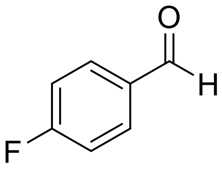

4-Fluorobenzaldehyde (2b): Following the general procedure **GP1**, two parallel reactions of **1b** (0.2 mmol each one, 25.2 mg) were conducted. After 35 h the reaction mixture was concentrated under reduced pressure. Product **2b** was purified by flash chromatography on silica using hexane/AcOEt 98:2 affording a light yellow oil (31.2 mg, 63% yield). ^{1}H NMR (500 MHz, $CDCl_3$) δ 9.97 (s, 1H), 7.93-7.90 (m, 2H), 7.23-7.20 (m 2H). ^{13}C NMR (125 MHz, $CDCl_3$) δ 190.65, 167.7 (d, JCF = 255 Hz), 133.1 (d, JCF = 2.56 Hz), 132.4 (d, JCF = 9.6 Hz), 116.6 (d, JCF = 22 Hz). The analytical data are consistent with published ones. [3]

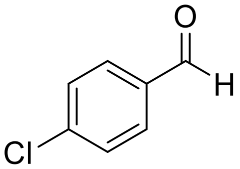

4-Chlorobenzaldehyde (2c): Following the general procedure **GP1**, compound **1c** (0.2 mmol each one, 28.4 mg) was converted in two parallel reactions. After 35 h the reaction mixture was concentrated under reduced pressure. Product **2c** was purified by flash chromatography on silica using hexane/AcOEt 98:2 affording a light yellow semi solid (37.5 mg, 67% yield). ^{1}H NMR (500 MHz, $CDCl_3$) δ 9.98 (s, 1H), 7.82 (d, *J* = 8.5Hz, 2H), 7.51 (d, *J* = 8.5Hz, 2H)· ^{13}C NMR (125 MHz, $CDCl_3$) δ 190.98, 141.09, 134.85, 131.04, 129.59. The analytical data are consistent with published ones. [3]

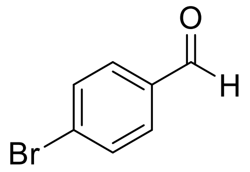

4-Bromobenzaldehyde (2d): Following the general procedure **GP1**, two parallel reactions of **1d** (0.2 mmol each one, 37.2 mg) were performed. After 35 h the reaction mixture was concentrated under reduced pressure. Product **2d** was purified by flash chromatography on silica using hexane/AcOEt 98:2 followed by hexane/AcOEt 90:10 to afford a white solid (56.2 mg, 76%). ^{1}H NMR (500 MHz, $CDCl_3$) δ 9.98 (s, 1H), 7.75 (d, *J* = 8.5Hz, 2H), 7.69 (d, *J* = 8.5Hz, 2H). ^{13}C NMR (125 MHz, $CDCl_3$) δ 191.22, 135.23, 132.60, 131.12, 129.94. The analytical data are consistent with published ones. [3]

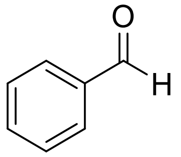

Benzaldehyde (2e): Following the general procedure **GP1**, two reactions of **1e** (0.2 mmol each one, 21.6mg) were performed. After 42 h the reaction mixture was concentrated under reduced pressure. Product **2e** was purified by flash chromatography on silica using hexane/AcOEt 98:2 to afford a pale yellow oil (23.3 mg, 55%). ^{1}H NMR (500 MHz, $CDCl_3$) δ 10.03 (s, 1H), 7.89 (d, *J* = 8 Hz, 2H), 7.63 (t, *J* = 8 Hz, 1H), 7.53 (t, *J* = 8 Hz, 2H). ^{13}C NMR (125 MHz, $CDCl_3$) δ 192.54, 136.54, 134.59, 129.83, 129.13. The analytical data are consistent with published ones. [4]

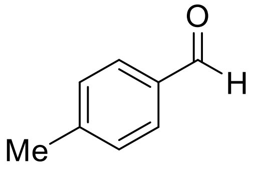

4-Methylbenzaldehyde (2f): Following the general procedure **GP1**, two reaction batches of **1f** (0.2 mmol each one, 24.4 mg) were converted. After 42 hthe reaction mixture was concentrated under reduced pressure. Product **2f** was purified by flash chromatography on silica using hexane/AcOEt 98:2 to afford a light yellowish oil (23 mg, 48% yield). ^{1}H NMR (500 MHz, $CDCl_3$) δ 9.96 (s, 1H), 7.78 (d, *J* = 8Hz, 2H), 7.33 (d, *J* = 8Hz, 2H), 2.44 (s, 3H) ^{13}C NMR (125 MHz, $CDCl_3$) δ 192.16, 145.70, 134.36, 130.01, 129.86, 22.03. The analytical data are consistent with published ones. [3]

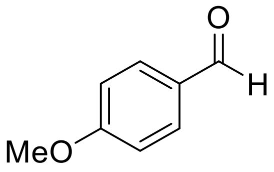

4-Methoxybenzaldehyde (2g): Following the general procedure **GP1**, two reaction batches of **1g** (0.2 mmol each one, 27.6 mg) were converted. After 48 h the reaction mixture was concentrated under reduced pressure. Product **2g** was purified by flash chromatography on silica using hexane/AcOEt 98:2 to give a light yellow oil (20.1 mg, 37% yield). ^{1}H NMR (500 MHz, $CDCl_3$) δ 9.89 (s, 1H), 7.84 (d, *J* = 8.6Hz, 2H), 7.01 (d, *J* = 8.6 Hz, 2H), δ 3.89 (s, 3H). ^{13}C NMR (125 MHz, $CDCl_3$) δ 190.96, 164.76, 132.14, 130.14, 114.47, 55.74. The analytical data are consistent with published ones.[5]

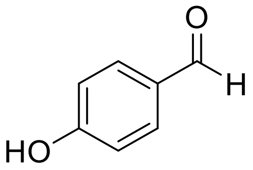

4-Hydroxybenzaldehyde (2h): Following the general procedure **GP1**, two parallel reactions of **1h** (0.2 mmol each one, 25 mg) were conducted. After 48 h the reaction mixture was concentrated under reduced pressure. Product **2h** was purified by flash chromatography on silica using hexane/AcOEt 95:5 to give a light brown solid (16.6 mg, 34% yield). ^{1}H NMR (500 MHz, $CDCl_3$) δ 9.86 (s 1H), 7.82 (d, *J* = 8.5 Hz, 2H), 6.98 (d, *J* = 8.5 Hz, 2H), 6.49 (bs, 1H). ^{13}C NMR (125 MHz, $CDCl_3$) δ 191.48, 161.85, 132.71, 129.95, 116.18. The analytical data are consistent with published ones. [6]

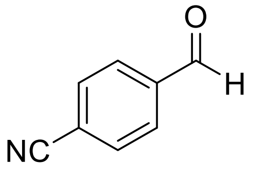

4-Formylbenzonitrile (2i): Following the general procedure **GP1**, two reaction batches of **1i** (0.2 mmol each one, 26.6 mg) were converted. After 35 h the reaction mixture was concentrated under reduced pressure. Product **2i** was purified by flash chromatography on silica using hexane/AcOEt 98:2 affording a semi solid (30.4.mg, 58%). ^{1}H NMR (500 MHz, $CDCl_3$) δ 10.09 (s, 1H), 8.0 (d, *J* = 8 Hz, 2H), 7.85 (d, *J* = 8 Hz, 2H). ^{13}C NMR (125 MHz, $CDCl_3$) δ 190.76, 138.90, 133.06, 130.80, 130.05, 117.80. The analytical data are consistent with published ones. [4]

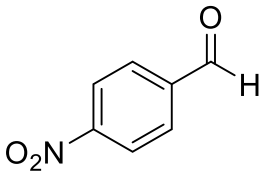

4-Nitrobenzaldehyde (2j): Following the general procedure **GP1**, two batches of **1j** (0.2 mmol each one, 30.6 mg) were reacted. After 35 h the reaction mixture was concentrated under reduced pressure. Product **2j** was purified by flash chromatography on silica using hexane/AcOEt 95:5 affording a yellow semi solid (33.2 mg, 55% yield) ^{1}H NMR (500 MHz, $CDCl_3$) δ 10.16 (s, 1H), 8.40 (d, *J* = 8.5 Hz, 2H), 8.08 (d, *J* = 8.5 Hz, 2H). ^{13}C NMR (125 MHz, $CDCl_3$) δ 190.41, 151.29, 140.20, 130.63, 124.46. The analytical data are consistent with published ones. [4]

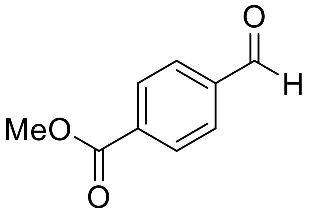

Methyl 4-formylbenzoate (2k): Following the general procedure **GP1**, two batches of **1k** (0.2 mmol each one, 33.2 mg) were reacted. After 35 h the reaction mixture was concentrated under reduced pressure. Product **2k** was purified by flash chromatography on silica using hexane/AcOEt 95:5 to give a semi solid (37.4 mg, 57% yield). ^{1}H NMR (500 MHz, $CDCl_3$) δ 10.10 (s, 1H), 8.20 (d, *J* = 8 Hz, 2H), 7.96 (d, *J* = 8 Hz, 2H), 3.96 (s, 3H). ^{13}C NMR (125 MHz, $CDCl_3$) δ 191.78, 166.21, 139.29, 135.24, 130.34, 129.66, 52.73. The analytical data are consistent with published ones. [7]

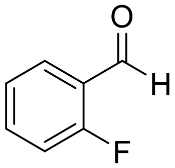

2-Fluorobenzaldehyde (2l): Following the general procedure **GP1**, two batches of **1l** (0.2 mmol each one, 25.2 mg) were reacted. After 35 h the reaction mixture was concentrated under reduced pressure. Product **2l** was purified by flash chromatography on silica using hexane/AcOEt 98:2 followed by hexane/AcOEt 90:10) affording a yellowish oil (29.7 mg, 60% yield). ^{1}H NMR (500 MHz, $CDCl_3$) δ 10.40 (s, 1H), 7.92-7.90 (m, 1H), 7.66-7.61 (m, 1H), 7.31-7.28 (m, 1H), 7.22-7.20 (m, 1H). ^{13}C NMR (125 MHz, $CDCl_3$) δ 187.40 (d, JCF = 6.6 Hz), 165.91 (d, JCF = 257 Hz), 136.53 (d, JCF = 9 Hz) 128.88 (d, JCF = 1.75 Hz), 124.81 (d,

JCF = 3.75 Hz), 124.38 (d, JCF = 8 Hz), 116.75 (d, JCF = 20 Hz). The analytical data are consistent with published ones. [8]

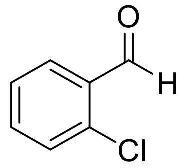

2-Chlorobenzaldehyde (2m): Following the general procedure **GP1**, two batches of compound **1m** (0.2 mmol each one, 28.4 mg) were reacted. After 35 h the reaction mixture was concentrated under reduced pressure. Product **2m** was purified by flash chromatography on silica using hexane/AcOEt 98:2 affording an oil (36.4 mg, 63% yield). ^{1}H NMR (500 MHz, $CDCl_3$) δ 10.49 (s, 1H), 7.93 (dd, *J* = 7.5Hz, 2.0 Hz, 1H), 7.57-7.53 (m, 1H), 7.49-7.46 (m, 1H), 7.40-7.38 (m, 1H). ^{13}C NMR (125 MHz, $CDCl_3$) δ 190.00, 138.11, 135.27, 132.63, 130.77, 129.54, 127.44. The analytical data are consistent with published ones. [9]

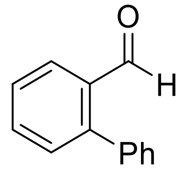

[1,1'-biphenyl]-2-carbaldehyde (2n): Following the general procedure **GP1**, two batches of compound **1n** (0.2 mmol each one, 36.8 mg) were converted and after 48 h the reaction mixture was concentrated under reduced pressure. Product **2n** was purified by flash chromatography on silica using hexane/AcOEt 95:5 yielding an oil (18.2 mg, 25% yield). ^{1}H NMR (500 MHz, $CDCl_3$) δ 9.99 (s, 1H), 8.04-8.02 (m, 1H), 7.66-7.63 (m, 1H), 7.52-7.45 (m, 5H), 7.39-7.38 (m, 2H). ^{13}C NMR (125 MHz, $CDCl_3$) δ 192.62, 146.13, 137.91, 133.88, 133.71, 130.92, 130.25, 128.58, 128.27, 127.93, 127.72. The analytical data are consistent with published ones. [10]

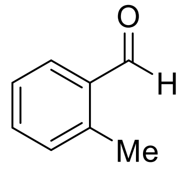

2-Methylbenzaldehyde (2o): Following the general procedure **GP1**, two batches of compound **1** (0.2 mmol each one, 24.4mg) were reacted and after 42 h the reaction mixture was concentrated under reduced pressure. Product **2** was purified by flash chromatography on silica using hexane/AcOEt 98:2 followed by hexane/AcOEt 90:10 affording a yellowish oil (28.3 mg, 59 %). ^{1}H NMR (500 MHz, $CDCl_3$) δ 10.28 (s, 1H), 7.81-7.80 (m, 1H), 7.50-7.47 (m, 1H), 7.38-7.35 (m,1H), 7.27 (m,1H), 2.68 (s,3H). ^{13}C NMR (125 MHz, $CDCl_3$) δ 192.98, 140.78,

134.32, 133.80, 132.20, 131.93, 126.48, 19.73. The analytical data are consistent with published ones. [11]

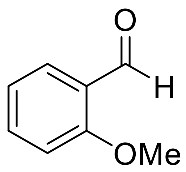

2-Methoxybenzaldehyde (2p): Following the general procedure **GP1**, two batches of compound **1p** (0.2 mmol each one, 27.6 mg) were converted and after 48 h the reaction mixture was concentrated under reduced pressure. Product **2p** was purified by flash chromatography on silica using hexane/AcOEt 98:2 followed by hexane/AcOEt 90:10 giving a semi solid (21.7 mg, 40%). ^{1}H NMR (500 MHz, $CDCl_3$) δ 10.46 (s, 1H), 7.83-7.81 (m, 1H), 7.56-7.53 (m, 1H), 7.03-6.98 (m, 2H), 3.92 (s, 3H). ^{13}C NMR (125 MHz, $CDCl_3$) δ 189.99, 161.98, 136.08, 128.71, 124.99, 120.81, 111.76, 55.76. The analytical data are consistent with published ones. [12]

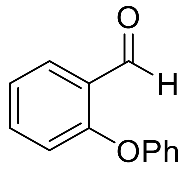

2-Phenoxybenzaldehyde (2q): Following the general procedure **GP1**, two batches of compound **1q** (0.2 mmol each one, 40 mg) were reacted and after 35 h the reaction mixture was concentrated under reduced pressure. Product **2q** was purified by flash chromatography on silica using hexane/AcOEt 95:5 affording a light yellow solid (50.6 mg, 64% yield). ^{1}H NMR (500 MHz, $CDCl_3$) δ 10.52 (s, 1H), 7.95-7.93 (m, 1H), 7.53-7.49 (m, 1H), 7.41-7.38 (m, 2H), 7.20-7.17 (m, 2H), 7.07 (d, J = 7.5 Hz, 2H), 6.90 (d, J = 8Hz, 1H). ^{13}C NMR (125 MHz, $CDCl_3$) δ 189.50, 160.13, 156.56, 135.87, 130.23, 128.59, 127.07, 124.46, 123.46, 119.53, 118.63. The analytical data are consistent with published ones. [13]

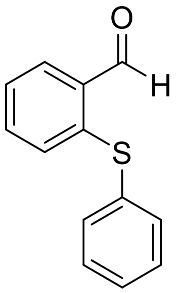

2-(Phenylthio)benzaldehyde (2r): Following the general procedure **GP1**, two batches of compound **1r** (0.2 mmol each one, 43.2 mg) were reacted and after 35 h the reaction mixture was concentrated under reduced pressure. Product **2r** was purified by flash chromatography on silica using hexane/AcOEt 95:5 followed by hexane/AcOEt 90:10 affording a light yellow semi solid (52.2 mg, 61% yield). ^{1}H NMR (500 MHz, $CDCl_3$) δ 10.39 (s, 1H), 7.89-7.87 (m, 1H), 7.44-7.33 (m, 7H), 7.10 (d, *J* = 8Hz, 1H). ^{13}C NMR (125 MHz, $CDCl_3$) δ 191.70, 141.70, 134.22, 133.93, 133.43, 133.27, 132.01, 130.56, 129.85, 128.56, 126.46. The analytical data are consistent with published ones. [14]

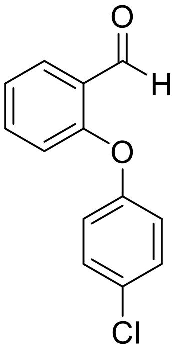

2-(4-Chlorophenoxy)benzaldehyde (2s): Following the general procedure **GP1**, two batches of compound **1s** (0.2 mmol each one, 46.8 mg) were reacted and after 35 h the reaction mixture was concentrated under reduced pressure. Product **2s** was purified by flash chromatography on silica using hexane/AcOEt 95:5 followed by hexane/AcOEt 90:10 giving a light yellow solid (62.1 mg, 67% yield). ^{1}H NMR (500 MHz, $CDCl_3$) δ 10.40 (s, 1H), δ 7.86 (dd, *J* = 7.5Hz, 2.0 Hz, 1H), 7.47-7.44 (m, 1H), 7.27 (d, *J* = 9Hz, 2H), 7.13 (t, *J* = 7.5Hz, 1H), 6.93 (d, *J* = 9Hz, 2H), 6.82 (d, *J* = 8Hz, 1H). ^{13}C NMR (125 MHz, $CDCl_3$) δ 189.21, 159.63, 155.17, 135.98, 130.26, 129.65, 128.84, 127.08, 123.90, 120.73, 118.58. The analytical data are consistent with published ones. [15]

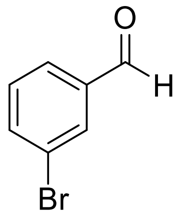

3-Bromobenzaldehyde (2t): Following the general procedure **GP1**, two batches of compound **1t** (0.2 mmol each one, 37.2 mg) were converted and after 35 h the reaction mixture was concentrated under reduced pressure. Product **2t** was purified by flash chromatography on silica using hexane/AcOEt 98:2 affording a yellow oil (44.4 mg, 60% yield). ^{1}H NMR (500 MHz, $CDCl_3$) δ 9.96 (s, 1H), 8.02 (s, 1H), 7.82 (d, *J* = 7.5Hz, 2H), 7.76 (d, *J* = 7.5Hz, 2H), 7.42 (t, *J* = 7.5Hz, 1H). ^{13}C NMR (125 MHz, $CDCl_3$) δ 190.89, 138.15, 137.46, 132.53, 130.78, 128.52, 123.53. The analytical data are consistent with published ones. [16]

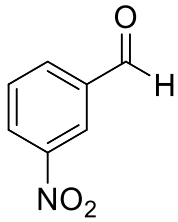

3-Nitrobenzaldehyde (2u): Following the general procedure **GP1**, two batches of compound **1u** (0.2 mmol each one, 30.6 mg) were converted and after 35 h the reaction mixture was concentrated under reduced pressure. Product **2u** was purified by flash chromatography on silica using hexane/AcOEt 95:5 affording a semi solid (35 mg, 58% yield) ^{1}H NMR (500 MHz, $CDCl_3$) δ 10.13 (s, 1H), 8.72-8.71 (m, 1H), 8.51-8.49 (m, 1H), 8.24 (dt, *J* = 8 Hz, 1.5 Hz, 1H), 7.77 (t, *J* = 7.5Hz, 1H). ^{13}C NMR (125 MHz, $CDCl_3$) δ 189.85, 137.55, 134.75, 130.53, 128.75, 124.67. The analytical data are consistent with published ones. [2]

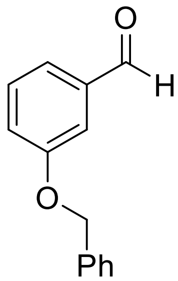

3-(Benzyloxy)benzaldehyde (2v): Following the general procedure **GP1**, two batches of compound **1v** (0.2 mmol each one, 42.8mg) were converted and after 35 h the reaction mixture was concentrated under reduced pressure. Product **2v** was purified by flash chromatography on

silica using hexane/AcOEt 95:5 affording a yellow semi solid (47.4 mg, 56%). ^{1}H NMR (500 MHz, $CDCl_3$) δ 9.98 (s, 1H), 7.48-7.35 (m, 9H), 5.13 (s, 2H). ^{13}C NMR (125 MHz, $CDCl_3$) δ 192.21, 159.47, 137.98, 136.45, 130.26, 128.82, 128.35, 127.69, 123.82, 122.34, 113.43, 70.38. The analytical data are consistent with published ones. [17]

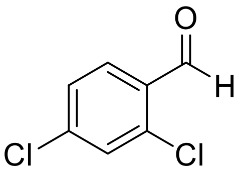

2,4-Dichlorobenzaldehyde (2w): Following the general procedure **GP1**, two batches of compound **1w** (0.2 mmol each one, 35.2 mg) were reacted and after 35 h the reaction mixture was concentrated under reduced pressure. Product **2w** was purified by flash chromatography on silica using hexane/AcOEt 98:2, followed by hexane/AcOEt 90:10 giving a white solid (49 mg, 70% yield). ^{1}H NMR (500 MHz, $CDCl_3$) δ 10.41 (s, 1H), 7.87 (d, *J* = 8.5 Hz, 1H), 7.48 (d, *J* = 2Hz, 1H), 7.39-7.36 (m, 1H). ^{13}C NMR (125 MHz, $CDCl_3$) δ 188.65, 141.25, 138.68, 131.08, 130.61, 130.47, 128.11. The analytical data are consistent with published ones. [18]

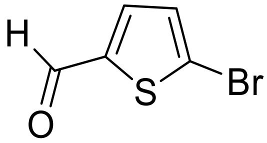

5-Bromothiophene-2-carbaldehyde (2x): Following the general procedure **GP1**, two batches of compound **1x** (0.2 mmol each one, 38.4 mg) were reacted and after 35 h the reaction mixture was concentrated under reduced pressure. Product **2x** was purified by flash chromatography on silica using hexane/AcOEt 98:2 followed by hexane/AcOEt 90:10 affording a brown oil (61 mg, 80% yield). ^{1}H NMR (500 MHz, $CDCl_3$) δ 9.78 (s, 1H), 7.52 (d, J = 4 Hz, 1H), 7.20 (d, J = 4 Hz, 1H). ^{13}C NMR (125 MHz, $CDCl_3$) δ 181.87, 145.36, 136.63, 131.59, 125.15. The analytical data are consistent with published ones. [4]

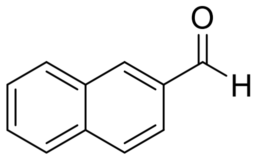

2-Naphthaldehyde (2y): Following the general procedure **GP1**, two batches of compound **1y** (0.2 mmol each one, 31.6 mg) were reacted and after 42 h the reaction mixture was concentrated under reduced pressure. Product **2y** was purified by flash chromatography on silica using hexane/AcOEt 95:5 affording a white solid (38 mg, 61% yield). ^{1}H NMR (500 MHz, $CDCl_3$) δ 10.17 (s, 1H), 8.35 (s, 1H), 8.02-7.90 (m, 4H), 7.66-7.58 (m, 2H). ^{13}C NMR (125 MHz, $CDCl_3$) δ 192.42, 136.61, 134.70, 134.27, 132.80, 129.68, 129.26, 129.25, 128.23, 127.24, 122.92. The analytical data are consistent with published ones. [4]

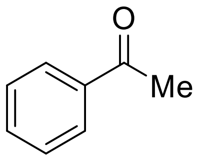

Acetophenone (2z): Following the general procedure **GP1**, two batches of alcohol **1z** (0.2 mmol each one, 24.4 mg) were oxidized and after 42 h the reaction mixture was concentrated under reduced pressure. Product **2z** was purified by flash chromatography on silica using hexane/AcOEt 96:4 affording an oil (28.8 mg, 60 % yield). ^{1}H NMR (500 MHz, $CDCl_3$) δ 7.96 (d, *J* = 7Hz, 2H), δ 7.56 (t, *J* = 7.5Hz, 1H), 7.47 (t, *J* = 7.5Hz, 2H), 2.61(s, 3H). ^{13}C NMR (125 MHz, $CDCl_3$) δ 198.32, 137.27, 133.24, 128.71, 128.45, 26.75. The analytical data are consistent with published ones. [4]

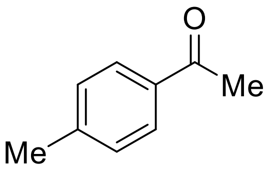

1-(*p*-Tolyl)ethan-1-one (2aa): Following the general procedure **GP1**, alcohol **1aa** (0.2 mmol each one, 27.2 mg) was converted and after 42 h the reaction mixture was concentrated under reduced pressure. Product **2aa** was purified by flash chromatography on silica using hexane/AcOEt 96:4 affording an oil (37.5 mg, 70% yield). ^{1}H NMR (500 MHz, $CDCl_3$) δ 7.87 (d, *J* = 8Hz 2H), 7.27 (d, *J* = 8Hz, 2H), 2.59 (s, 3H), 2.43 (s, 3H). ^{13}C NMR (125 MHz, $CDCl_3$)

δ 198.01, 144.01, 134.86, 129.38, 128.58, 26.66, 21.76. The analytical data are consistent with published ones. [19]

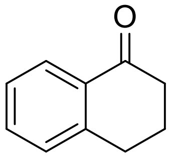

3,4-Dihydronaphthalen-1(2*H*)-one (2ab): Following the general procedure **GP1**, two batches of compound **1ab** (0.2 mmol each one, 29.6 mg) were reacted. After 42 h the reaction mixture was concentrated under reduced pressure. Product **2ab** was purified by flash chromatography on silica using hexane/AcOEt 95:5 affording a yellowish oil (32 mg, 55% yield). ^{1}H NMR (500 MHz, $CDCl_3$) δ 8.04-8.02 (m, 1H), 7.47 (td, *J* = 7.5Hz, 1.5 Hz, 1H), δ 7.31 (t, *J* = 7Hz, 1H), 7.26-7.24 (m, 1H), 2.97 (t, *J* = 6.5 Hz, 2H), 2.66 (t, *J* = 6.5Hz, 2H) 2.14 (quint, *J* = 6.5Hz, 2H). ^{13}C NMR (125 MHz, $CDCl_3$) δ 198.56, 144.63, 133.53, 132.76, 128.91, 127.31, 126.77, 39.31, 29.85, 23.42. The analytical data are consistent with published ones. [4]

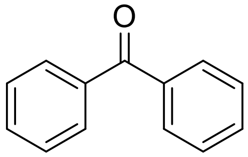

Benzophenone (2ac): Following the general procedure **GP1**, two batches of alcohol **1ac** (0.2 mmol each one, 36.8 mg) were photooxidized and after 42 h the reaction mixture was concentrated under reduced pressure. Product **2ac** was purified by flash chromatography on silica using hexane/AcOEt 95:5 affording a white solid (40.8 mg, 56% yield). ^{1}H NMR (500 MHz, $CDCl_3$) δ 7.82-7.80 (m, 4H), 7.61-7.58 (m, 2H), 7.50-7.47 (m, 4H). ^{13}C NMR (125 MHz, $CDCl_3$) δ 196.91, 137.74, 132.55, 130.20, 128.41. The analytical data are consistent with published ones. [3]

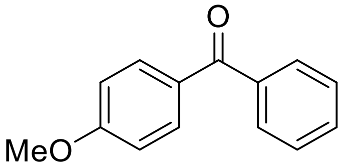

(4-Methoxyphenyl)(phenyl)methanone (2ad): Following the general procedure **GP1**, two batches of compound **1ad** (0.2 mmol each one, 42.8 mg) were photooxidized and after 42 h the reaction mixture was concentrated under reduced pressure. Product **2ad** was purified by flash chromatography on silica using hexane/AcOEt 95:5 affording a yellow semi solid

(43.2 mg, 51% yield). ^{1}H NMR (500 MHz, $CDCl_3$) δ 7.84-7.82 (m, 2H), 7.76-7.75 (m, 2H), 7.55 (m,1H), 7.49-7.46 (m, 2H), 6.98-6.95 (m, 2H), 3.89 (s, 3H). ^{13}C NMR (125 MHz, $CDCl_3$) δ 195.70, 163.36, 138.42, 132.69, 132.01, 130.29, 129.85, 128.31, 113.68, 55.62. The analytical data are consistent with published ones. [20]

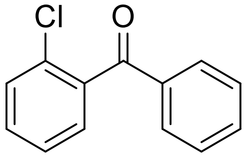

(2-Chlorophenyl)(phenyl)methanone (2ae): Following the general procedure **GP1**, two batches of compound **2ae** (0.2 mmol each one, 43.6 mg) were photooxidized and after 42 h the reaction mixture was concentrated under reduced pressure. Product **2ae** was purified by flash chromatography on silica using hexane/AcOEt 95:5 affording a white solid (45.8 mg, 53% yield). ^{1}H NMR (500 MHz, $CDCl_3$) δ 7.82-7.81 (m, 2H), 7.62-7.59 (m, 1H), 7.48-7.46 (m, 4H), 7.39-7.35 (m, 2H). ^{13}C NMR (125 MHz, $CDCl_3$) δ 195.43, 138.75, 136.61, 133.84, 131.45, 131.26, 130.21, 129.25, 128.75, 126.81. The analytical data are consistent with published ones. [20]

Mechanistic studies

ON/OFF experiment: A 10 mL glass vial was charged with 4-bromobenzyl alcohol (0.2 mmol), $CeCl_3$ (10 mol %), $NaHCO_3$ (10 mol %), trimethoxybenzene (0.2 mmol, internal standard), and a stirring bar and the glass vial was sealed with a PTFE septum. CH_3CN (3 mL) was added and the reaction was opened to air via a needle. The reactions were placed in a pre-programmed temperature controlled blue LED reactor (as shown in Figure S1) and the reaction mixture was irradiated with a 455 nm blue LED. To monitor the reaction progress, a small aliquot was removed after specific time intervals, concentrated under reduced pressure, and analyzed by ^{1}H NMR to determine the product yield.

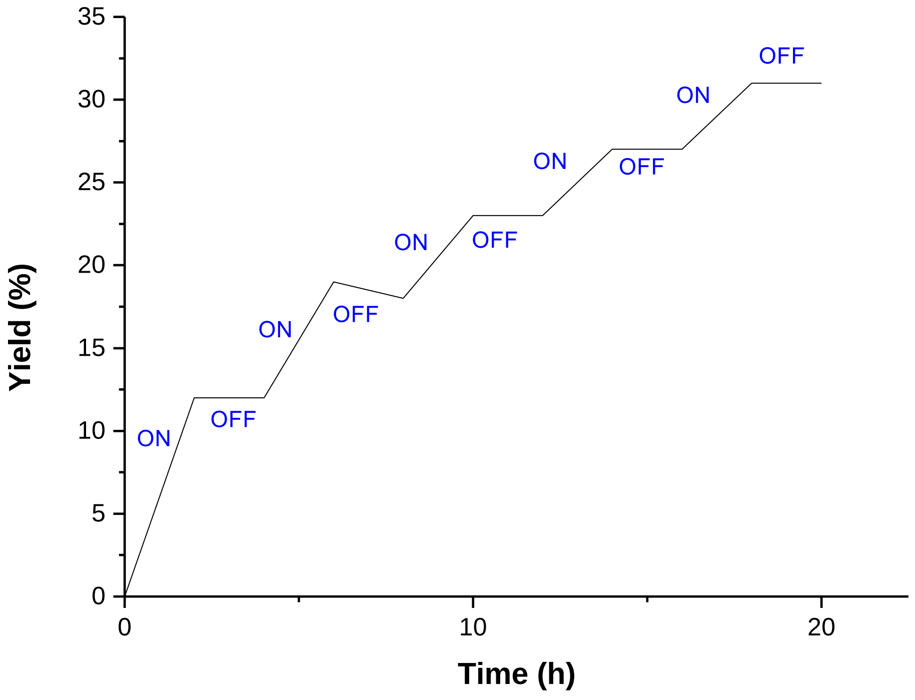

The reaction profile during alternating irradiation shows that the reaction does only proceed in the presence of light. This indicates that an essential reaction step requires light excitation. If radical chain processes are involved, the chain length is very short and does require constant initiation.

UV–vis experiments

In order to verify whether the interaction of substituted benzyl alcohols and cerium(IV) could lead to the overall LMCT process, which reduced the Ce(IV) species to Ce(III), a similar approach to the one reported by Zuo et al. was used.[21] (n-Bu_4)$_2Ce^{IV}Cl_6$ was chosen as a Ce(IV) source to ensure sufficient solubility in organic solvents and facilitate the detection of the species.

Synthesis of (n-Bu_4N)$_2Ce^{IV}Cl_6$

In a round-bottomed flask equipped with a teflon-coated stirring bar, tetrabutylammonium chloride (3.24 g, 11.7 mmol, 2.0 equiv) and $Ce(SO_4)_2{\cdot}(H_2O)_n$ (2.36 g, 5.8 mmol, 1.0 equiv) were placed, then HCl 37% (15 mL) was added at room temperature. After the formation of a yellow-orange precipitate, additional tetrabutylammonium chloride (324 mg, 1.2 mmol, 0.1 equiv) was added and the reaction additionally stirred for 20 minutes. The suspension was cooled to 5 °C using an ice–water bath, then the solid was collected by suction-filtration over a sintered funnel, the yellow-orange solid was washed three times with the minimal amount of acetone (approx. 10 mL each time) and dried under high vacuum, to afford an intensely yellow powder (480 mg, 0.57 mmol, 10% yield)

Preparation of a basic solution of (n-Bu_4N)$_2Ce^{IV}Cl_6$ in MeCN (solution A).

In a glass vial equipped with a teflon-coated stirring bar and a septum, (n-Bu_4N)$_2Ce^{IV}Cl_6$ (2.2 mg, 2.6 μmol) and $NaHCO_3$ (1.7 mg, 21 μmol) were dissolved in MeCN (4 mL, HPLC grade).

Preparation of $Ce^{IV}(OBn)Cl_n$ in MeCN (solution B).

In a glass vial equipped with a teflon-coated stirring bar and a septum, (n-Bu_4)$_2Ce^{IV}Cl_6$ (2.2 mg, 2.6 μmol) and $NaHCO_3$ (1.7 mg, 21 μmol) and **1e** (62 mg, 0.57 mmol) were dissolved in MeCN (4 mL, HPLC grade).

Experimental procedure and sampling

The UV–vis measurements were performed using a Shimadzu UV-3600 Vis–NIR spectrophotometer using a fluorescence cuvette (1 cm optical pathway, both faces can transmit light). A single blue LED OSRAM Oslon® SSL 80 royal-blue LEDs (λ_{max} = 455 nm (± 15 nm), 3.5 V, 700 mA), equipped with a metallic passive cooling element, was placed approx. 2 mm away from one transmitting side of the cuvette, at 90° from the measuring beam. The spectra were recorded in the 200–600 nm range.

In order to record the spectra, the corresponding previously prepared solution was transferred into a syringe, filtered using a syringe filter and transferred into the cuvette, closed with a PTFE stopper. The acquisition routine was started and after a certain time interval the illumination was started.

UV–vis spectra of $(n\text{-}Bu_4N)_2Ce^{IV}Cl_6$

Solution A was used to record the UV–vis spectra of $(n\text{-}Bu_4N)_2Ce^{IV}Cl_6$ in the absence of light.

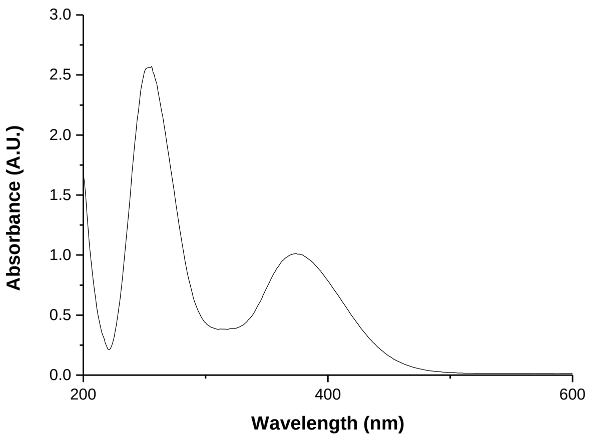

Figure S2: UV–vis spectrum of $(n\text{-}Bu_4N)_2Ce^{IV}Cl_6$ in the absence of light.

Spectra acquisition of $Ce^{IV}(OBn)Cl_n$ in the absence of light

Solution B was used and each spectrum was acquired after 50 s from the previous one. In the presence of benzyl alcohol (**1e**), the concentration of the Ce(IV) species remained almost constant without blue light irradiation (Figure S3). The small modulation was most likely caused by the fact that, in order to operate the spectroscopic device, absolute darkness could not be reached.

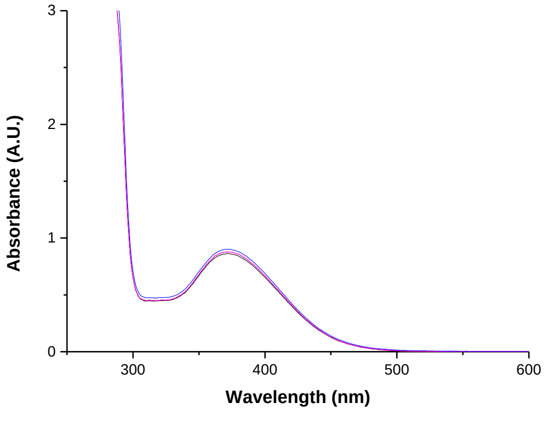

Figure S3: UV–vis spectrum of $Ce^{IV}(OBn)Cl_n$ in the absence of light.

Spectra acquisition of $Ce^{IV}(OBn)Cl_n$ in the presence of light

Solution B was used and each spectrum was acquired in an appropriate time interval. Upon irradiation with 455 nm light at different time intervals the reduction of the Ce(IV) species ($\lambda_{max} \approx 375$ nm) to Ce(III) species ($\lambda_{max} \approx 325$ nm) (Figure S4) was observed.

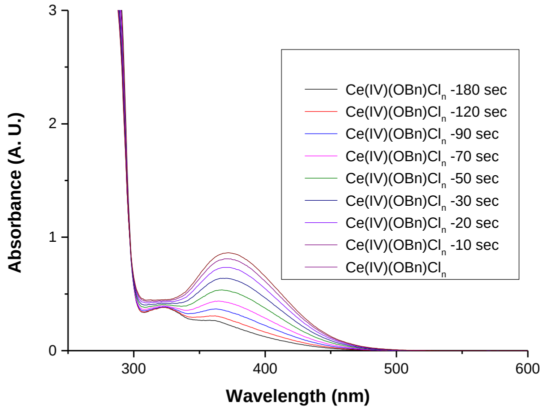

Figure S4: UV–vis spectra of $Ce^{IV}(OBn)Cl_n$ in the presence of light at different time intervals.

References

1. (a) M. F. Grünberg, L. J. Gooßen *Chem. Eur. J.*, **2013**, *19*, 7334-7337. (b) M. P. Paduraru, P. D. Wilson, *Org. Lett. 2003, 5*, 25, 4911–4913. (c) M. Lanzi, J. Merad, D. V. Boyarskaya, G. Maestri, C. Allain, G. MassonLanzi, *Org. Lett*. **2018**, *20*, 5247–5250.
2. M. Guan, C. Wang, J. Zhang, Y. Zhao, *RSC Adv.*, **2014**, *4*, 48777-48782
3. Y. Zhao, Y. Li, Z. Shen, X. Hu, B. Hu, L. Jin, N. M. Li. *Tetrahedron Lett.*, **2019**, *60*, 150994.
4. A. H. Bansode, G. Suryavanshi, *RSC Adv*. **2018**, *8*, 32055-32062.
5. Y. Kon,T. Nakashima, A. Yada, T. Fujitani, S. Onozawa, S. Kobayashi, K. Sato. *Org. Bio. Chem*, **2021**, *19*, 1115-1121.
6. P. -F. Wei, M. -Z, Qi, Z. -P. Wang, S. -Y. Ding, W. Yu, Q. Liu, L. -K. Wang, H.-Z. Wang, W. -K. An, W. Wang *J. Am. Chem. Soc*. **2018**, *140*, 4623–4631
7. D. Sirbu, C. Turta, A C. Benniston, F. Abou-Chahine, H. Lemmetyinen, N. V. Tkachenko , C. Woodd , E. Gibson. *RSC Adv*. **2014**, *4*, 22733-22742.
8. S. Vermaa, R. B. N. Baiga, M. N. Nadagoudab, R. S. Varmaa *ACS Sustain. Chem. Eng*. **2016**, *4*, 2333-2336.
9. C. Gangadurai, G. T. illa, D. S. Reddy *Org. Bio. Chem*, **2020**, *18,* 8459-8466.
10. T. Lim, J. Y. Ryoo, M. Janga, M. S. Han, *Org. Bio. Chem*, **2021**, *19,* 1009-1016.
11. S. Senthilkumar, W. Zhong, M. Natarajan, C. Lu, B. Xu, X. Liu, *New J. Chem.* **2021**, *45*, 705-713
12. S. Shirase, K. Shinohara, H. Tsurugi, K. Mashima, *ACS Catal*. **2018**, *8*, 6939−6947
13. S.Wertz ,D.Leifert, A.Studer. *Org. Lett.*, **2013**, *15*, 928-931.
14. Andres, Miriam et al. *Bioorg. Med. Chem. Lett*. **2014**, *24*, 5111-5117.
15. Honghua Rao and Chao-Jun Li, *Angew. Chem., Int. Ed.*, **2011**, *50*, 8936-8939.
16. G-F. Zha, W-Y. Fang, J. Leng, H-Li. Qin. *Adv, Synth. Catal*. **2019**, *361*, 2262-2267.
17. P. H. Gilmartin, M. C. Kozlowski *Org. Lett.* **2020**, *22*, 2914–2919
18. B. Hong, K. C. Aganda, A. Lee. *Org. Lett*, **2020**, *22*, 4395-4399.
19. S. Saha, S. Yadav, N. U Reshi, I. Dutta, S. Kunnikuruvan, J. K. Bera. *ACS Catalysis*, **2020**, *10*, 11385-11393.
20. X. Zhu, C. Liu, Y. Liu, H.Yang,H. Fu *Chem. Commun*. **2020**, *56*, 12443-12446.
21. A. Hu, J.-J. Guo, H. Pan, H. Tang, Z. Gao, Z. Zuo, *J. Am. Chem. Soc*. **2018**, *140,* 1612−1616.

NMR spectra

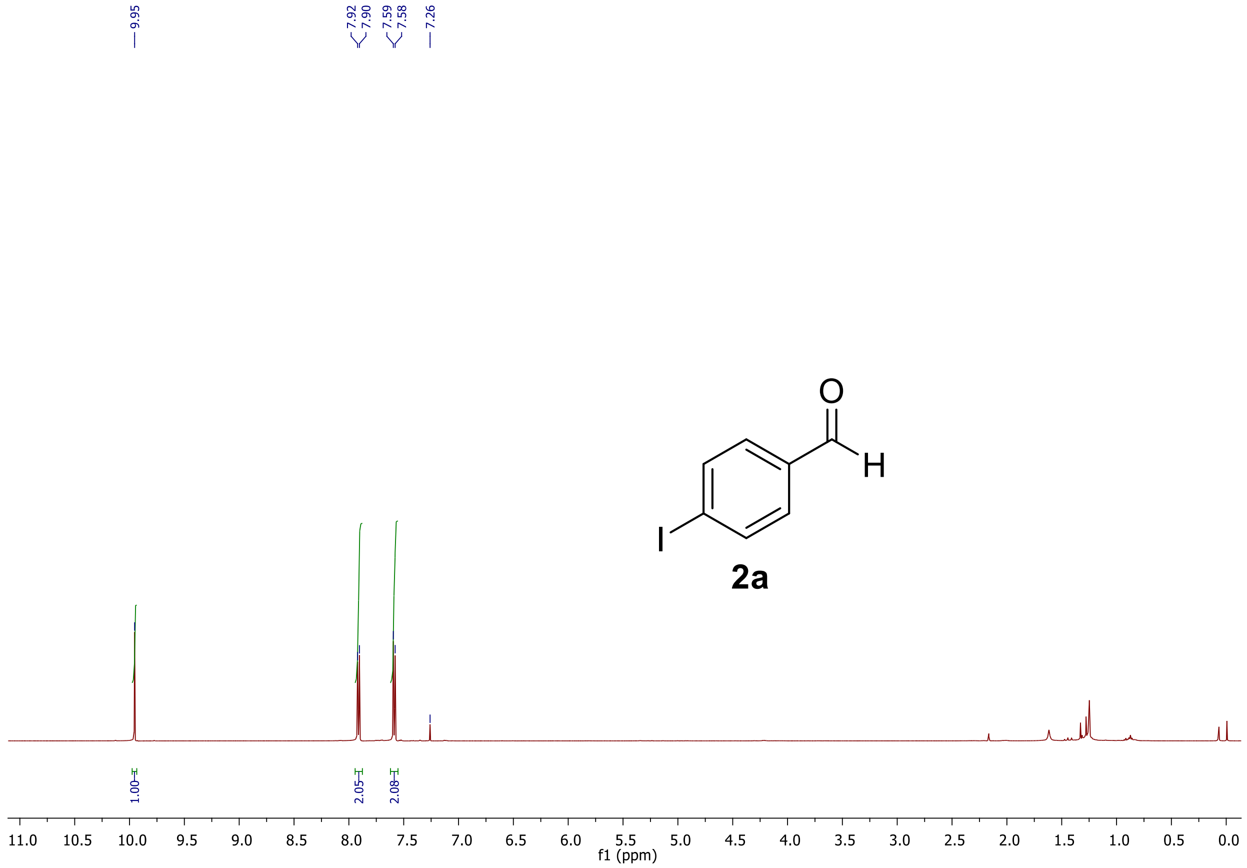
9.95
7.92
7.90
7.59
7.58
7.26
O
H
I
2a
1.00
2.05
2.08
11.0 10.5 10.0 9.5 9.0 8.5 8.0 7.5 7.0 6.5 6.0 5.5 5.0 4.5 4.0 3.5 3.0 2.5 2.0 1.5 1.0 0.5 0.0
f1 (ppm)

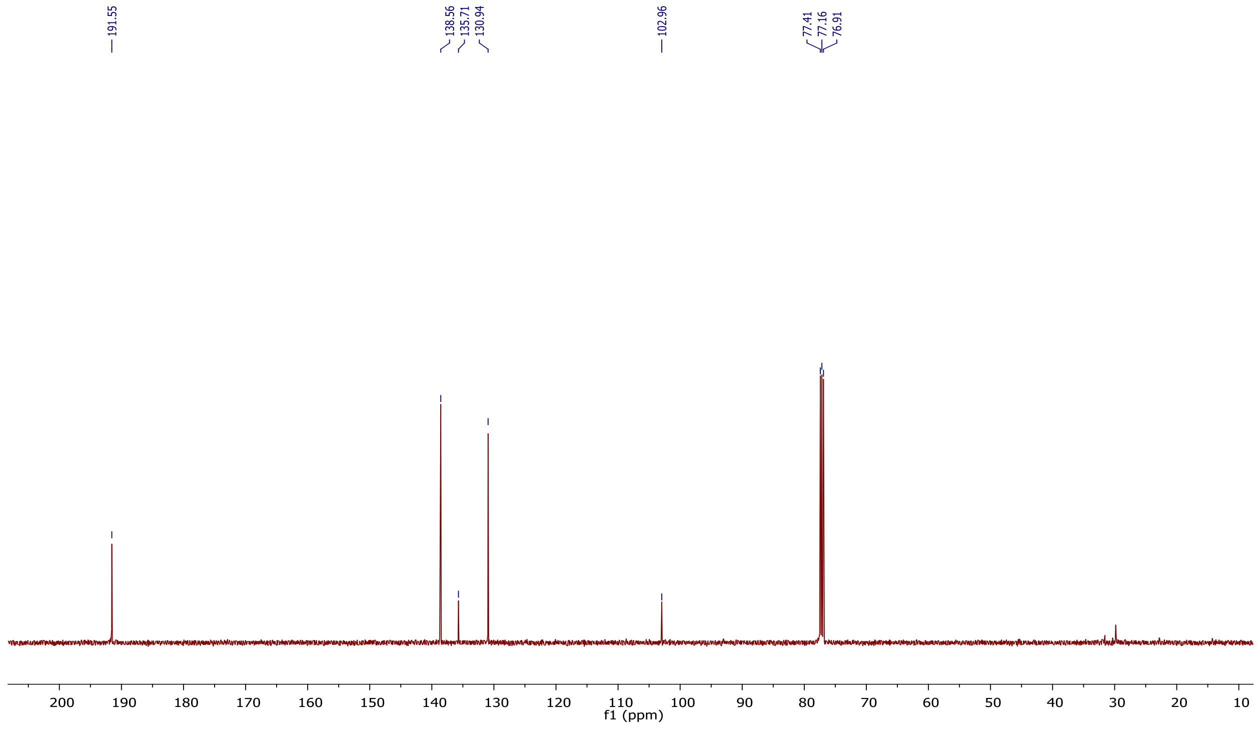
191.55
138.56
135.71
130.94
102.96
77.41
77.16
76.91
200 190 180 170 160 150 140 130 120 110 100 90 80 70 60 50 40 30 20 10
f1 (ppm)

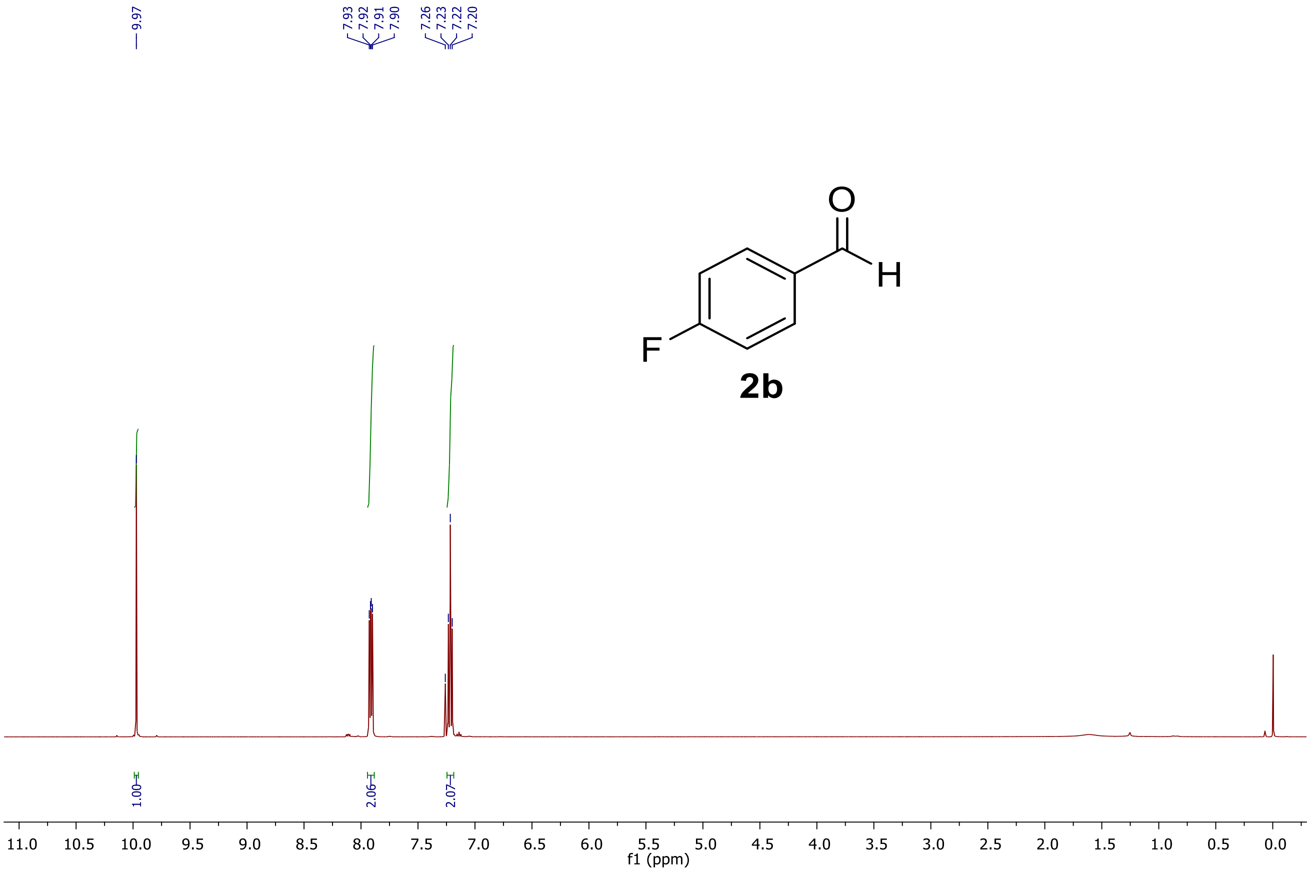
O
H
F
2b
9.97
7.93
7.92
7.91
7.90
7.26
7.23
7.22
7.20
1.00
2.06
2.07
11.0 10.5 10.0 9.5 9.0 8.5 8.0 7.5 7.0 6.5 6.0 5.5 5.0 4.5 4.0 3.5 3.0 2.5 2.0 1.5 1.0 0.5 0.0
f1 (ppm)

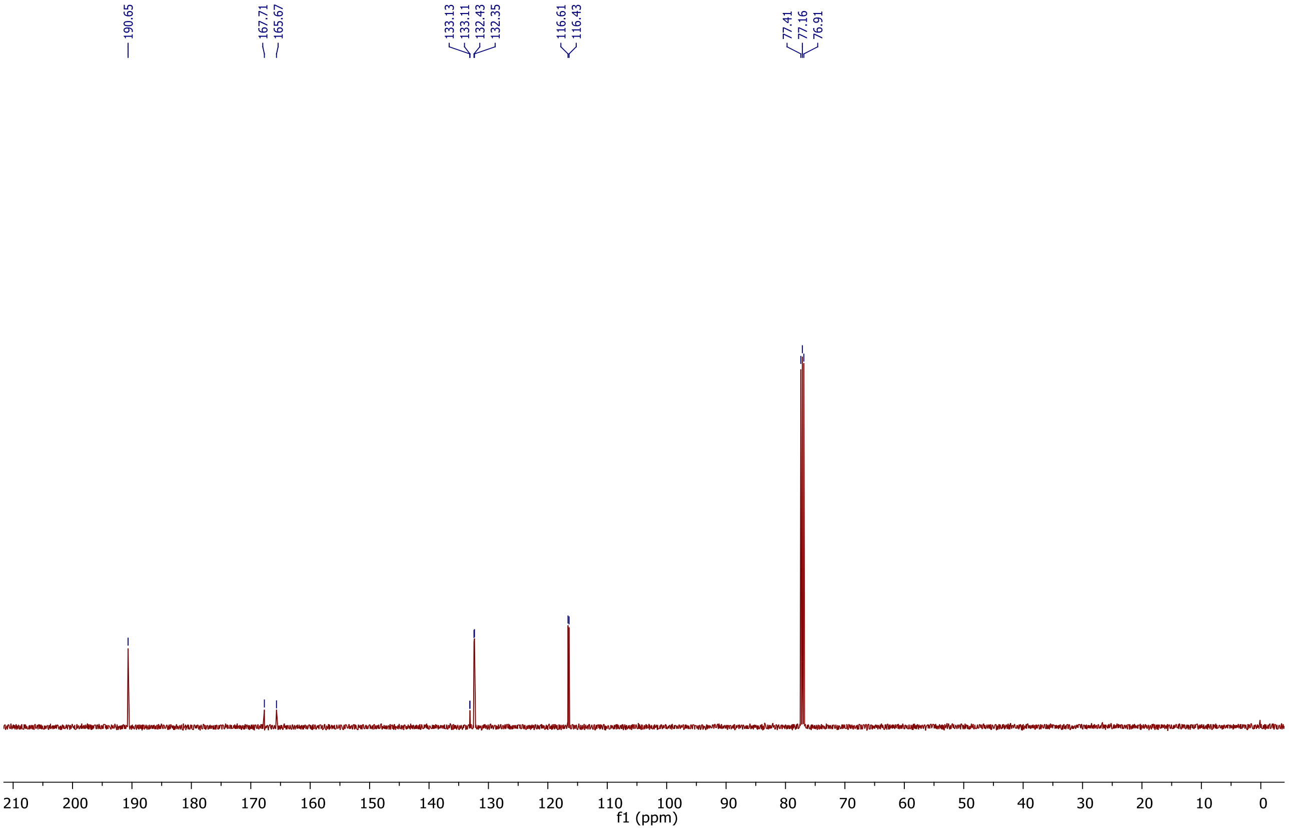
190.65
167.71
165.67
133.13
133.11
132.43
132.35
116.61
116.43
77.41
77.16
76.91
210 200 190 180 170 160 150 140 130 120 110 100 90 80 70 60 50 40 30 20 10 0
f1 (ppm)

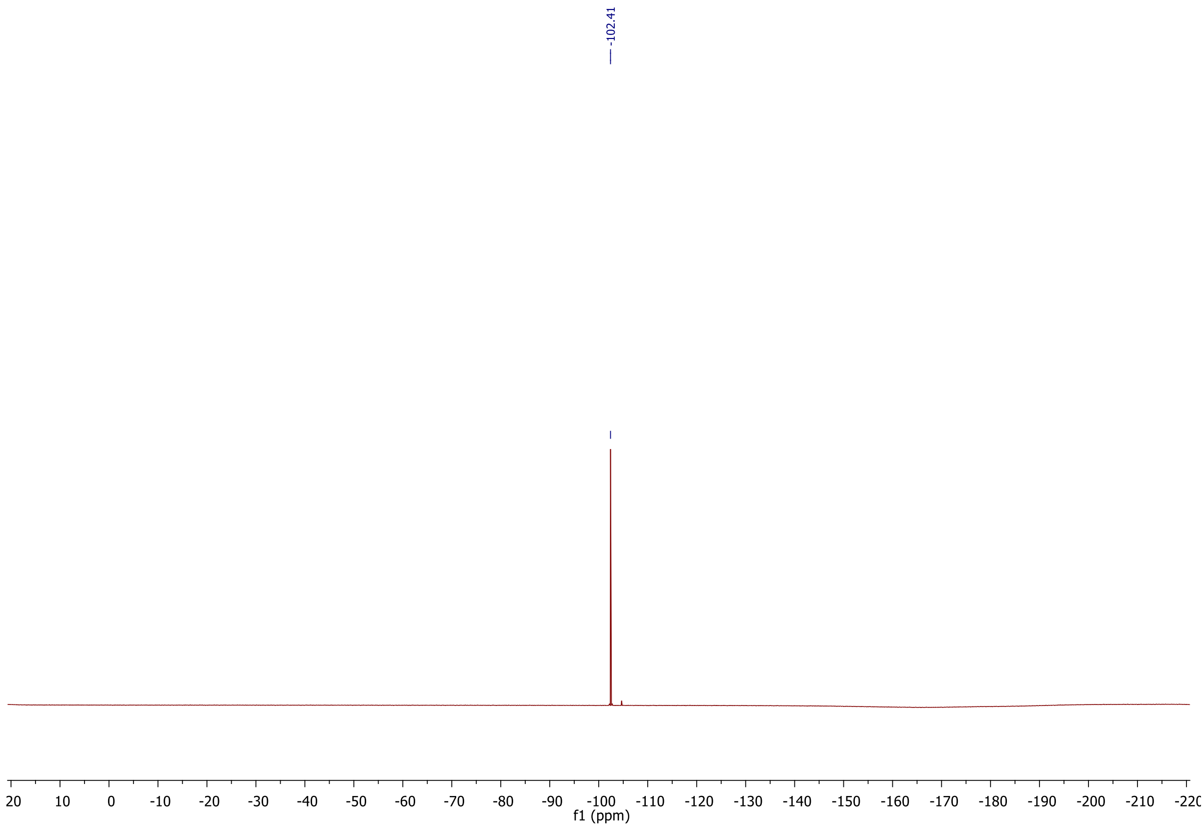
-102.41
20 10 0 -10 -20 -30 -40 -50 -60 -70 -80 -90 -100 -110 -120 -130 -140 -150 -160 -170 -180 -190 -200 -210 -220
f1 (ppm)

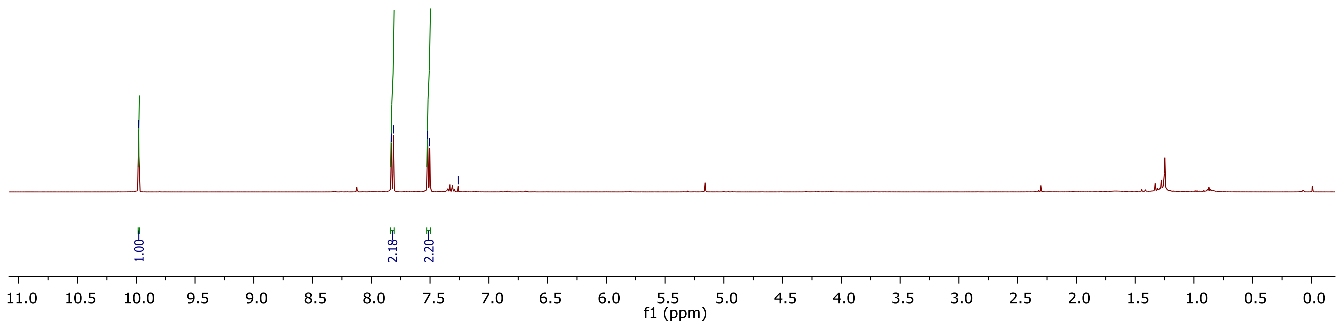
9.98
7.83
7.81
7.52
7.50
7.26
1.00
2.18
2.20
f1 (ppm)

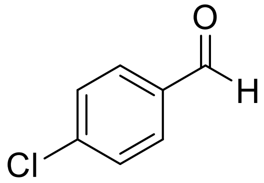
O
H
Cl

2c

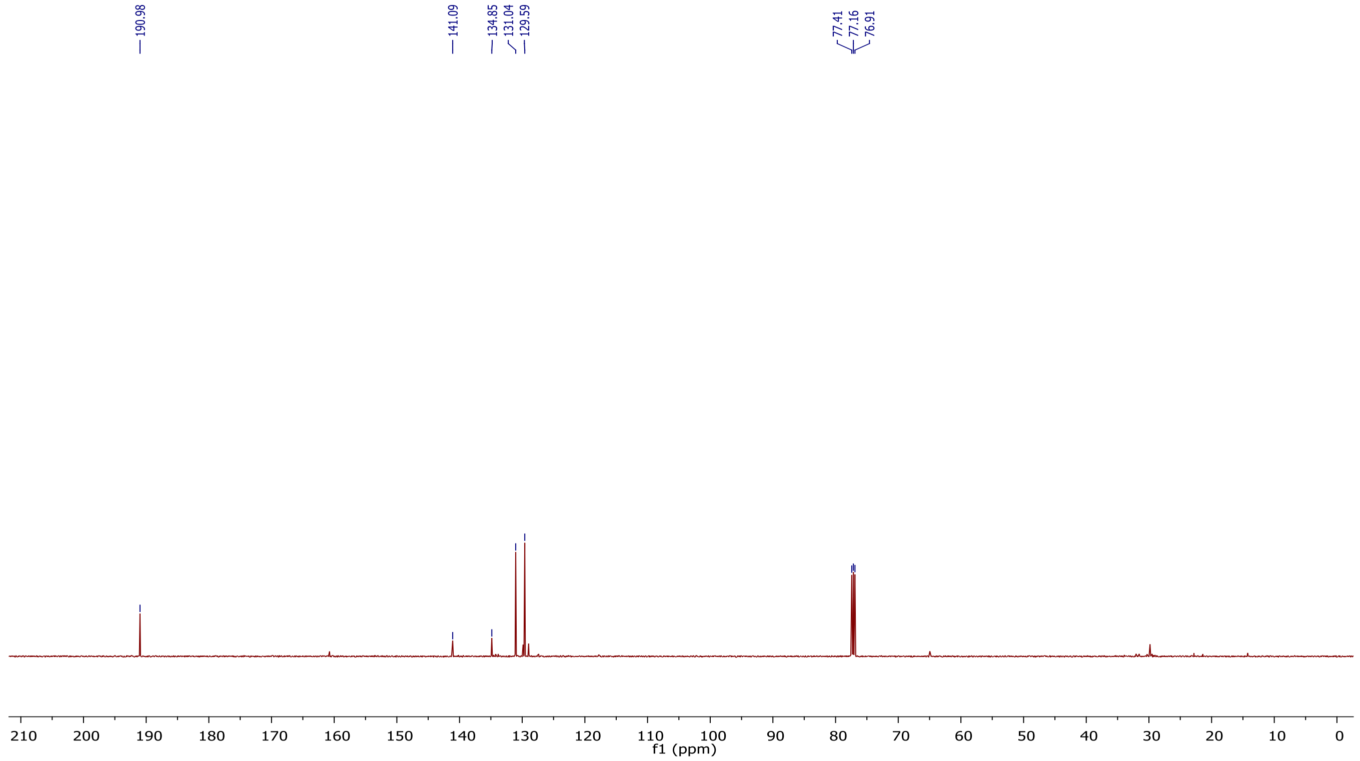
190.98
141.09
134.85
131.04
129.59
77.41
77.16
76.91
f1 (ppm)

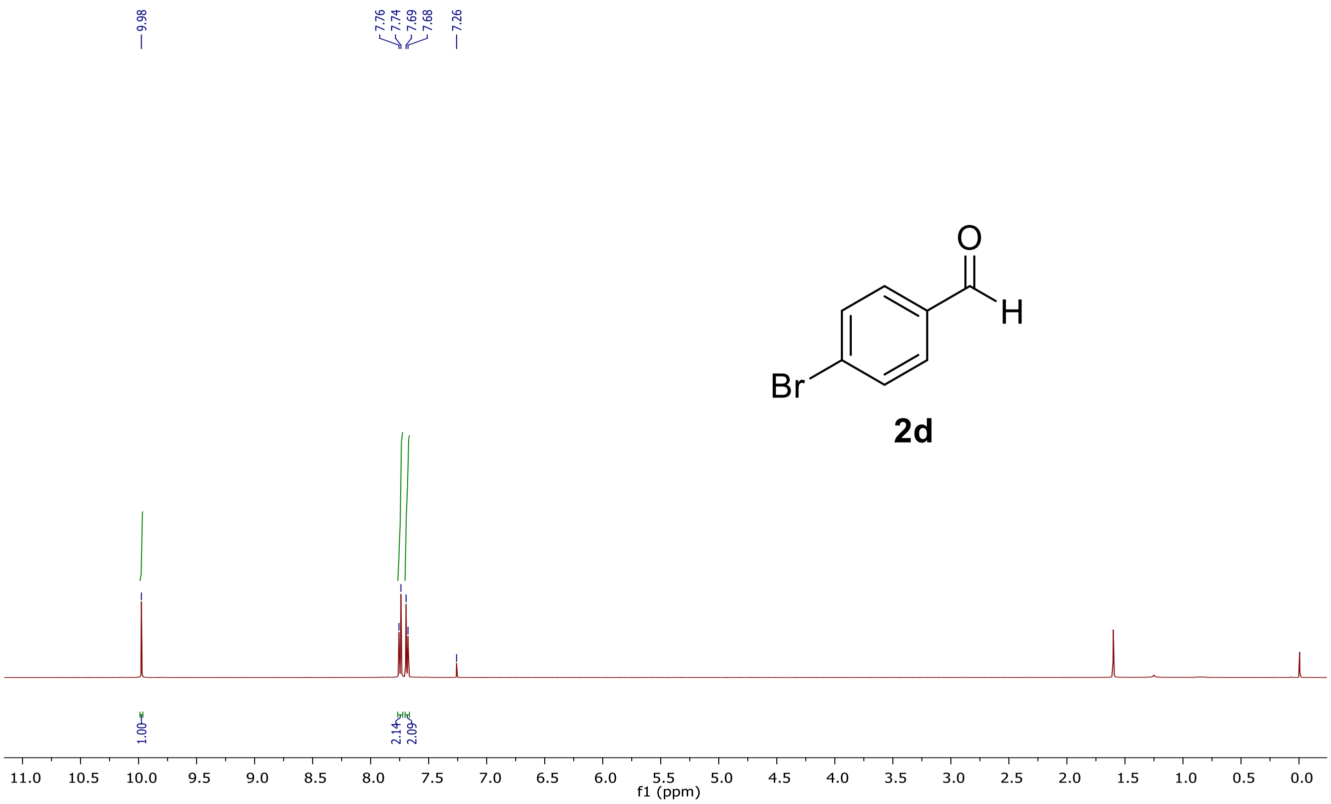
9.98
7.76
7.74
7.69
7.68
7.26
Br
O
H
1.00
2.14
2.09
f1 (ppm)

2d

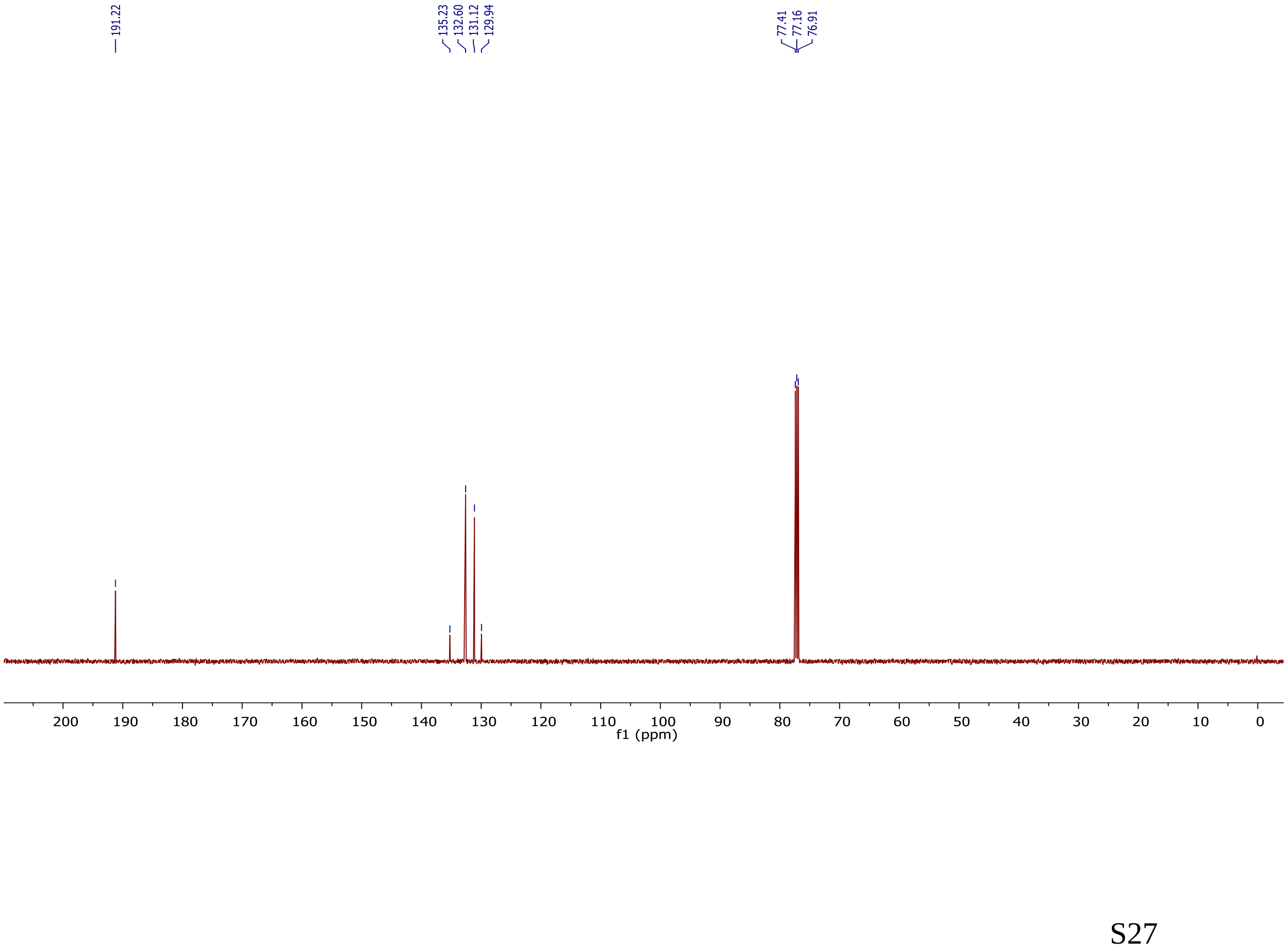
191.22
135.23
132.60
131.12
129.94
77.41
77.16
76.91
f1 (ppm)

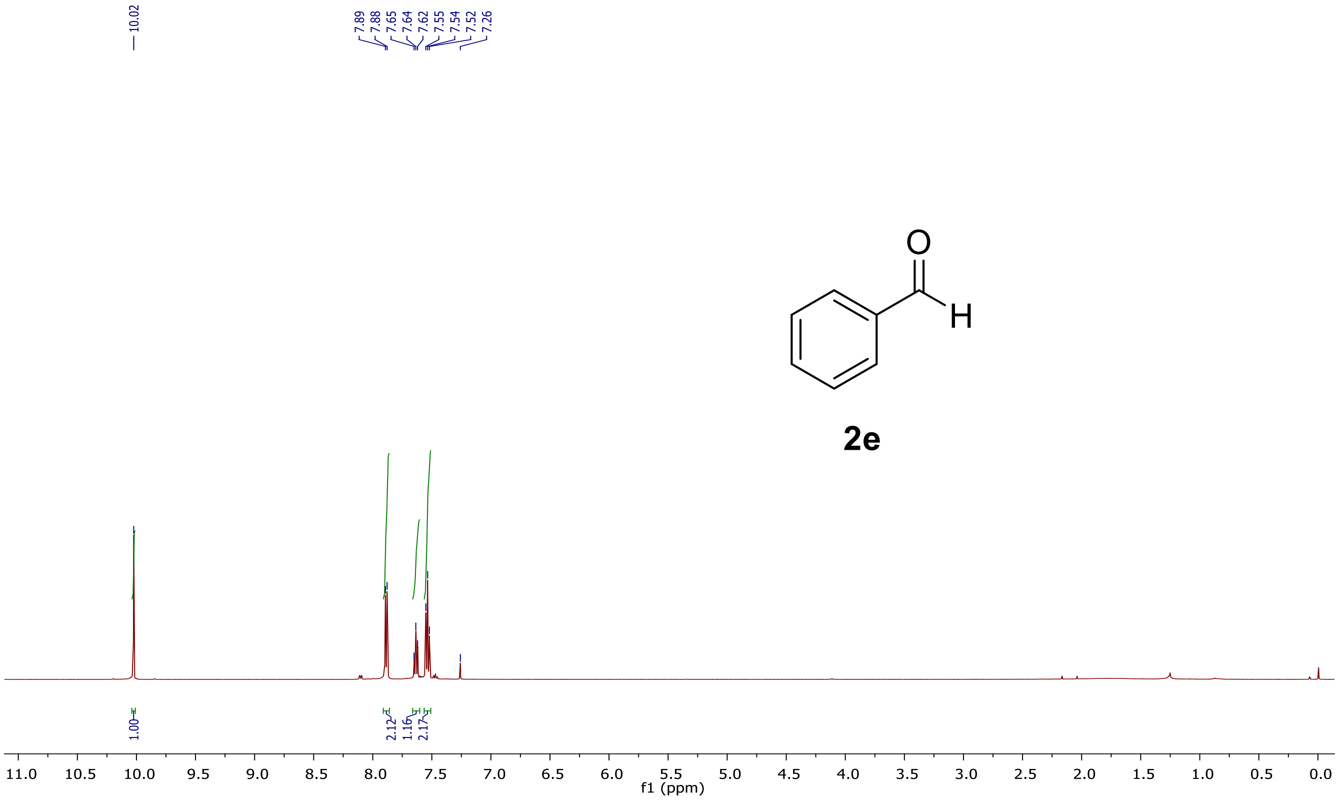
2e
10.02
7.89
7.88
7.65
7.64
7.62
7.55
7.54
7.52
7.26
1.00
2.12
1.16
2.17
11.0 10.5 10.0 9.5 9.0 8.5 8.0 7.5 7.0 6.5 6.0 5.5 5.0 4.5 4.0 3.5 3.0 2.5 2.0 1.5 1.0 0.5 0.0
f1 (ppm)

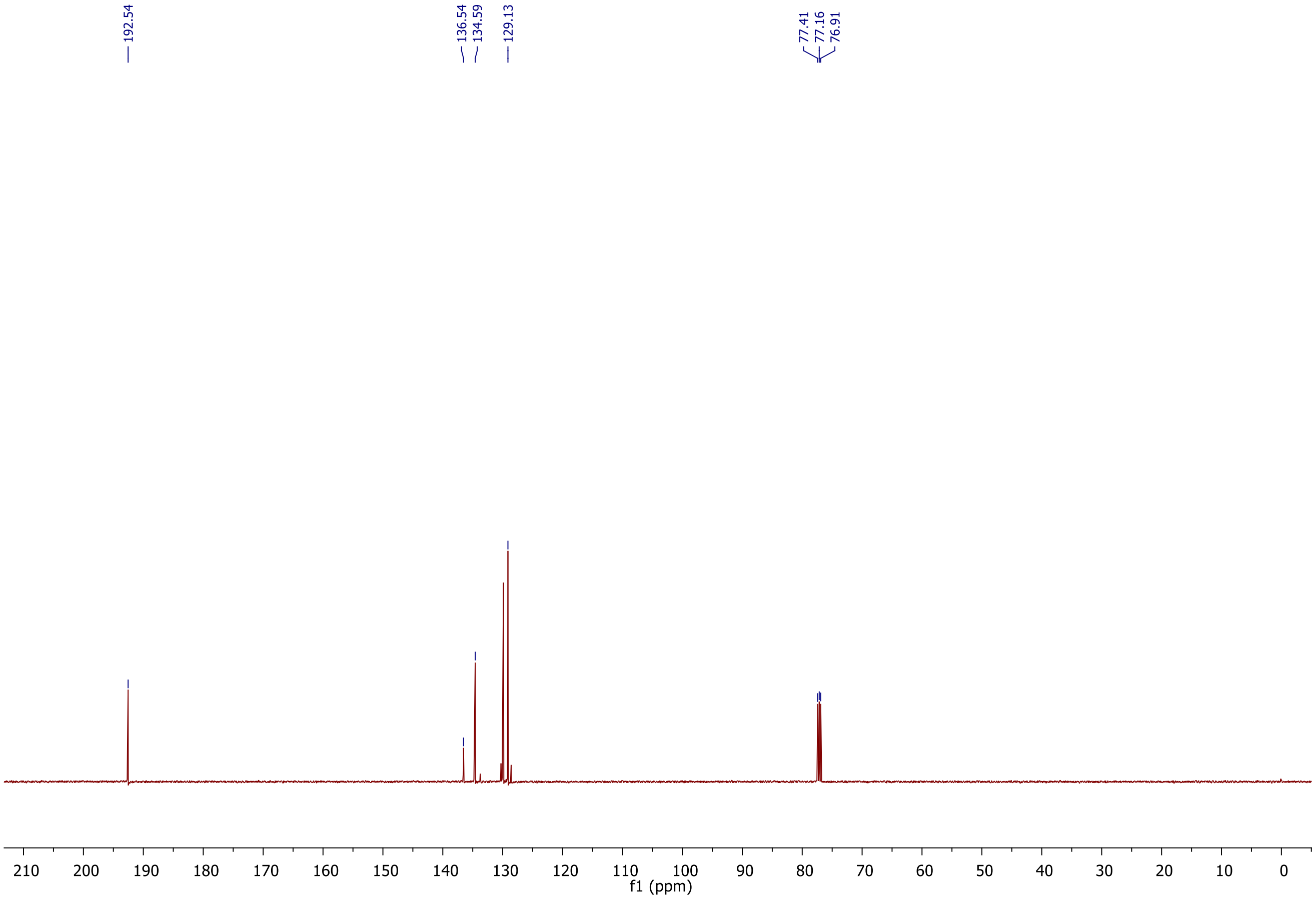
192.54
136.54
134.59
129.13
77.41
77.16
76.91
210 200 190 180 170 160 150 140 130 120 110 100 90 80 70 60 50 40 30 20 10 0
f1 (ppm)

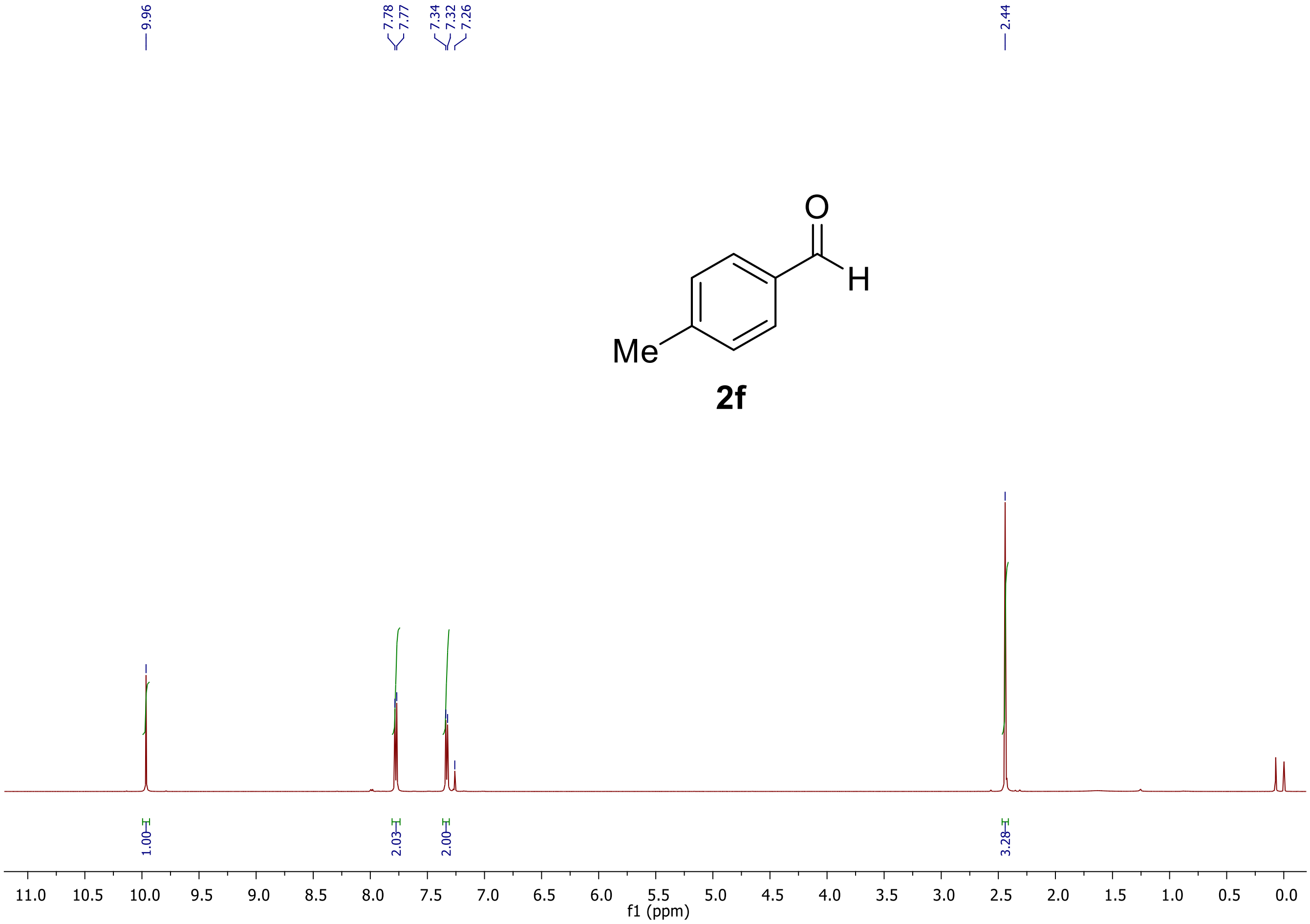
9.96
7.78
7.77
7.34
7.32
7.26
2.44
O
H
Me
1.00
2.03
2.00
3.28
f1 (ppm)

2f

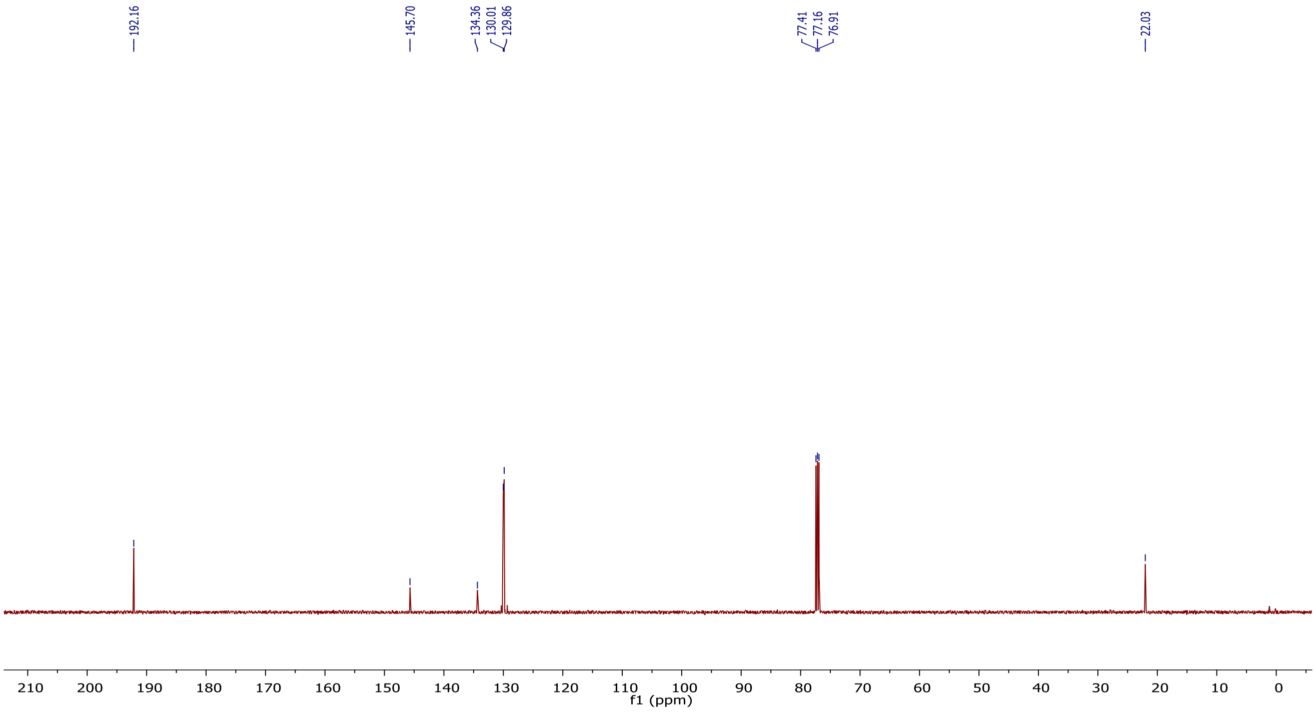
192.16
145.70
134.36
130.01
129.86
77.41
77.16
76.91
22.03
f1 (ppm)

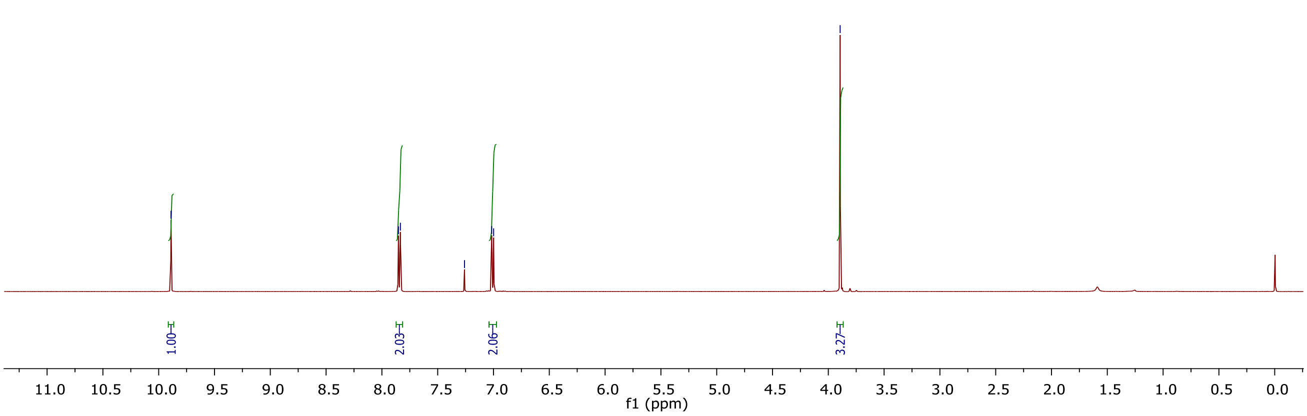

9.89
7.85
7.83
7.26
7.02
7.00
3.89
1.00
2.03
2.06
3.27
f1 (ppm)

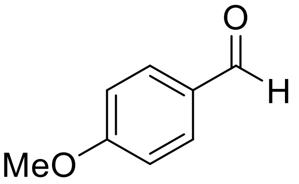

2g

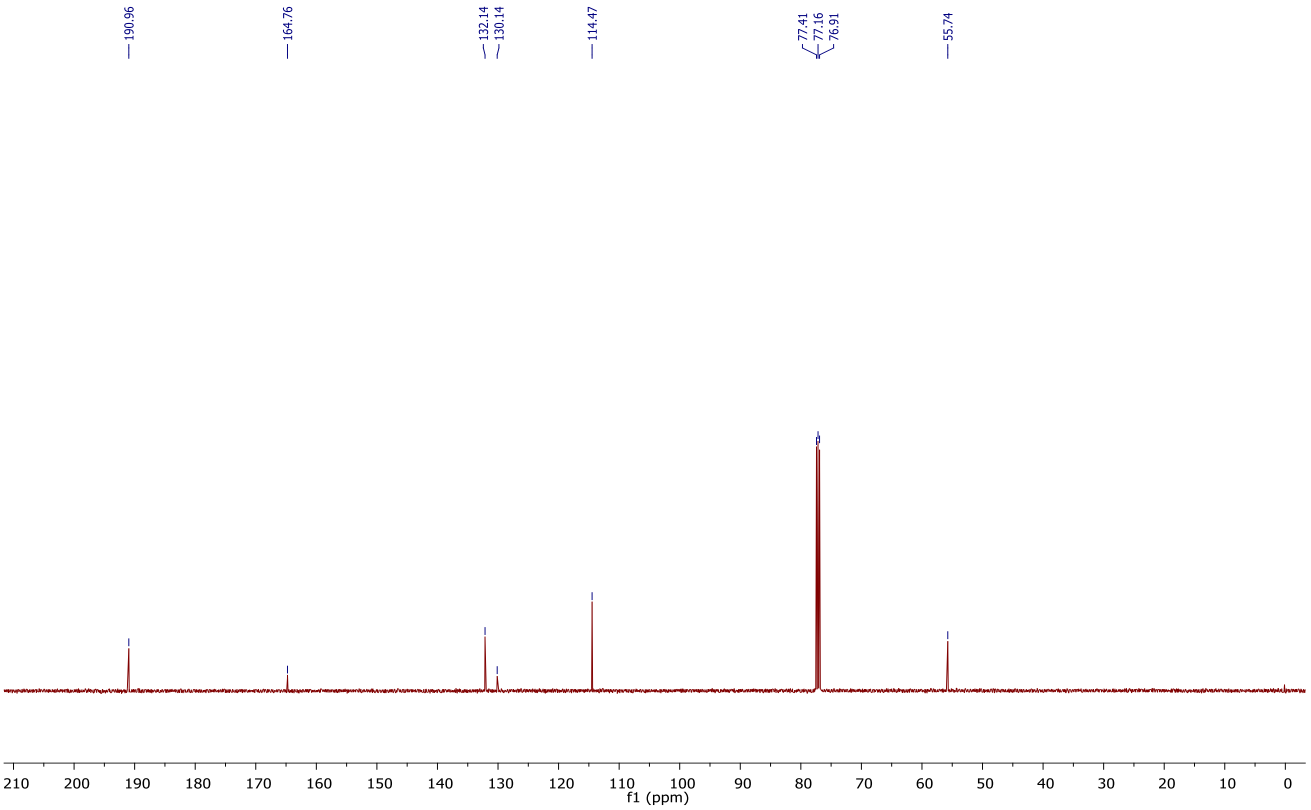

190.96
164.76
132.14
130.14
114.47
77.41
77.16
76.91
55.74
f1 (ppm)

2h

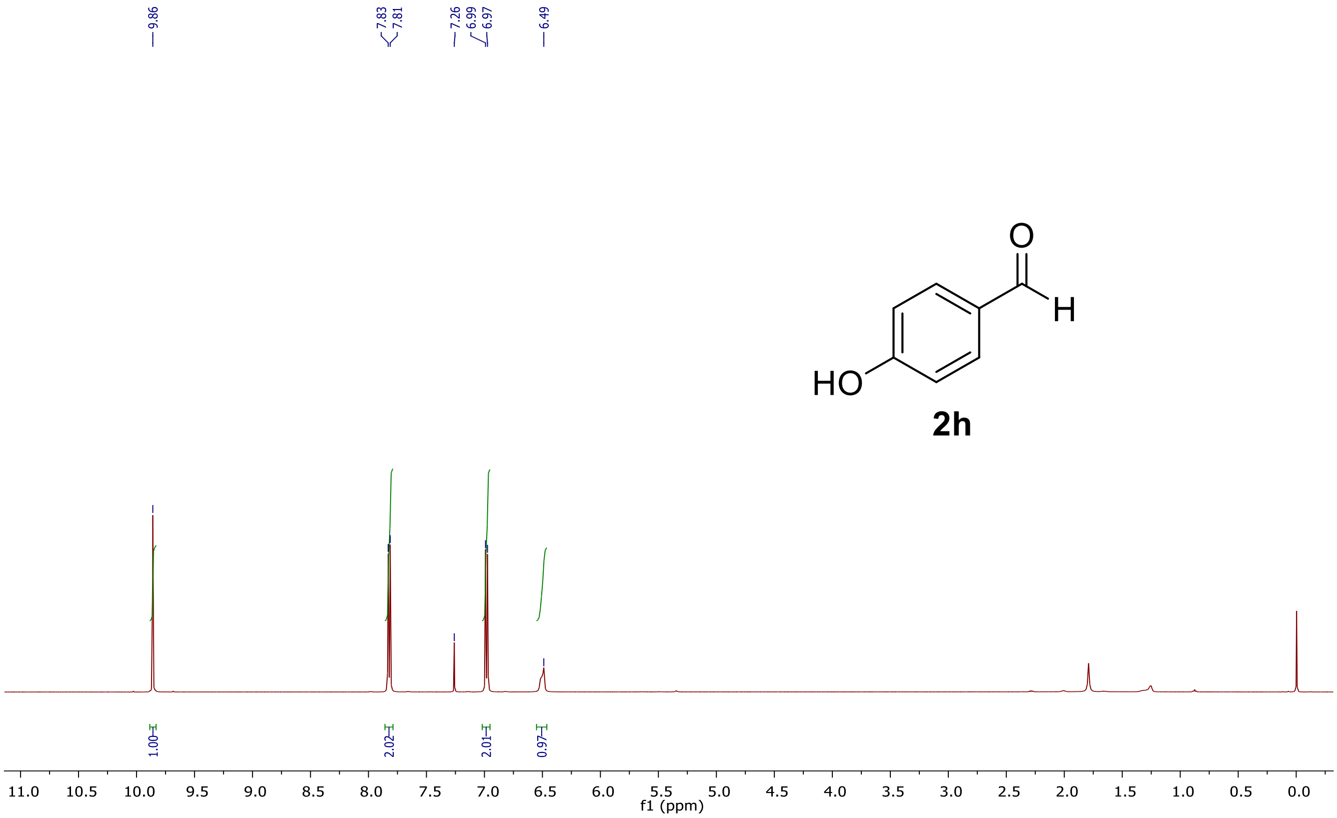

9.86
7.83
7.81
7.26
6.99
6.97
6.49
1.00
2.02
2.01
0.97
11.0 10.5 10.0 9.5 9.0 8.5 8.0 7.5 7.0 6.5 6.0 5.5 5.0 4.5 4.0 3.5 3.0 2.5 2.0 1.5 1.0 0.5 0.0
f1 (ppm)

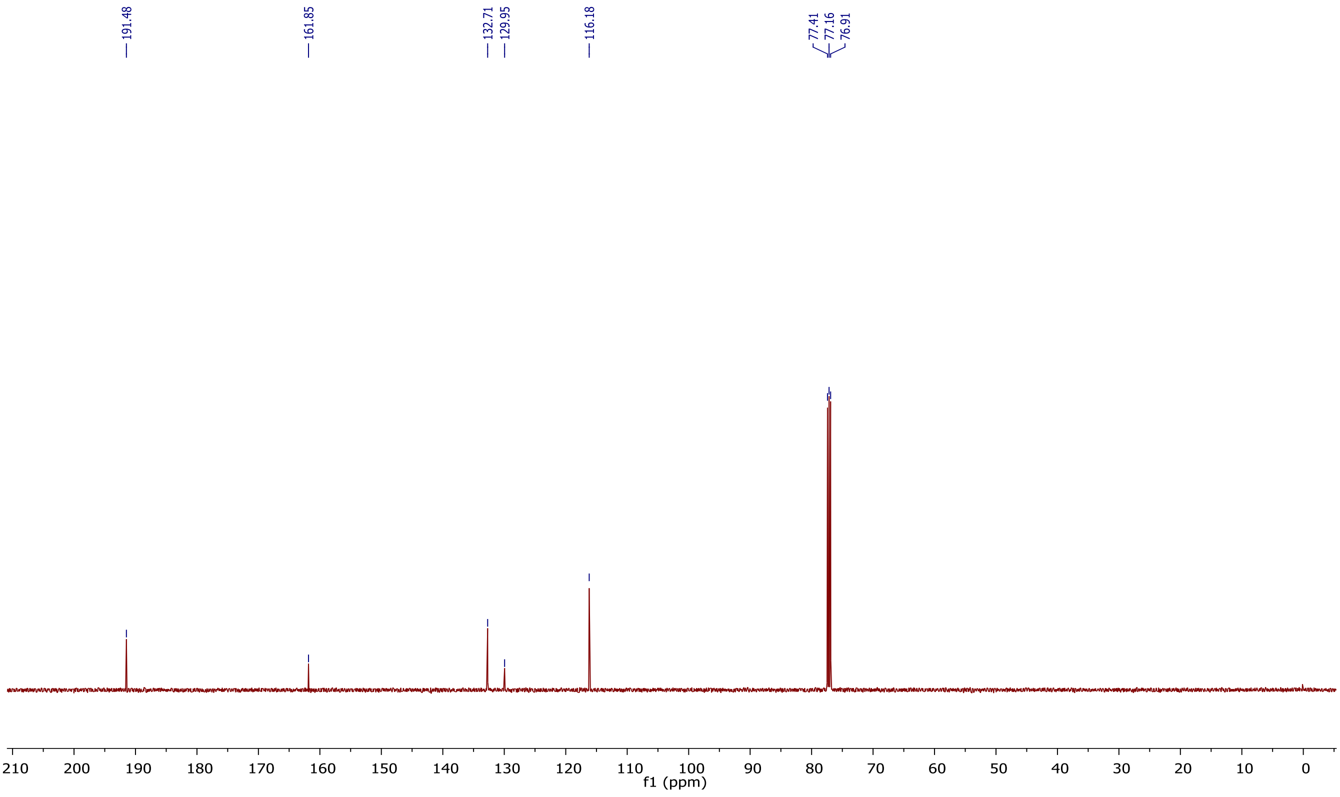

191.48
161.85
132.71
129.95
116.18
77.41
77.16
76.91
210 200 190 180 170 160 150 140 130 120 110 100 90 80 70 60 50 40 30 20 10 0
f1 (ppm)

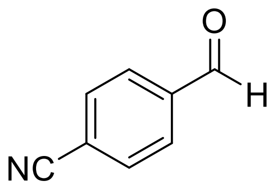
O
H
NC

2i

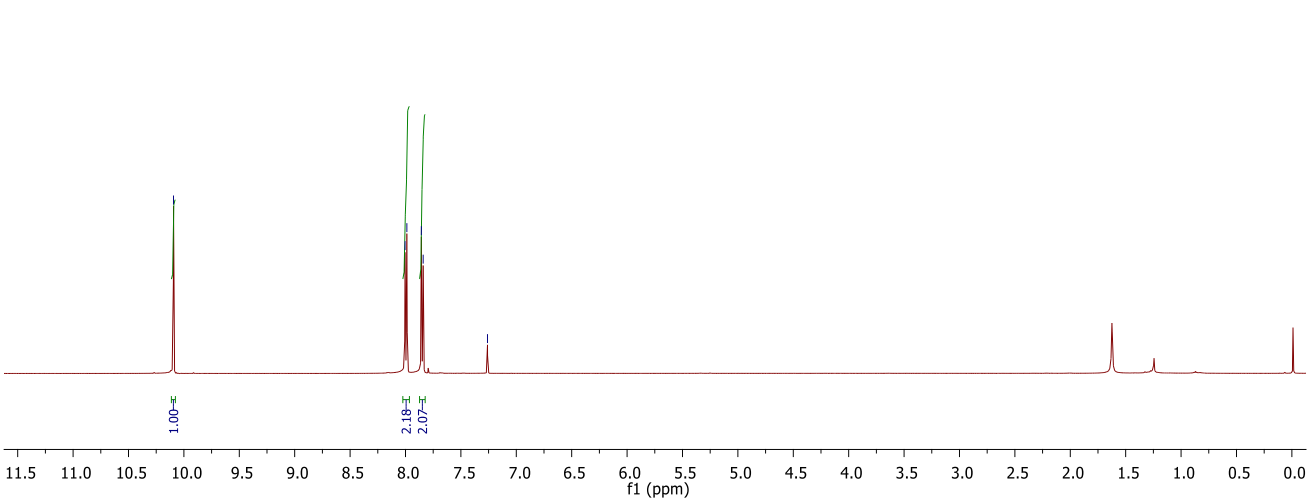
10.09
8.00
7.99
7.86
7.84
7.26
1.00
2.18
2.07
11.5 11.0 10.5 10.0 9.5 9.0 8.5 8.0 7.5 7.0 6.5 6.0 5.5 5.0 4.5 4.0 3.5 3.0 2.5 2.0 1.5 1.0 0.5 0.0
f1 (ppm)

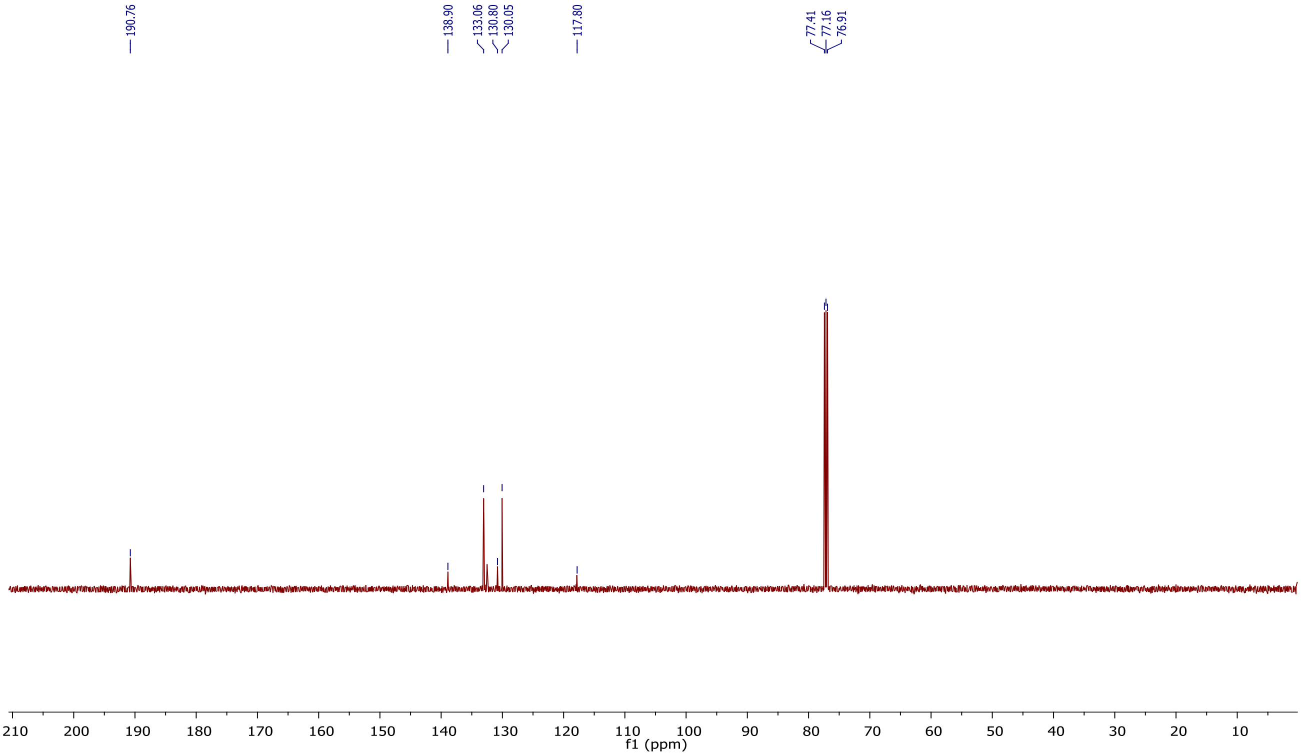
190.76
138.90
133.06
130.80
130.05
117.80
77.41
77.16
76.91
210 200 190 180 170 160 150 140 130 120 110 100 90 80 70 60 50 40 30 20 10
f1 (ppm)

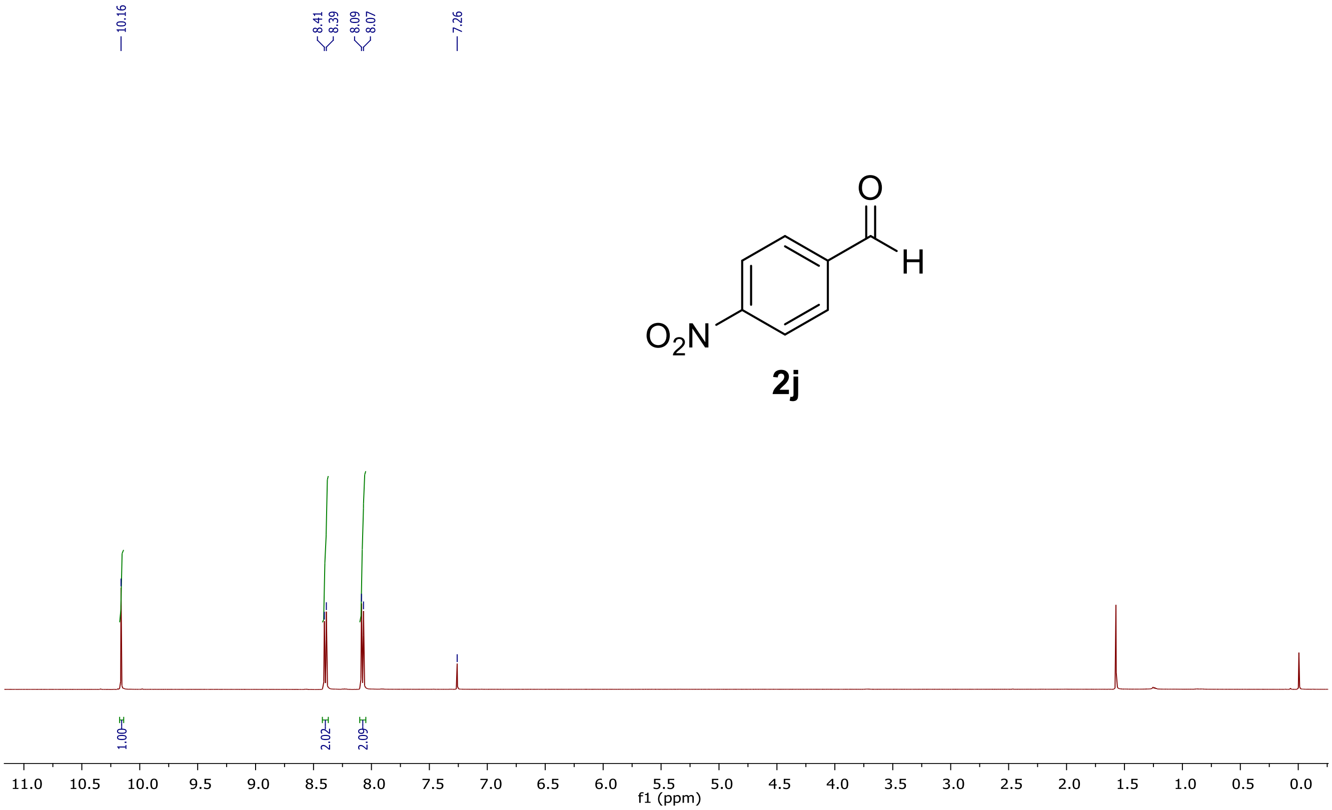
10.16
8.41
8.39
8.09
8.07
7.26
O
H
O2N
2j
1.00
2.02
2.09
11.0 10.5 10.0 9.5 9.0 8.5 8.0 7.5 7.0 6.5 6.0 5.5 5.0 4.5 4.0 3.5 3.0 2.5 2.0 1.5 1.0 0.5 0.0
f1 (ppm)

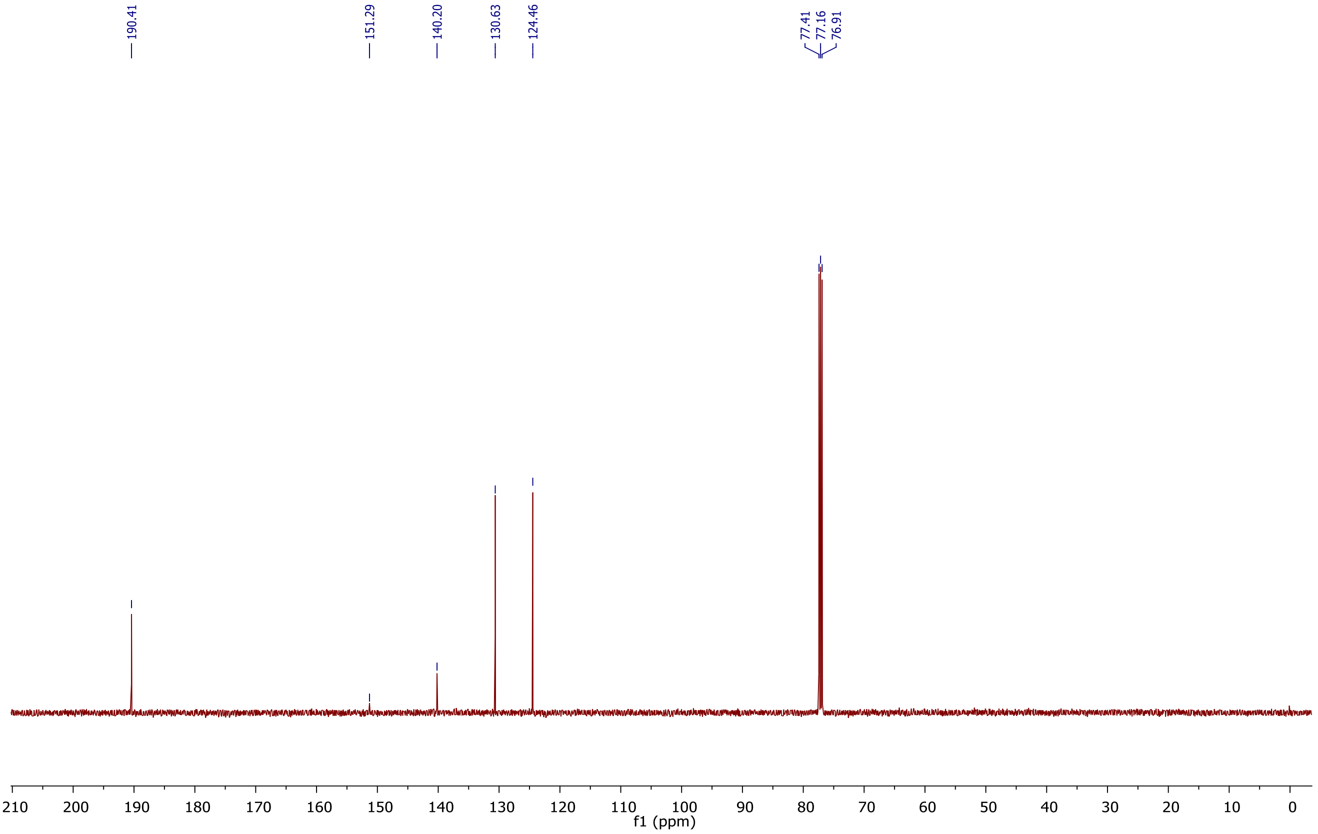
190.41
151.29
140.20
130.63
124.46
77.41
77.16
76.91
210 200 190 180 170 160 150 140 130 120 110 100 90 80 70 60 50 40 30 20 10 0
f1 (ppm)

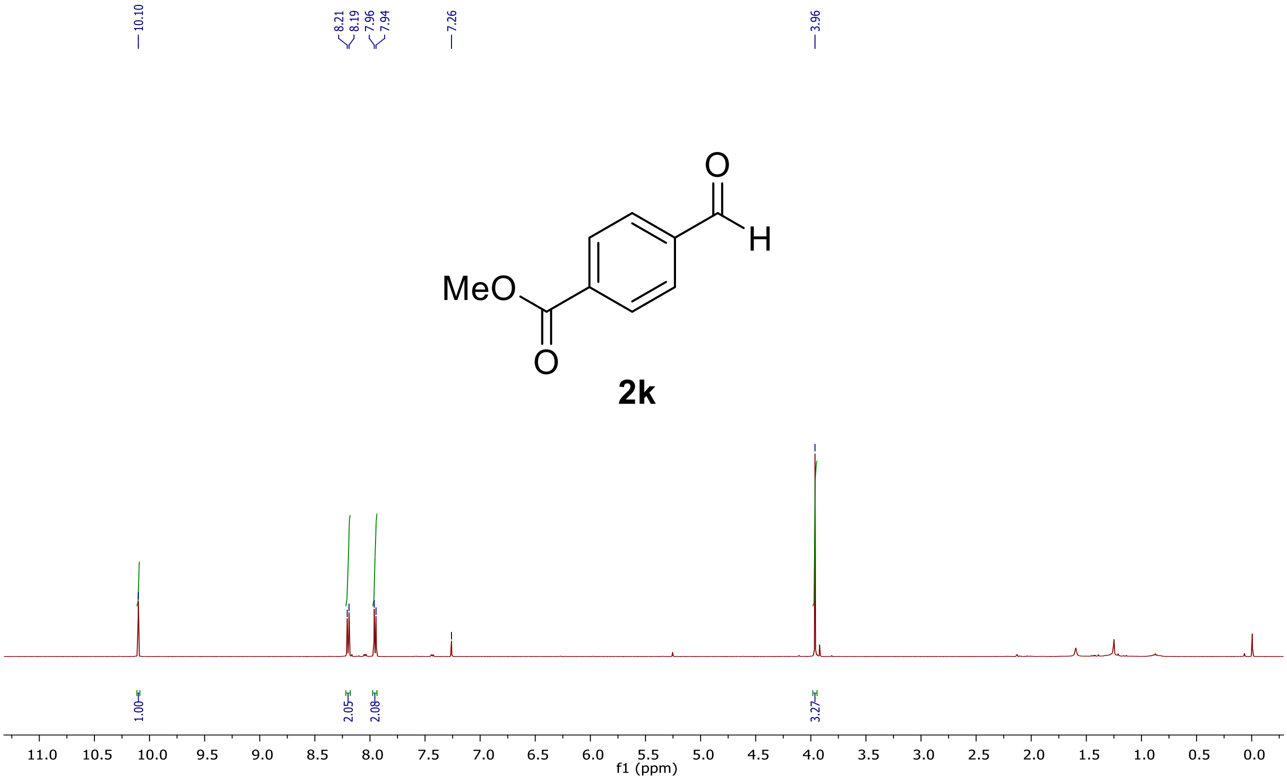
10.10
8.21
8.19
7.96
7.94
7.26
3.96
MeO
O
H
O
2k
1.00
2.05
2.08
3.27
11.0 10.5 10.0 9.5 9.0 8.5 8.0 7.5 7.0 6.5 6.0 5.5 5.0 4.5 4.0 3.5 3.0 2.5 2.0 1.5 1.0 0.5 0.0
f1 (ppm)

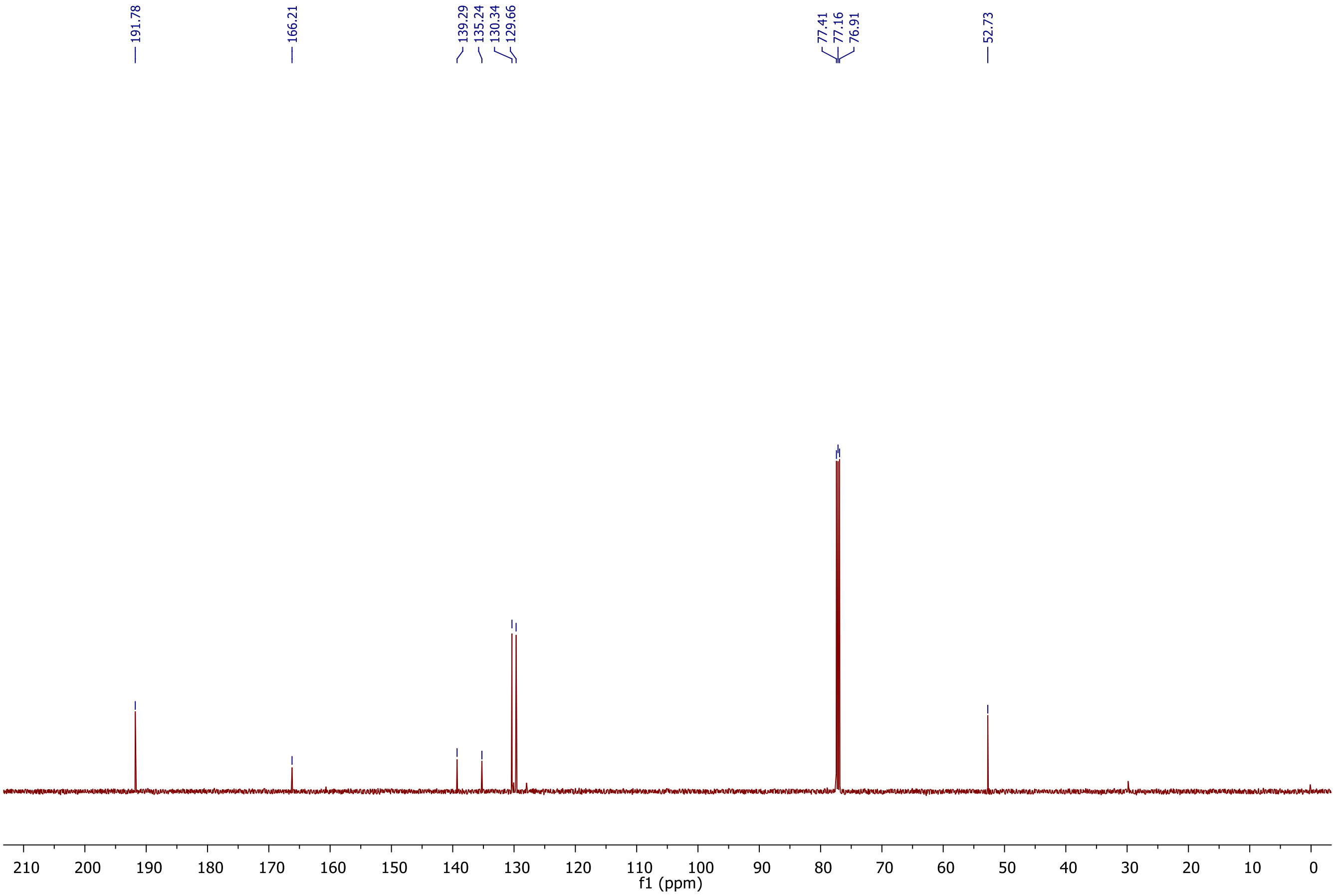
191.78
166.21
139.29
135.24
130.34
129.66
77.41
77.16
76.91
52.73
210 200 190 180 170 160 150 140 130 120 110 100 90 80 70 60 50 40 30 20 10 0
f1 (ppm)

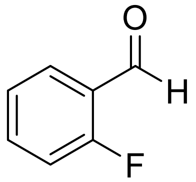

O
H
F

2l

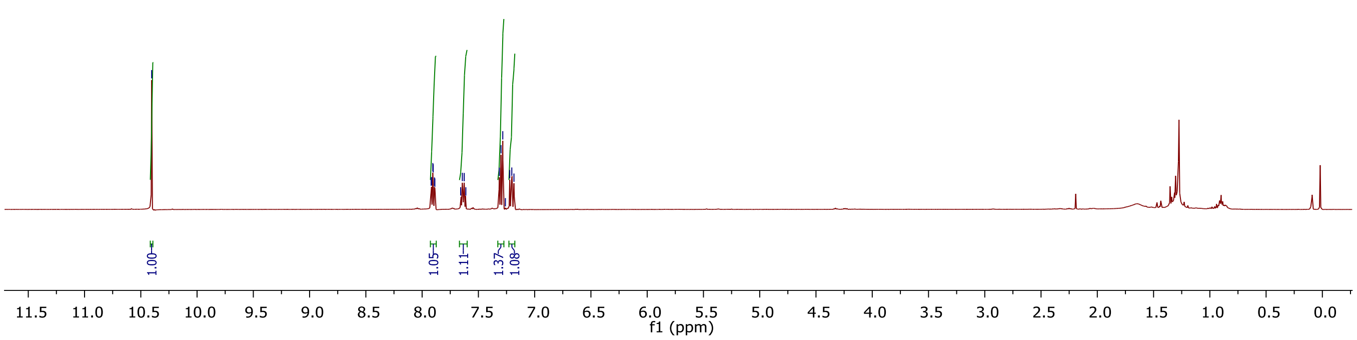

10.40
7.92
7.92
7.90
7.89
7.89
7.66
7.64
7.63
7.61
7.31
7.30
7.28
7.26
7.22
7.20
7.18
1.00
1.05
1.11
1.37
1.08
f1 (ppm)

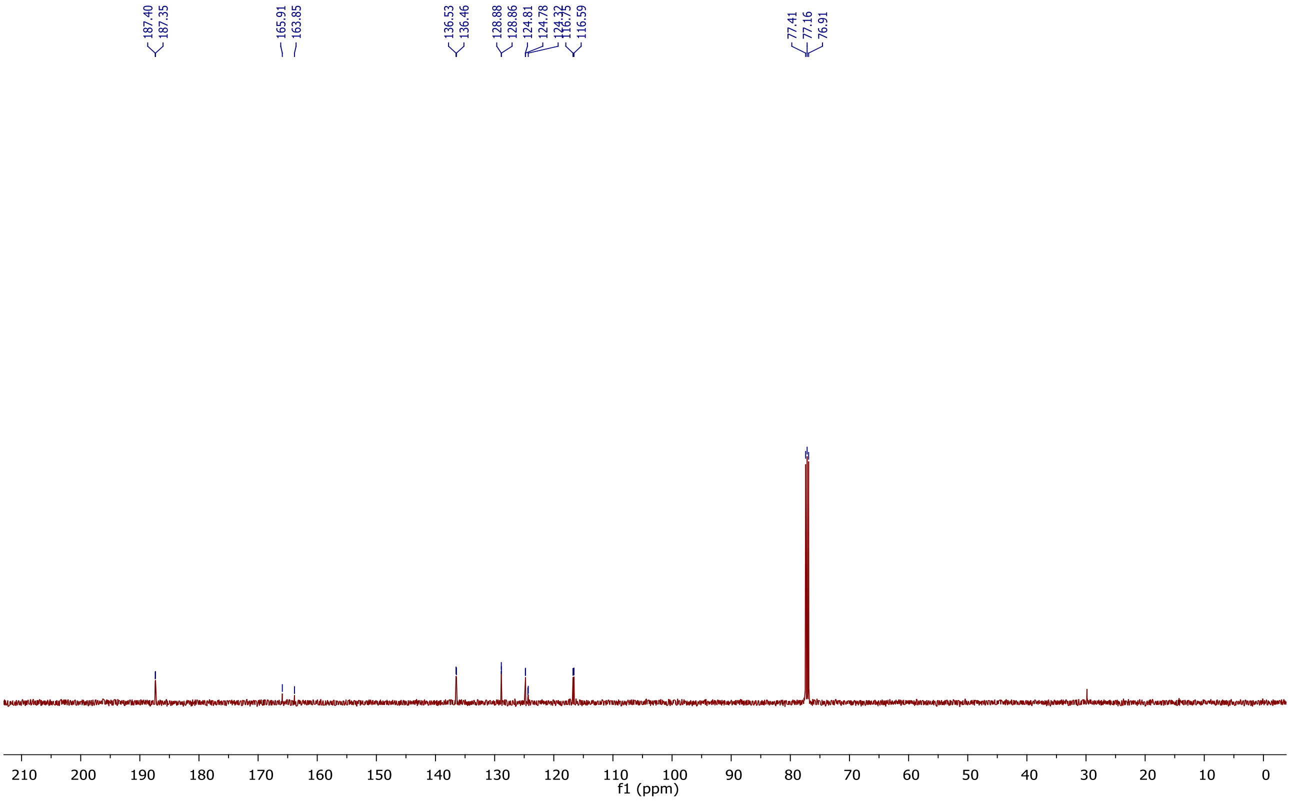

187.40
187.35
165.91
163.85
136.53
136.46
128.88
128.86
124.81
124.78
124.32
116.75
116.59
77.41
77.16
76.91
f1 (ppm)

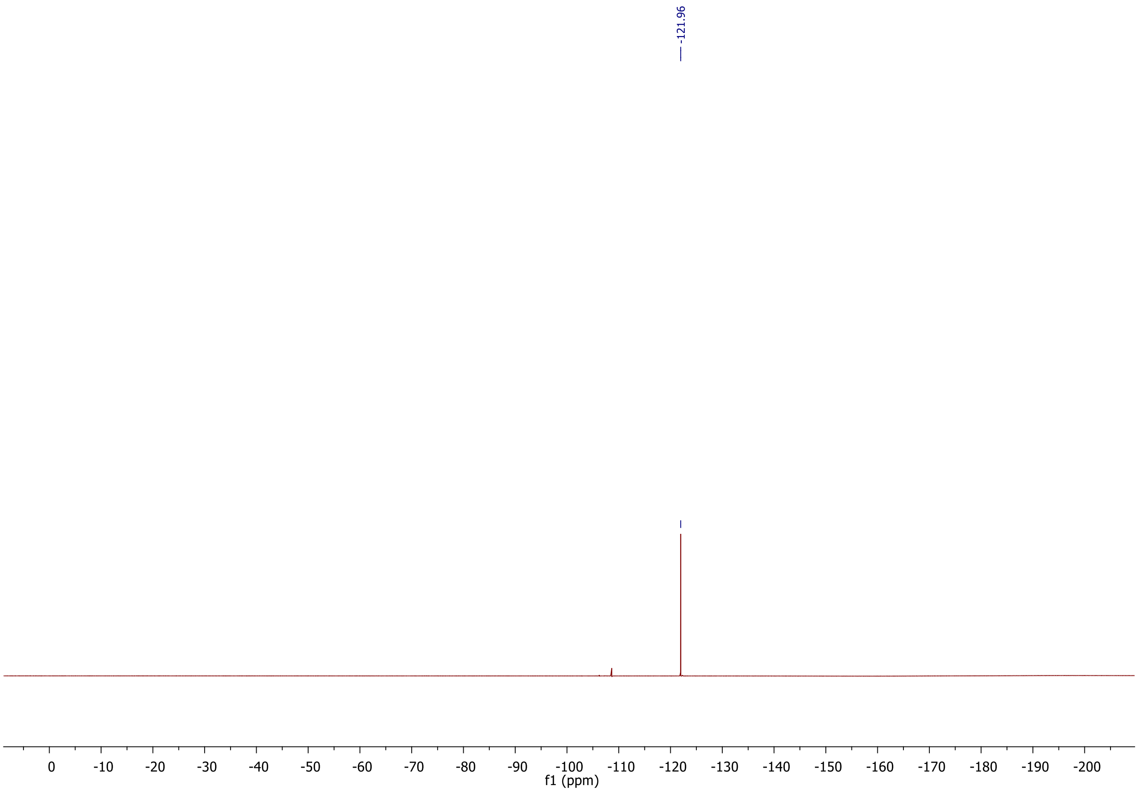
-121.96
0
-10
-20
-30
-40
-50
-60
-70
-80
-90
-100
-110
-120
-130
-140
-150
-160
-170
-180
-190
-200
f1 (ppm)

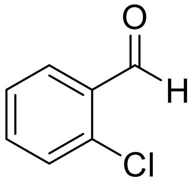

2m

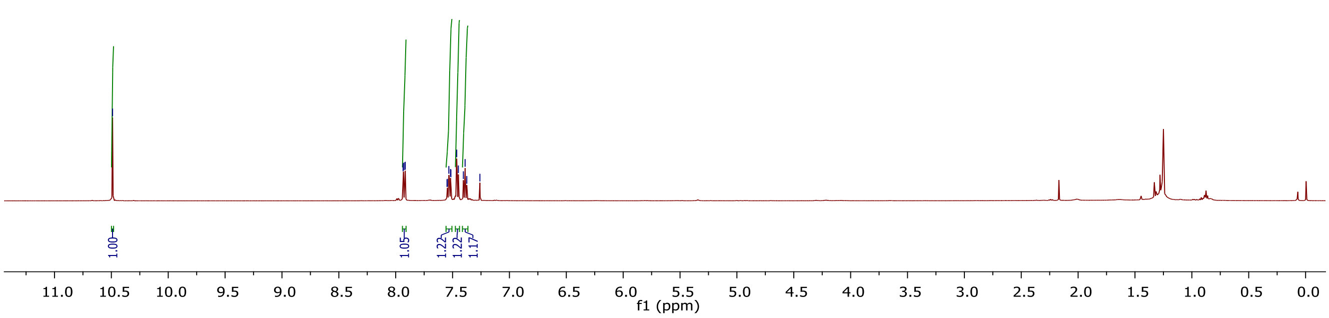
10.49
7.94
7.93
7.92
7.92
7.55
7.55
7.53
7.52
7.51
7.46
7.45
7.40
7.39
7.37
7.26
1.00
1.05
1.22
1.22
1.17
11.0 10.5 10.0 9.5 9.0 8.5 8.0 7.5 7.0 6.5 6.0 5.5 5.0 4.5 4.0 3.5 3.0 2.5 2.0 1.5 1.0 0.5 0.0
f1 (ppm)

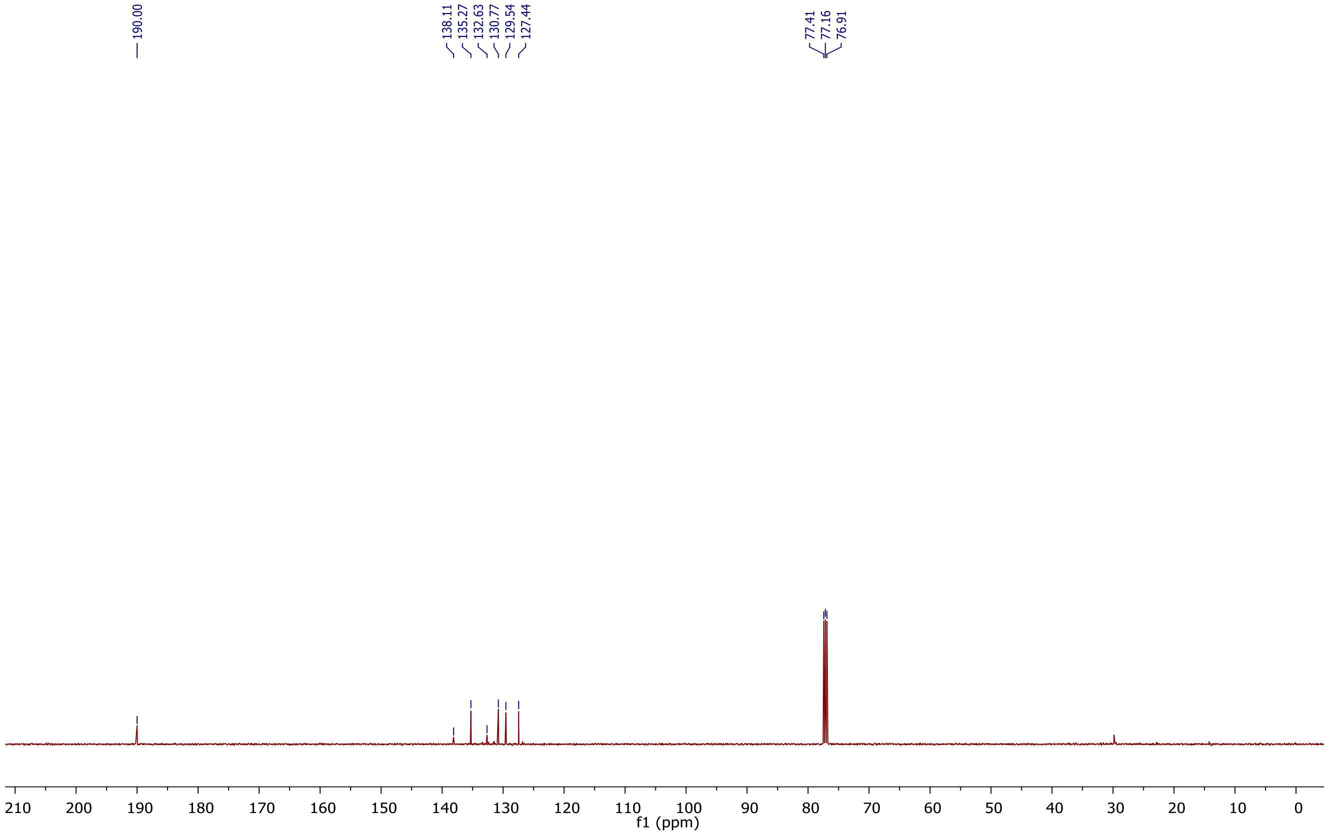
190.00
138.11
135.27
132.63
130.77
129.54
127.44
77.41
77.16
76.91
210 200 190 180 170 160 150 140 130 120 110 100 90 80 70 60 50 40 30 20 10 0
f1 (ppm)

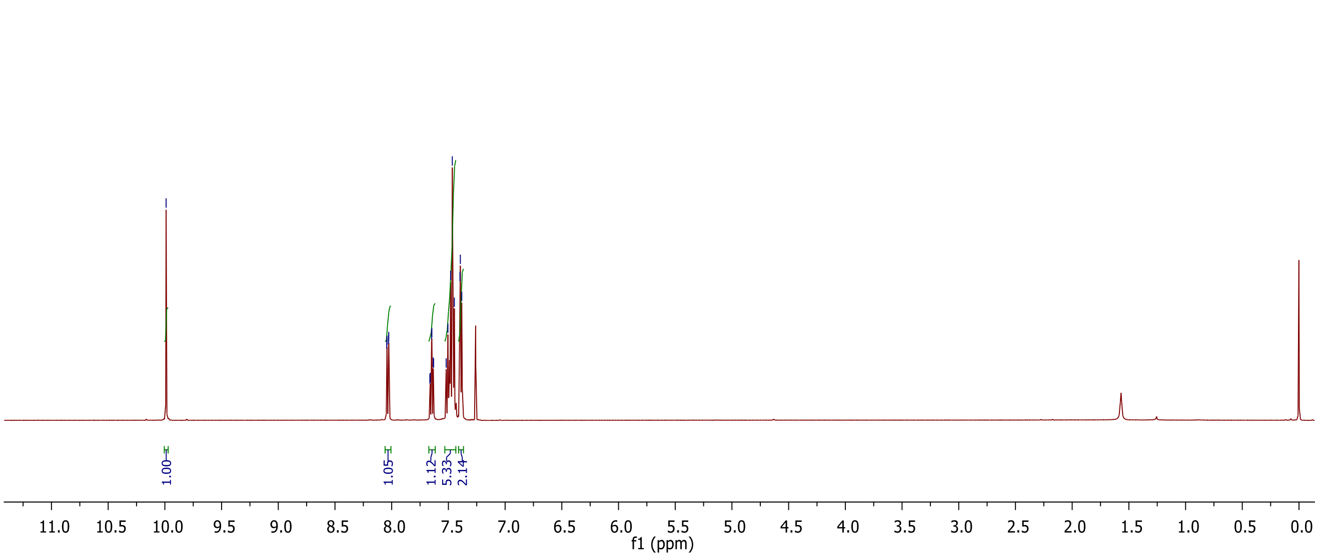
9.99
8.04
8.04
8.03
8.03
7.66
7.66
7.65
7.65
7.63
7.63
7.52
7.50
7.48
7.47
7.45
7.40
7.39
7.38
1.00
1.05
1.12
5.33
2.14
f1 (ppm)

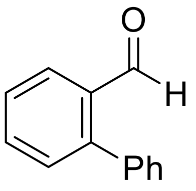
O
H
Ph

2n

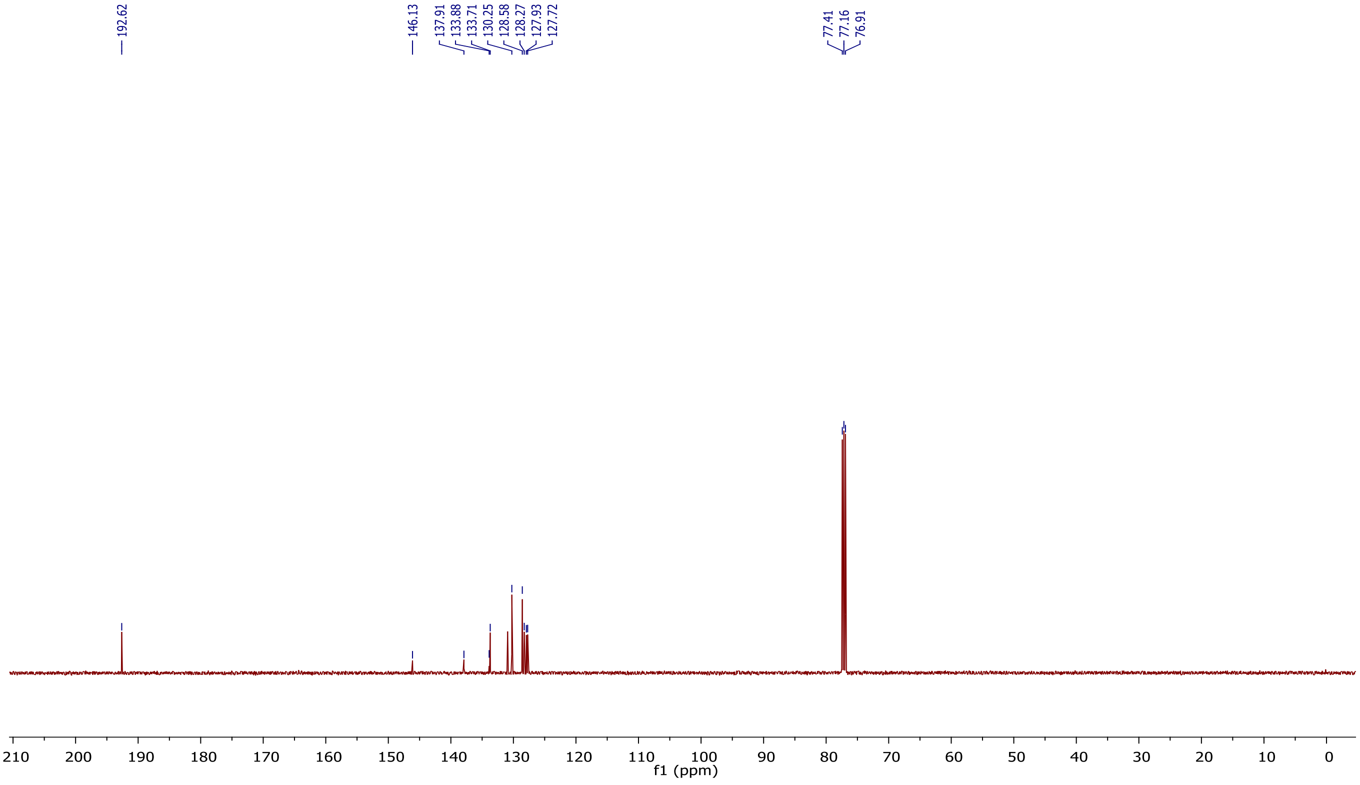
192.62
146.13
137.91
133.88
133.71
130.25
128.58
128.27
127.93
127.72
77.41
77.16
76.91
f1 (ppm)

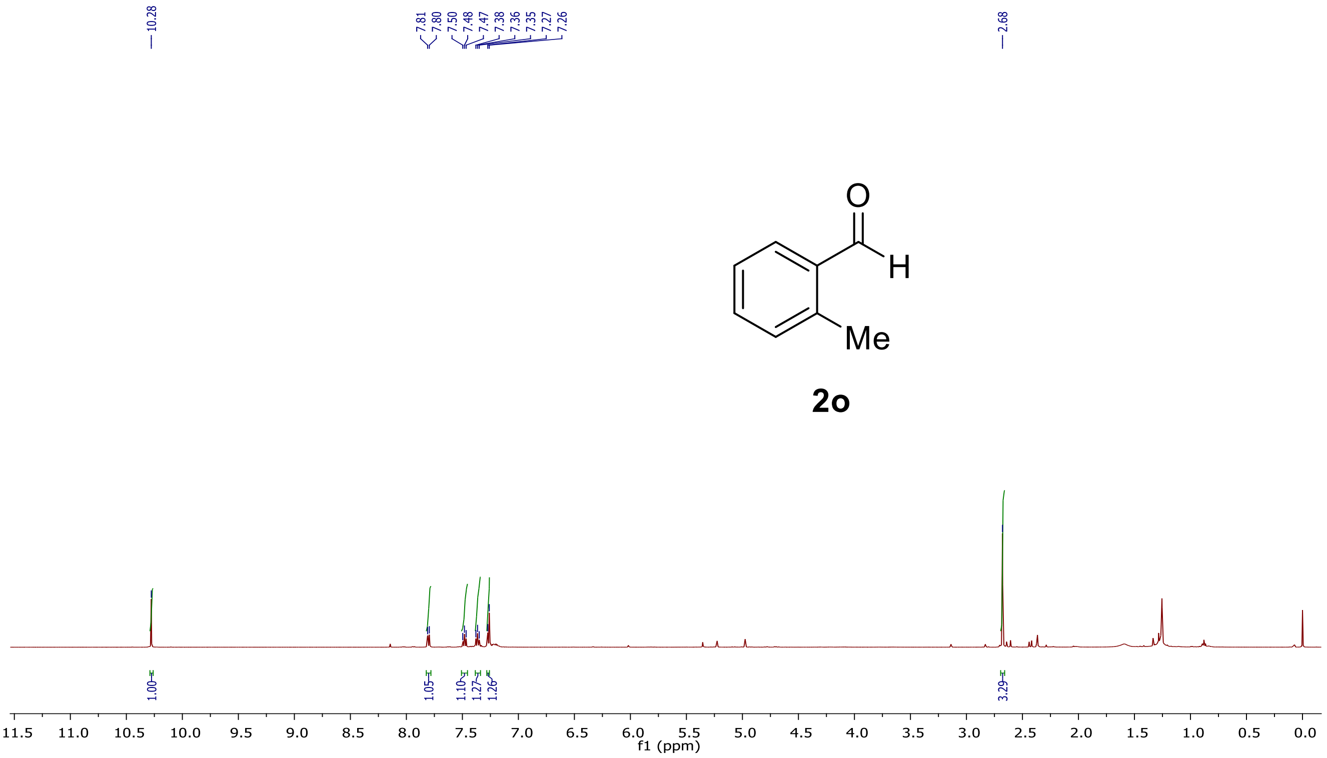
10.28
7.81
7.80
7.50
7.48
7.47
7.38
7.36
7.35
7.27
7.26
2.68
1.00
1.05
1.10
1.27
1.26
3.29
O
H
Me
f1 (ppm)

2o

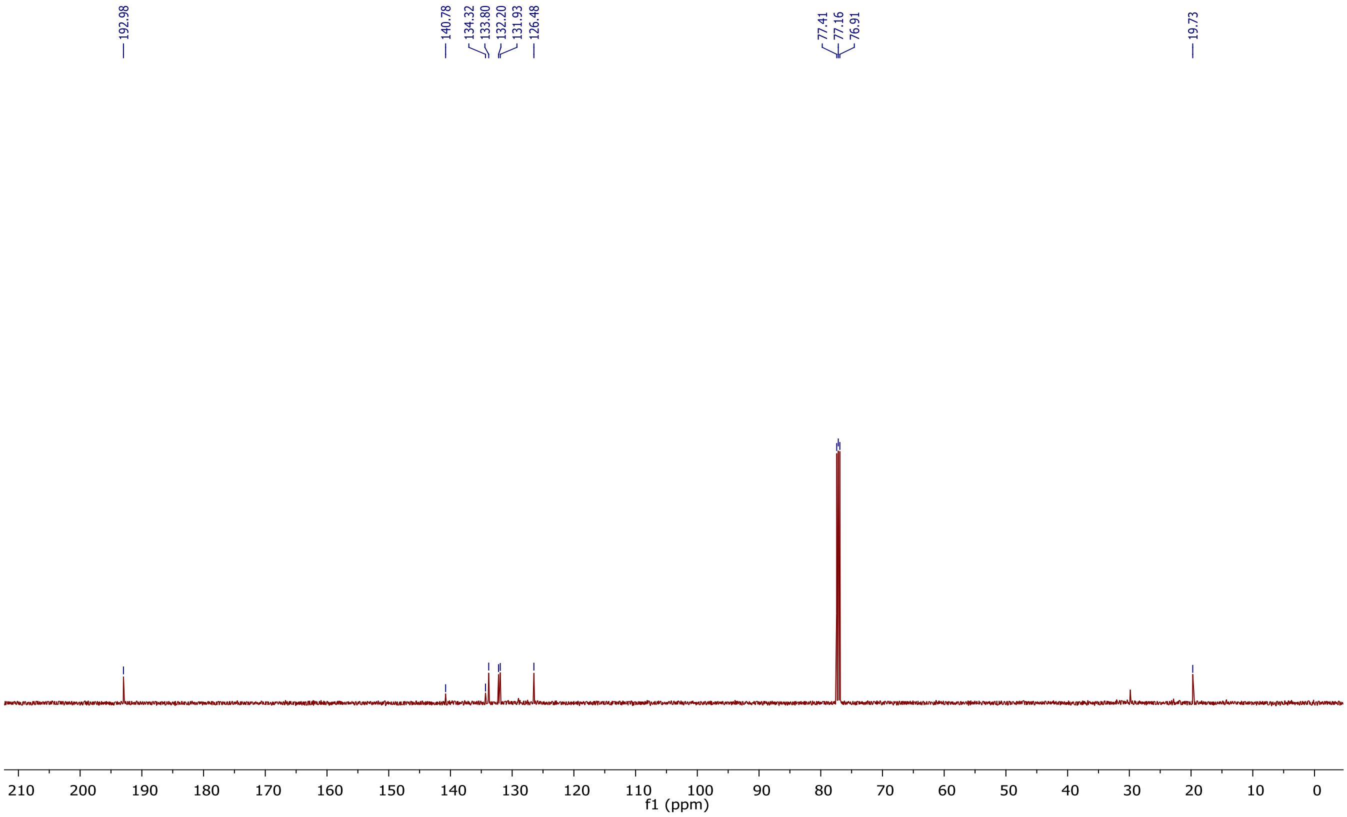
192.98
140.78
134.32
133.80
132.20
131.93
126.48
77.41
77.16
76.91
19.73
f1 (ppm)

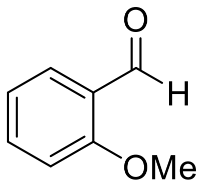

O
H
OMe

2p

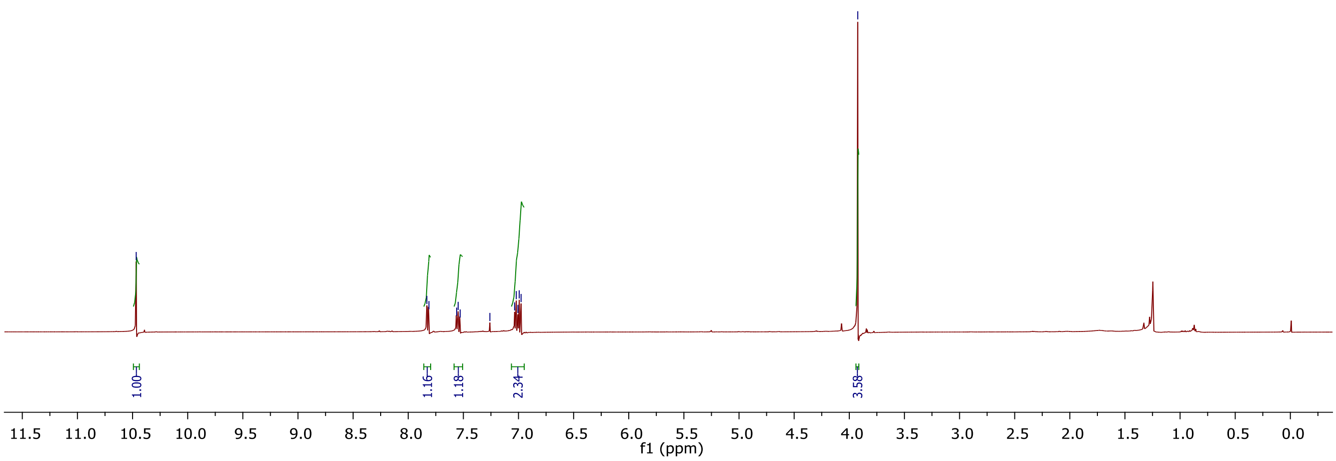

10.47
7.83
7.81
7.56
7.55
7.53
7.26
7.04
7.02
7.01
6.99
6.98
3.92
1.00
1.16
1.18
2.34
3.58
f1 (ppm)

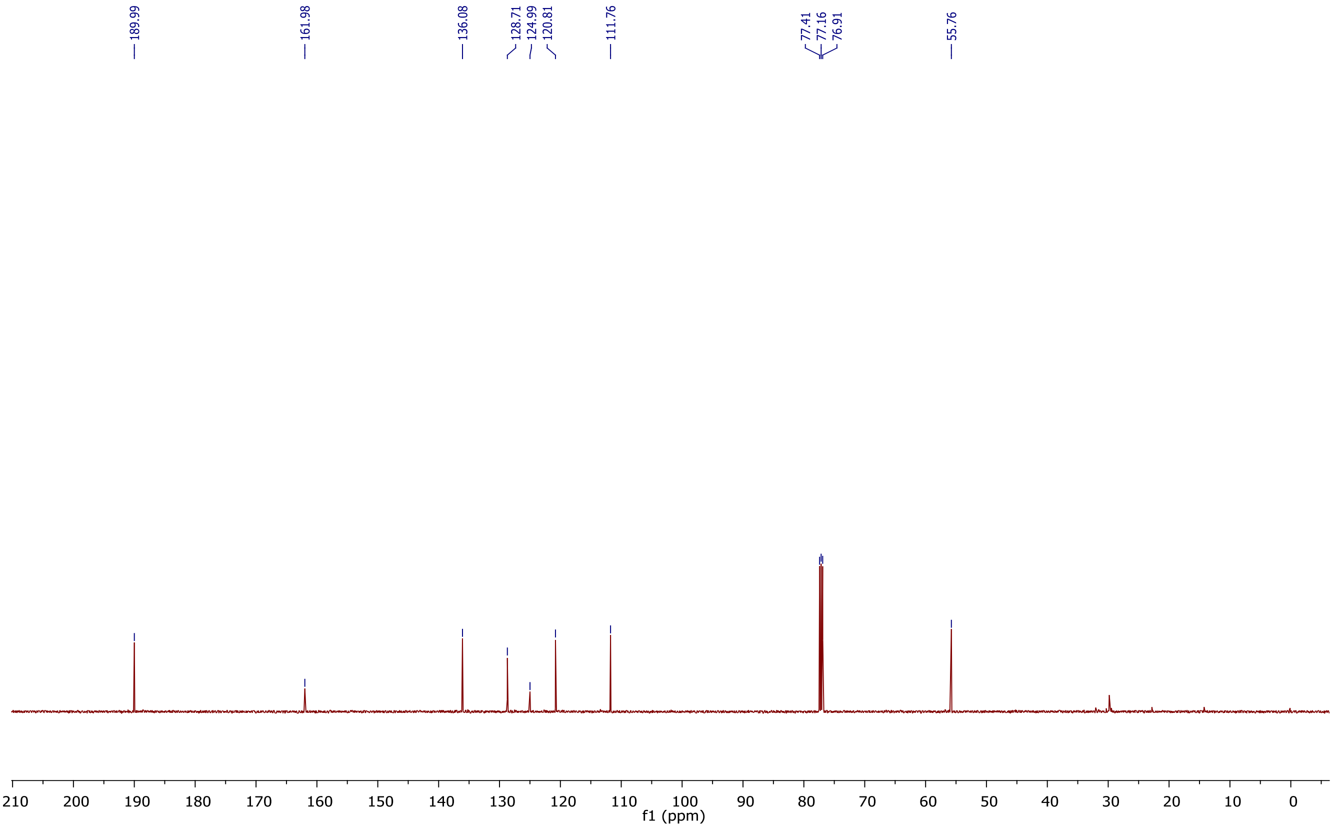

189.99
161.98
136.08
128.71
124.99
120.81
111.76
77.41
77.16
76.91
55.76
f1 (ppm)

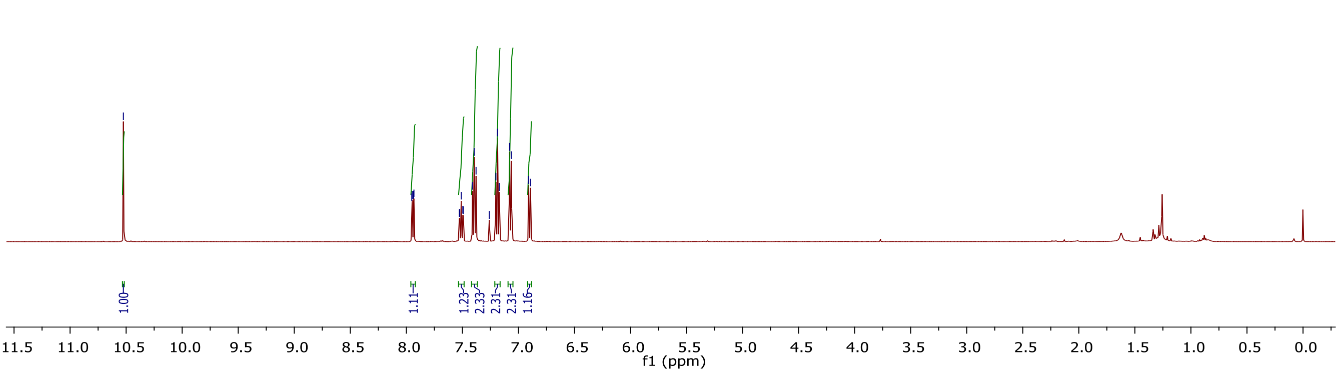
10.52
7.95
7.95
7.94
7.93
7.53
7.52
7.51
7.50
7.49
7.41
7.39
7.38
7.26
7.20
7.19
7.17
7.08
7.06
6.91
6.89
1.00
1.11
1.23
2.33
2.31
2.31
1.16
f1 (ppm)

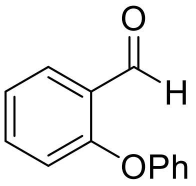
O
H
OPh

2q

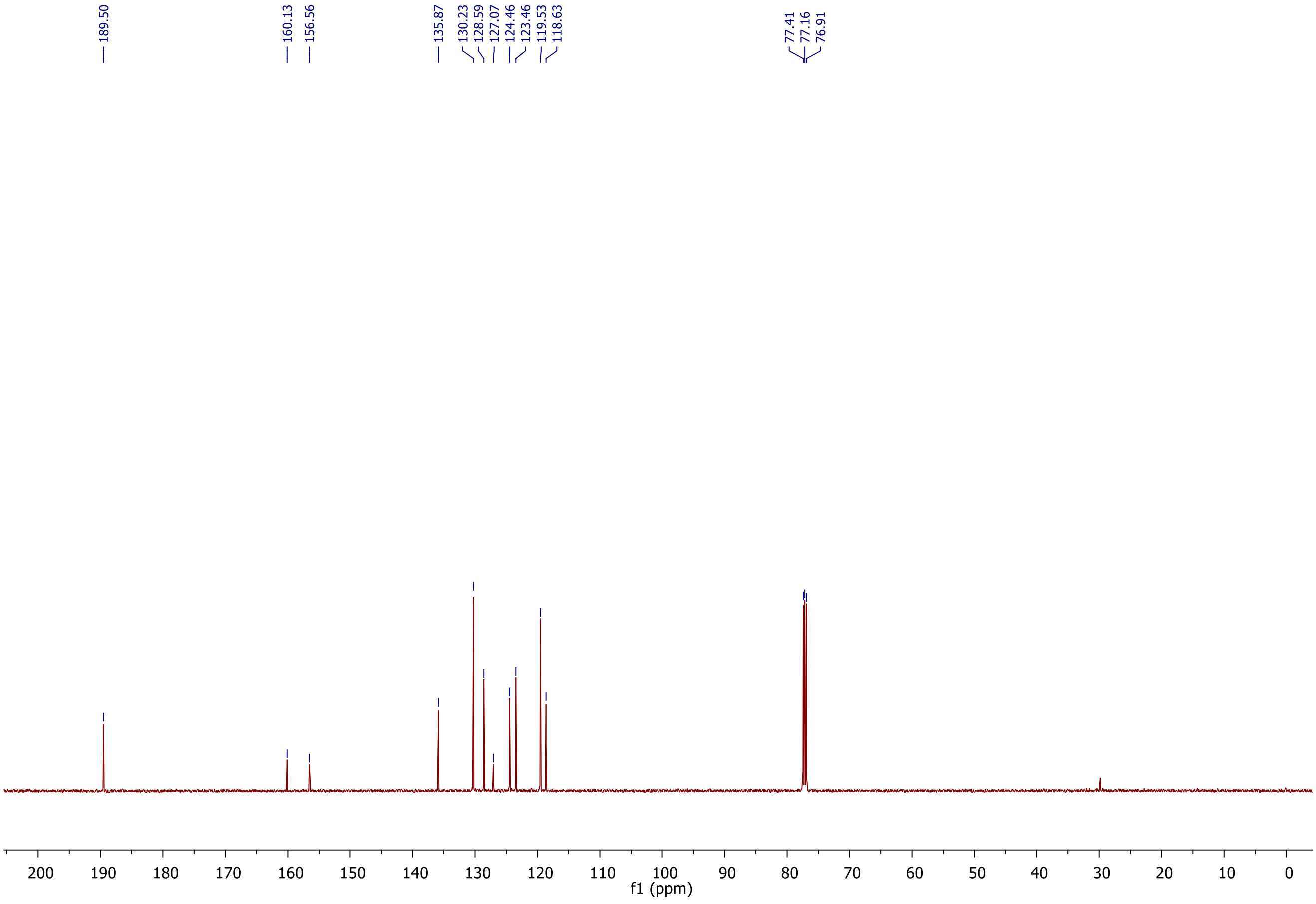
189.50
160.13
156.56
135.87
130.23
128.59
127.07
124.46
123.46
119.53
118.63
77.41
77.16
76.91
f1 (ppm)

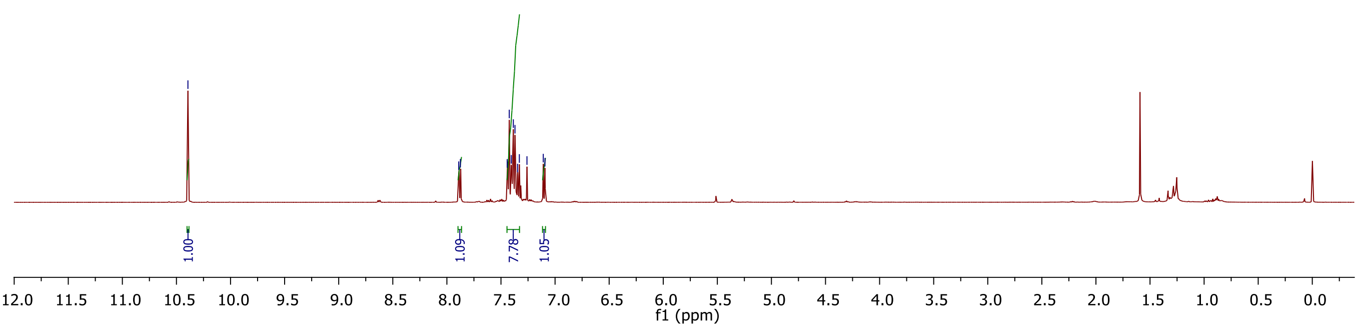
10.39
7.89
7.88
7.44
7.42
7.40
7.39
7.37
7.35
7.33
7.26
7.11
7.09
1.00
1.09
7.78
1.05
f1 (ppm)

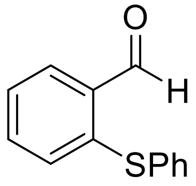
O
H
SPh

2r

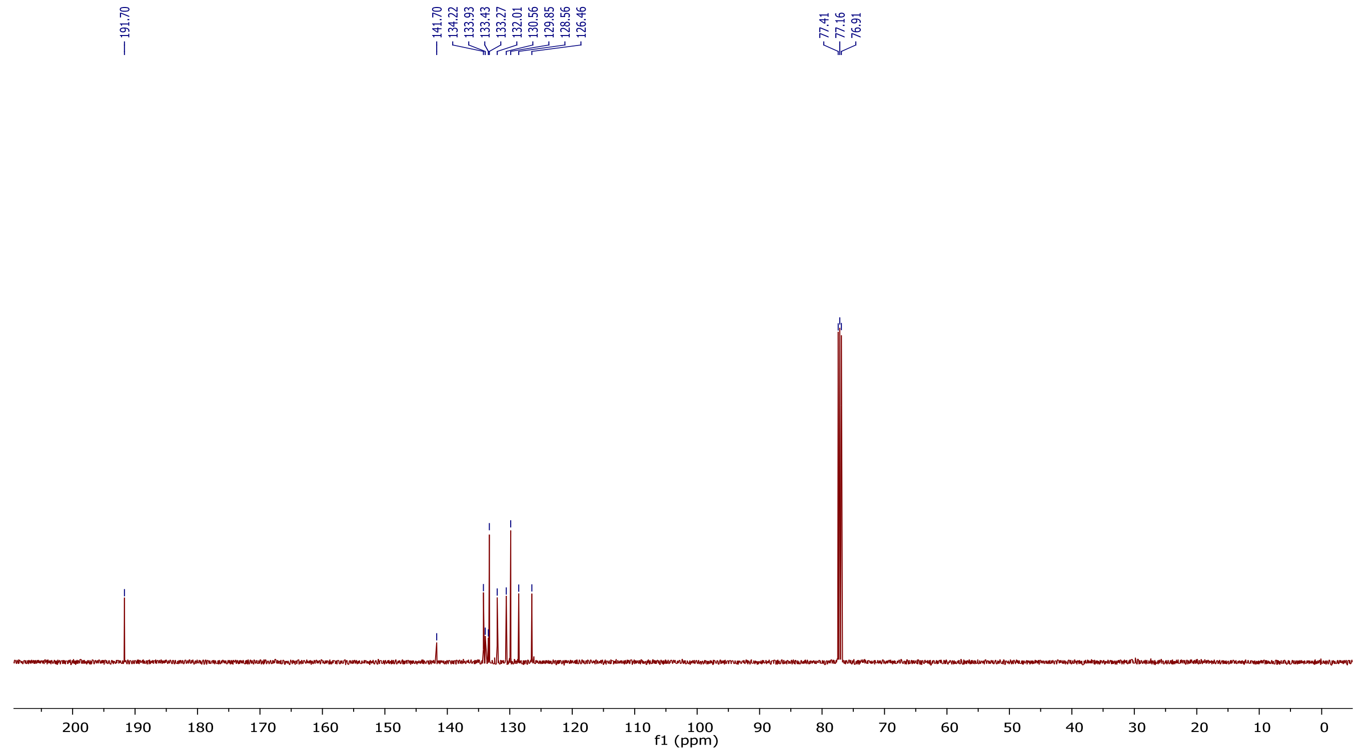
191.70
141.70
134.22
133.93
133.43
133.27
132.01
130.56
129.85
128.56
126.46
77.41
77.16
76.91
f1 (ppm)

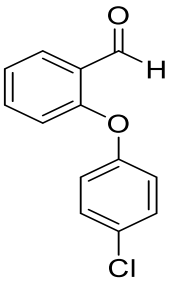

2s

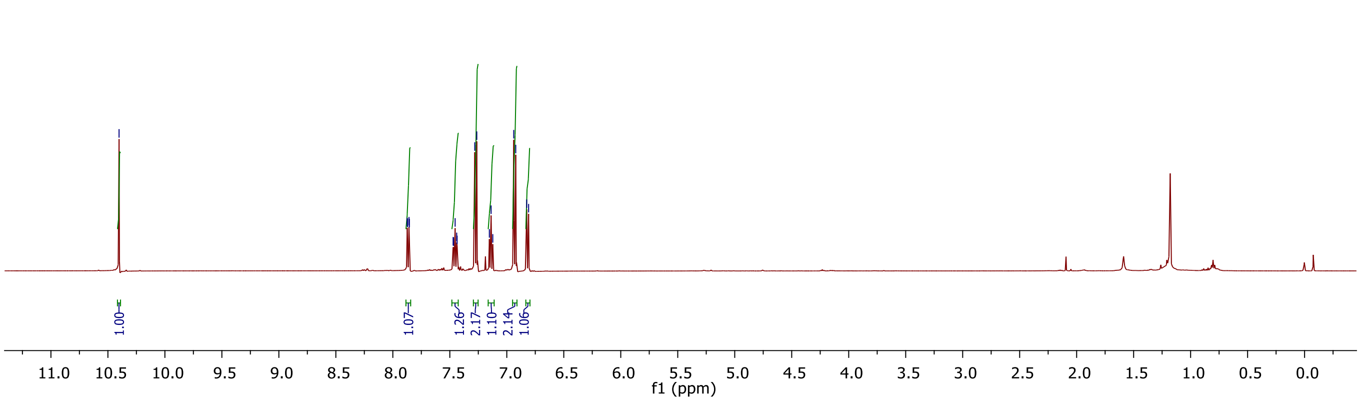

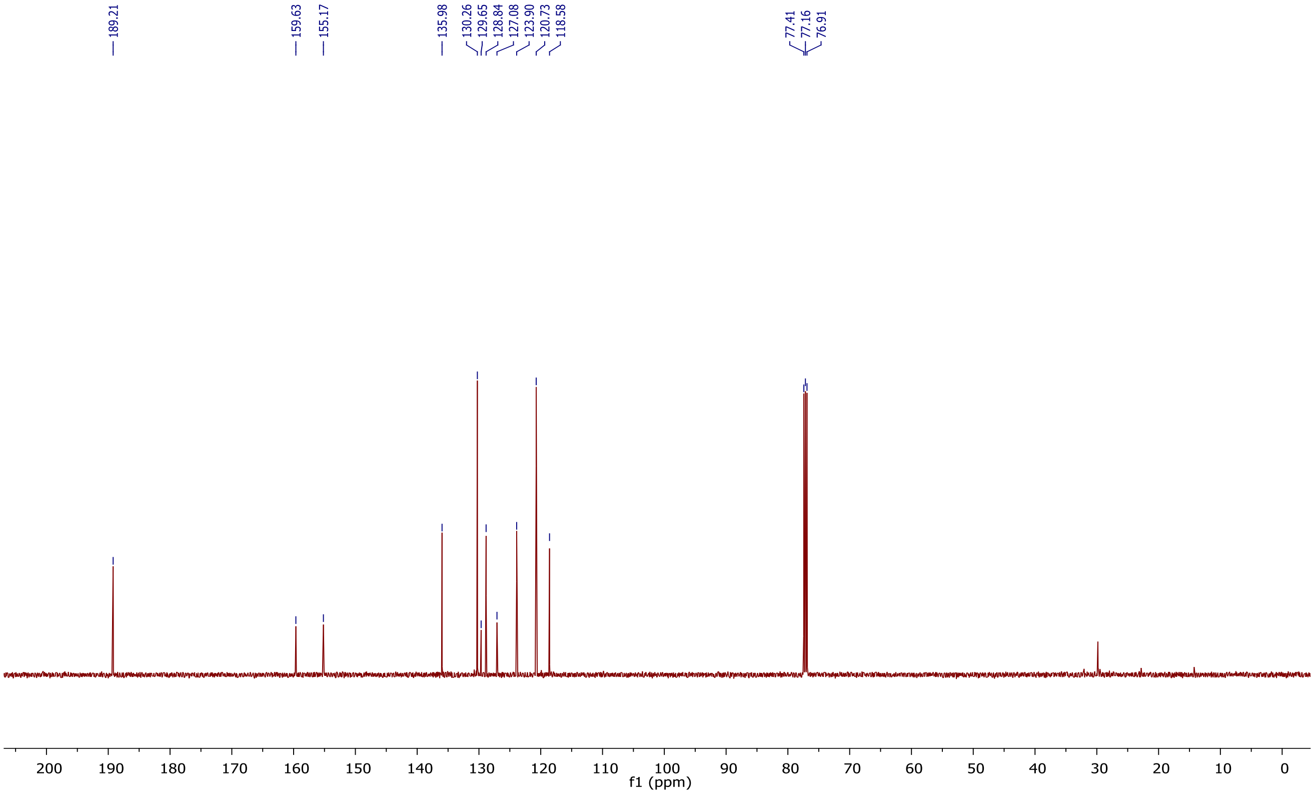

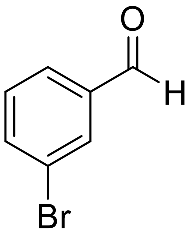
O
H
Br

2t

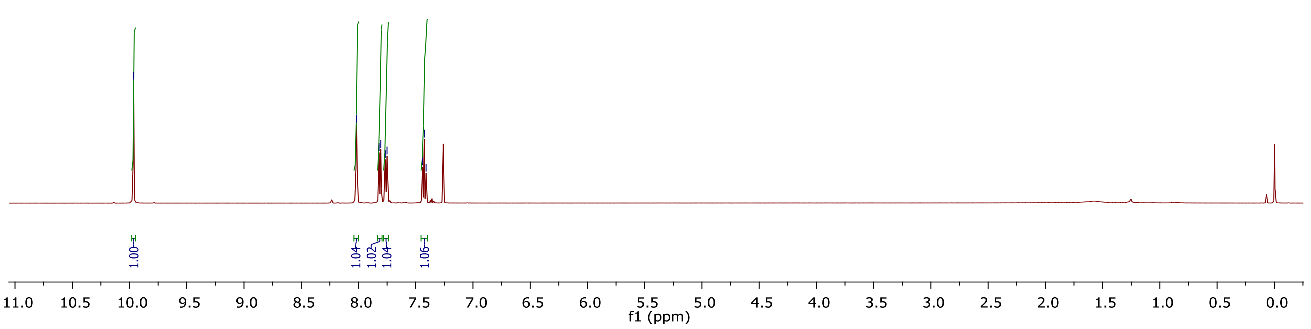
9.96
8.02
7.82
7.80
7.77
7.75
7.44
7.43
7.41
1.00
1.04
1.02
1.04
1.06
11.0 10.5 10.0 9.5 9.0 8.5 8.0 7.5 7.0 6.5 6.0 5.5 5.0 4.5 4.0 3.5 3.0 2.5 2.0 1.5 1.0 0.5 0.0
f1 (ppm)

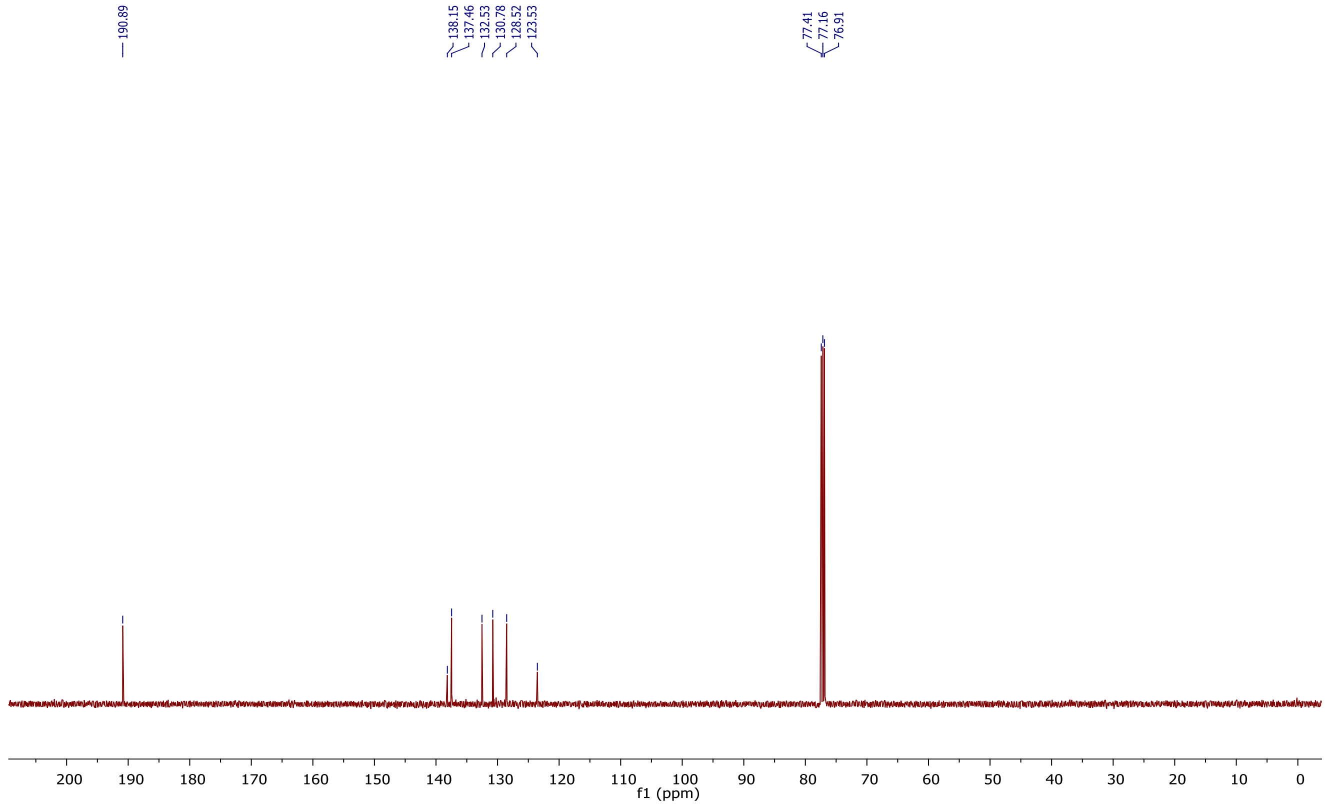
190.89
138.15
137.46
132.53
130.78
128.52
123.53
77.41
77.16
76.91
200 190 180 170 160 150 140 130 120 110 100 90 80 70 60 50 40 30 20 10 0
f1 (ppm)

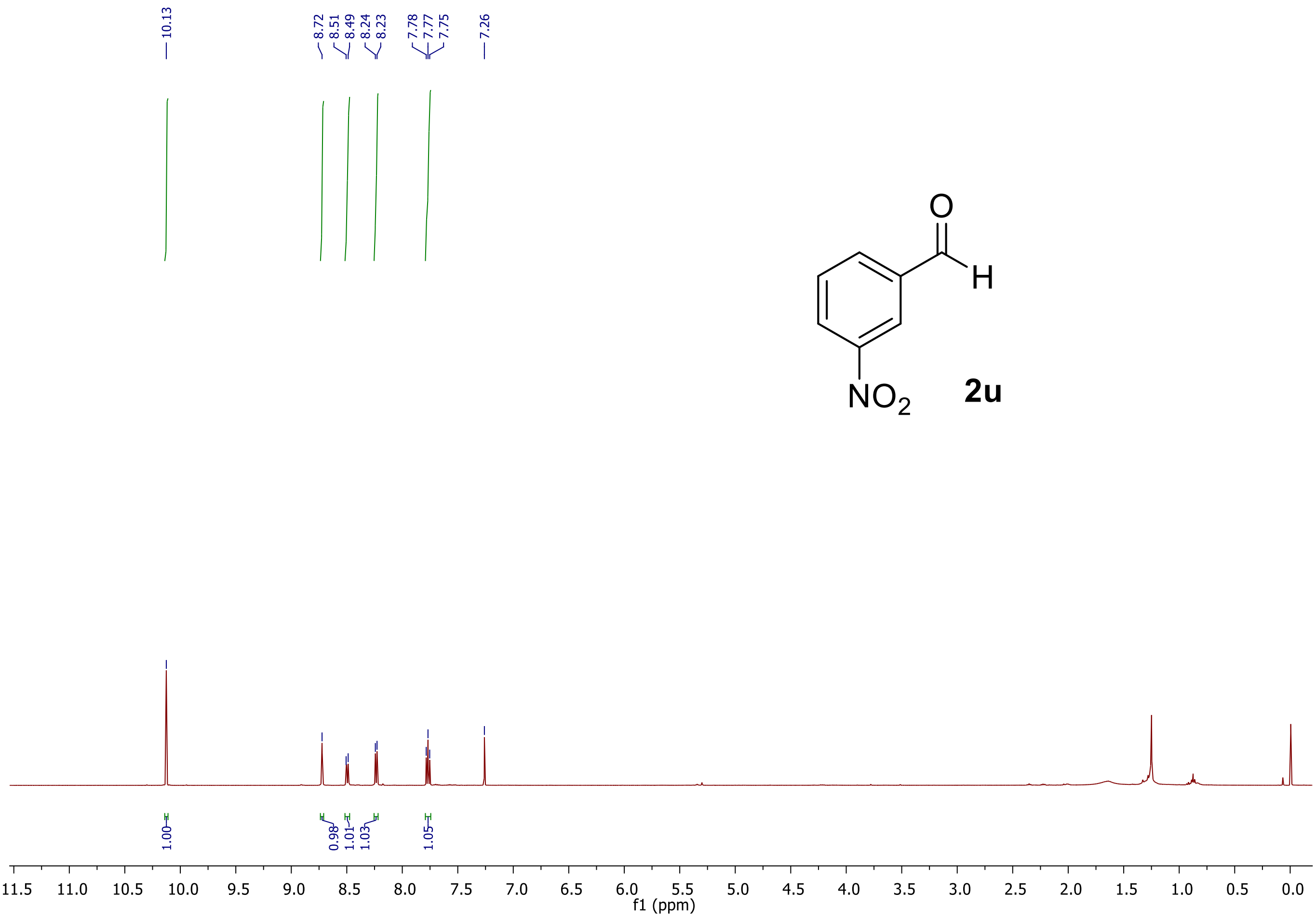
10.13
8.72
8.51
8.49
8.24
8.23
7.78
7.77
7.75
7.26
1.00
0.98
1.01
1.03
1.05
f1 (ppm)
O
H
NO_2

2u

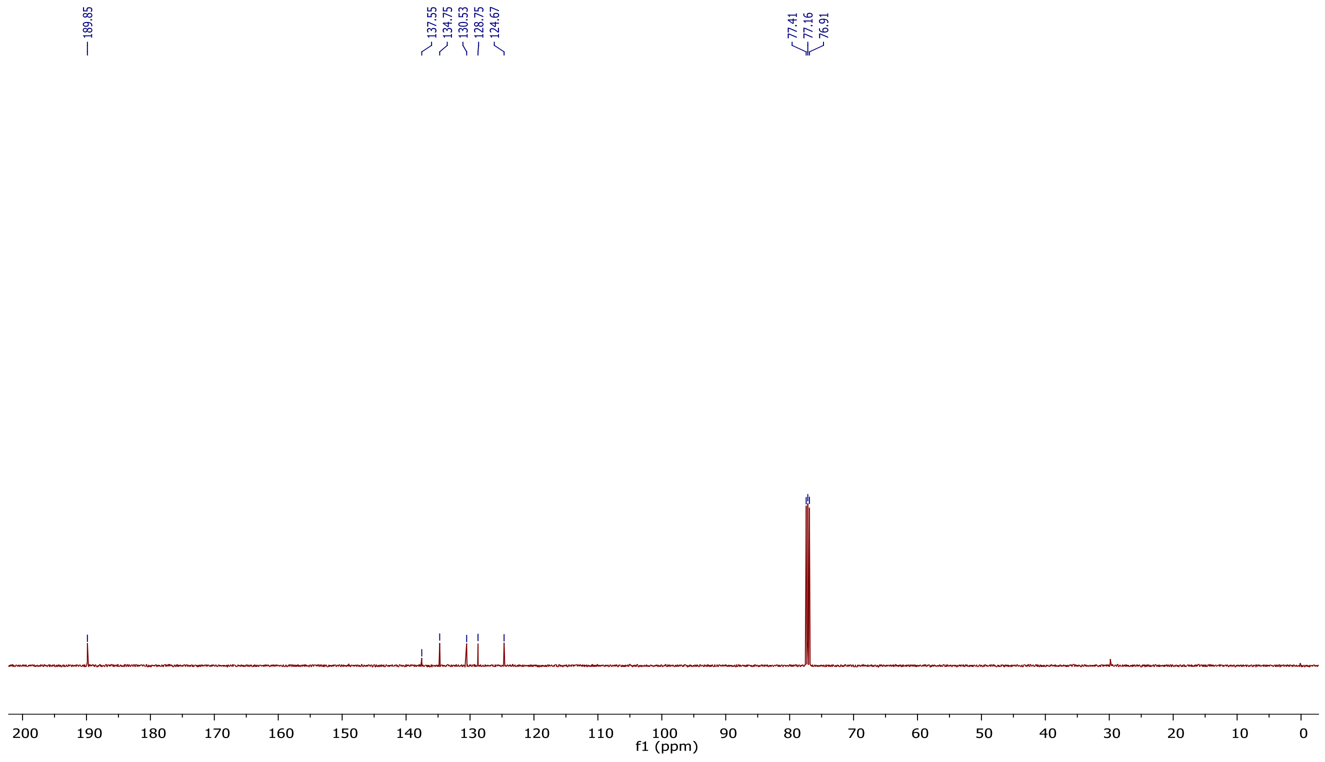
189.85
137.55
134.75
130.53
128.75
124.67
77.41
77.16
76.91
f1 (ppm)

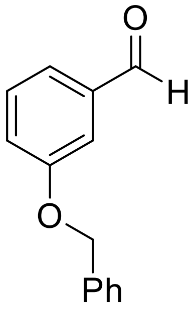

2v

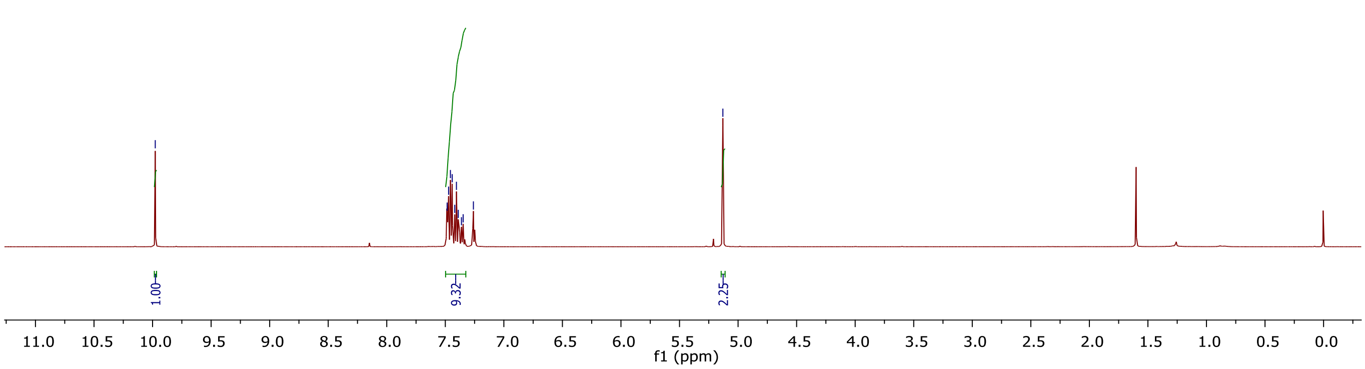

9.98
7.48
7.47
7.46
7.44
7.42
7.40
7.39
7.36
7.35
7.26
5.13
1.00
9.32
2.25
f1 (ppm)

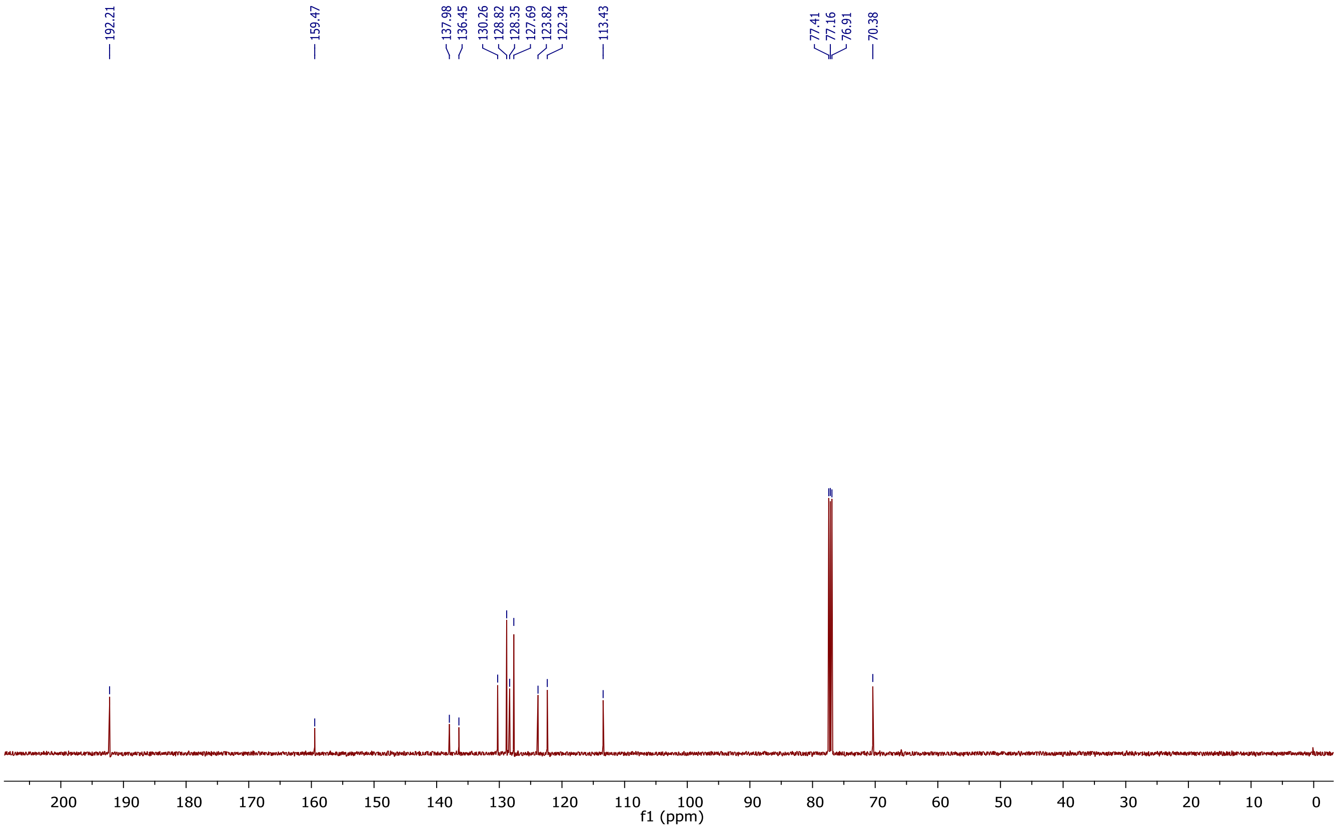

192.21
159.47
137.98
136.45
130.26
128.82
128.35
127.69
123.82
122.34
113.43
77.41
77.16
76.91
70.38
f1 (ppm)

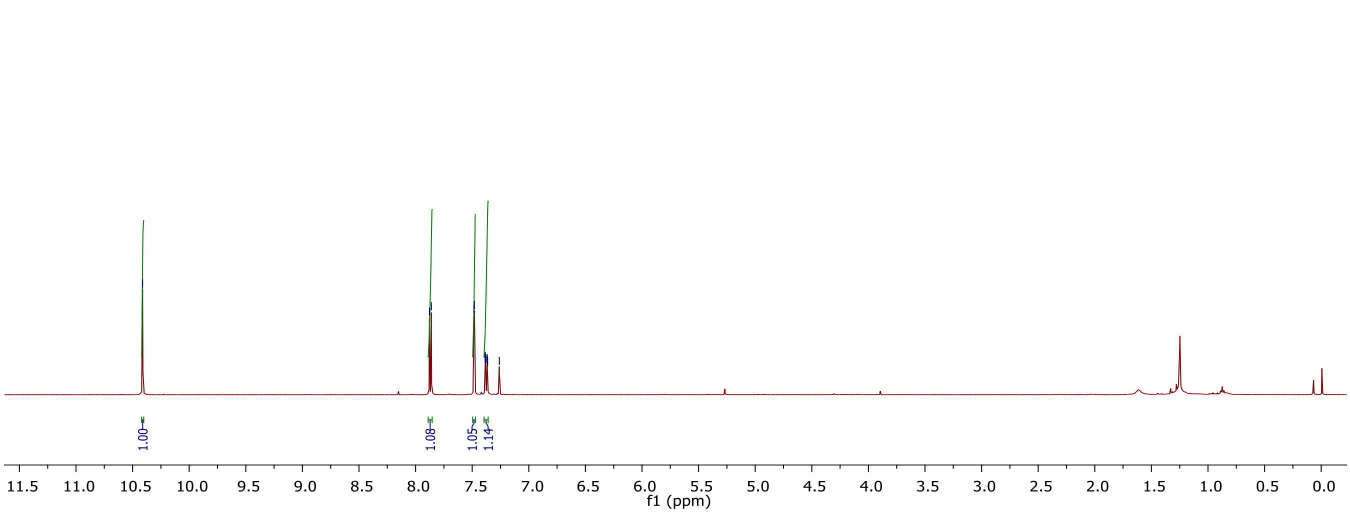

10.41
7.88
7.86
7.48
7.48
7.39
7.38
7.38
7.38
7.37
7.37
7.37
7.36
7.26
1.00
1.08
1.05
1.14
f1 (ppm)

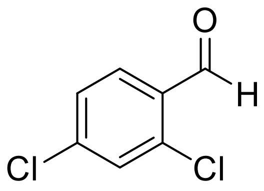

O
H
Cl
Cl

2w

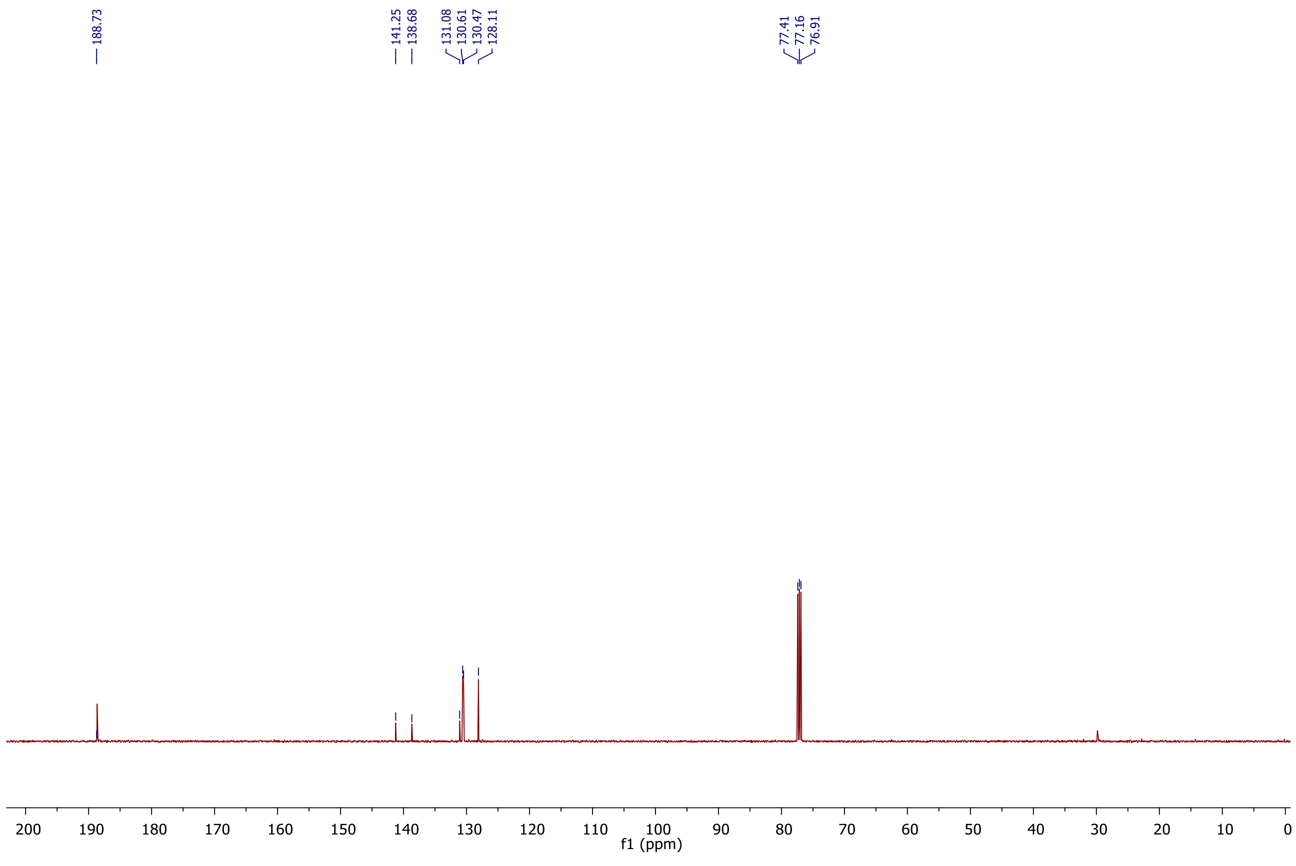

188.73
141.25
138.68
131.08
130.61
130.47
128.11
77.41
77.16
76.91
f1 (ppm)

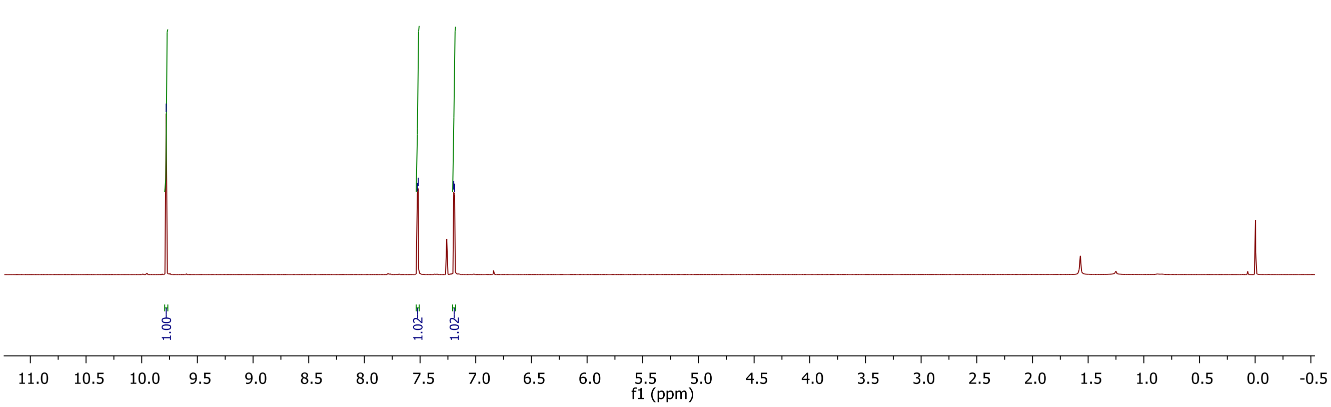
9.78
7.52
7.52
7.20
7.19
1.00
1.02
1.02
11.0 10.5 10.0 9.5 9.0 8.5 8.0 7.5 7.0 6.5 6.0 5.5 5.0 4.5 4.0 3.5 3.0 2.5 2.0 1.5 1.0 0.5 0.0 -0.5
f1 (ppm)

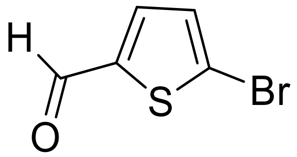
H
O
S
Br

2x

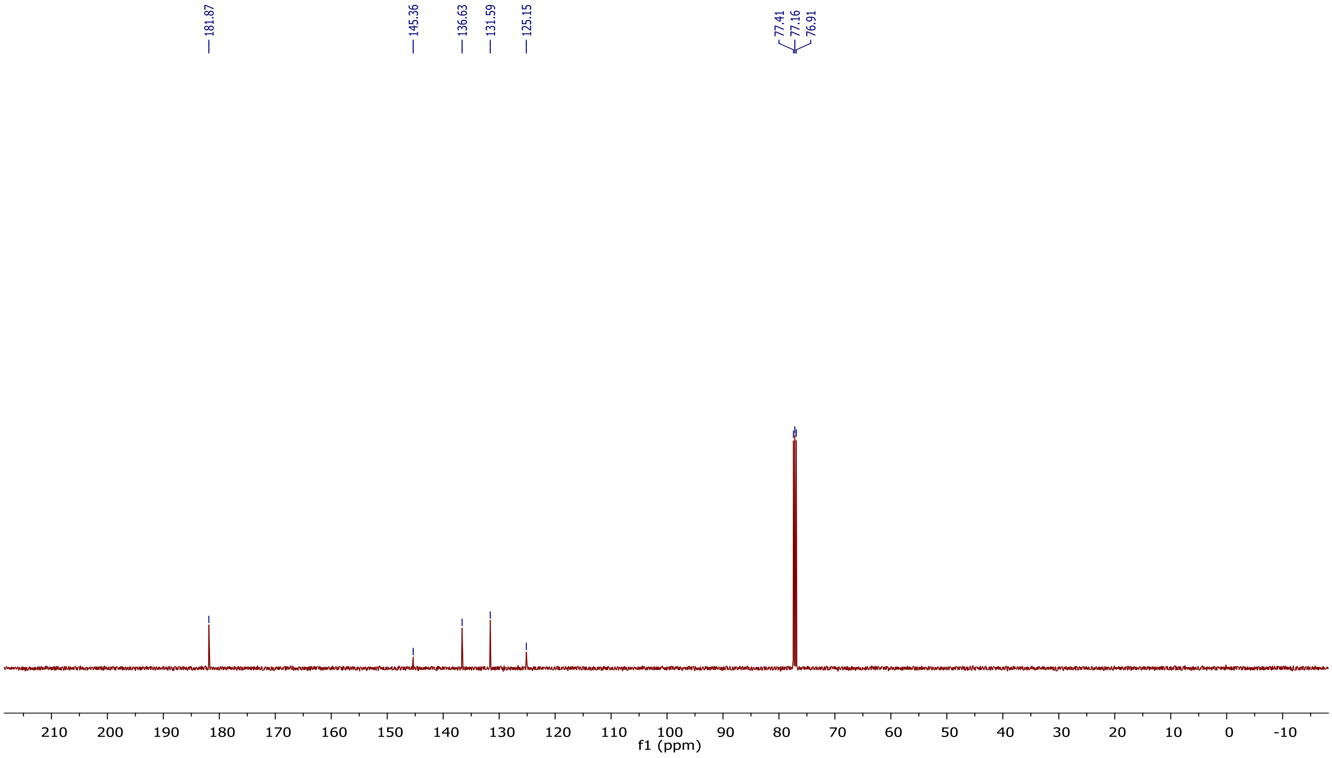
181.87
145.36
136.63
131.59
125.15
77.41
77.16
76.91
210 200 190 180 170 160 150 140 130 120 110 100 90 80 70 60 50 40 30 20 10 0 -10
f1 (ppm)

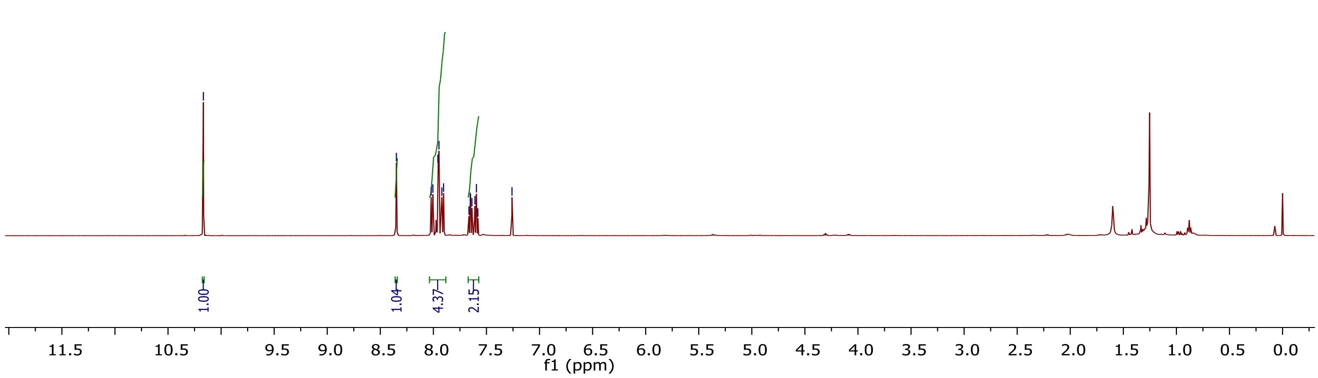
10.17
8.35
8.02
8.00
7.96
7.95
7.92
7.90
7.66
7.65
7.64
7.61
7.59
7.58
7.26
1.00
1.04
4.37
2.15
f1 (ppm)

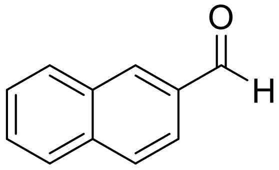
O
H

2y

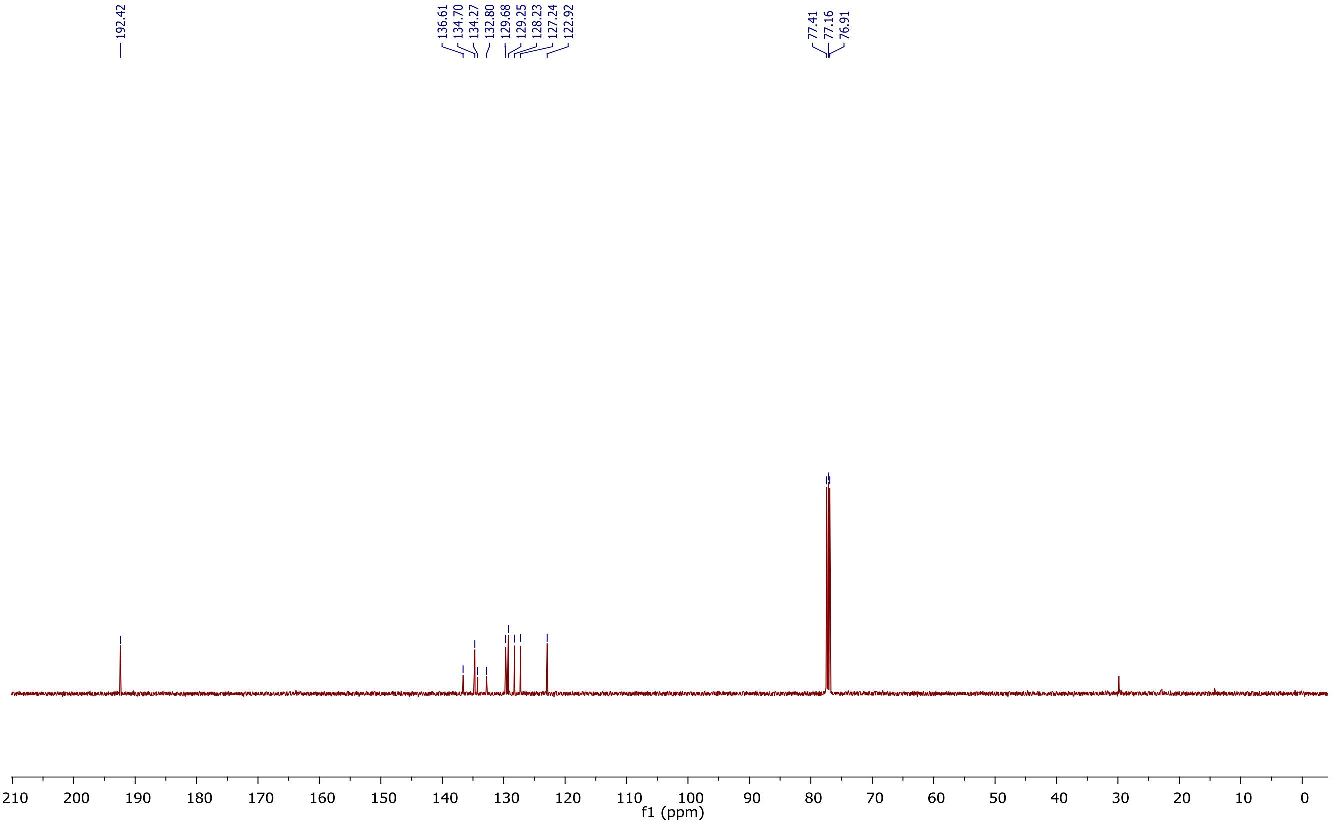
192.42
136.61
134.70
134.27
132.80
129.68
129.25
128.23
127.24
122.92
77.41
77.16
76.91
f1 (ppm)

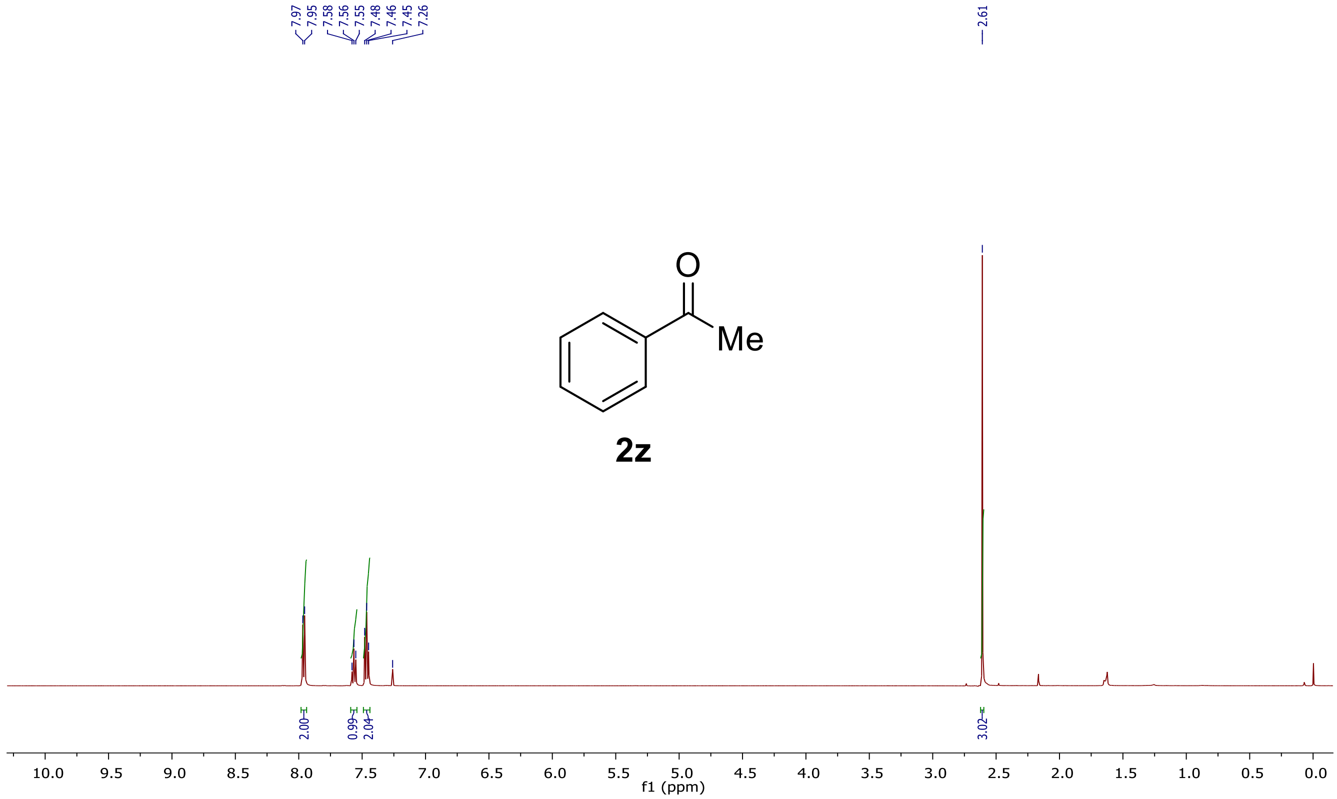
7.97
7.95
7.58
7.56
7.55
7.48
7.46
7.45
7.26
2.61
O
Me
2z
2.00
0.99
2.04
3.02
10.0 9.5 9.0 8.5 8.0 7.5 7.0 6.5 6.0 5.5 5.0 4.5 4.0 3.5 3.0 2.5 2.0 1.5 1.0 0.5 0.0
f1 (ppm)

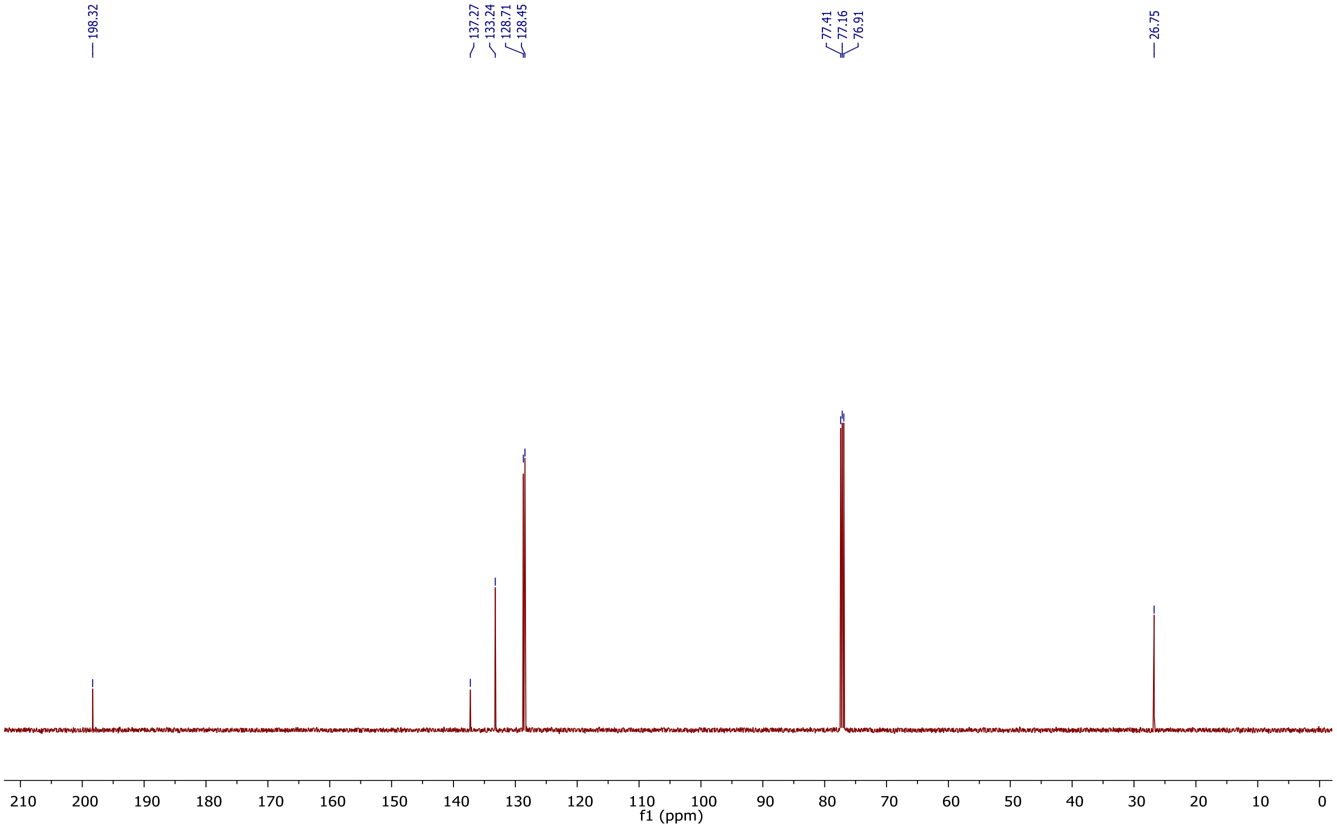
198.32
137.27
133.24
128.71
128.45
77.41
77.16
76.91
26.75
210 200 190 180 170 160 150 140 130 120 110 100 90 80 70 60 50 40 30 20 10 0
f1 (ppm)

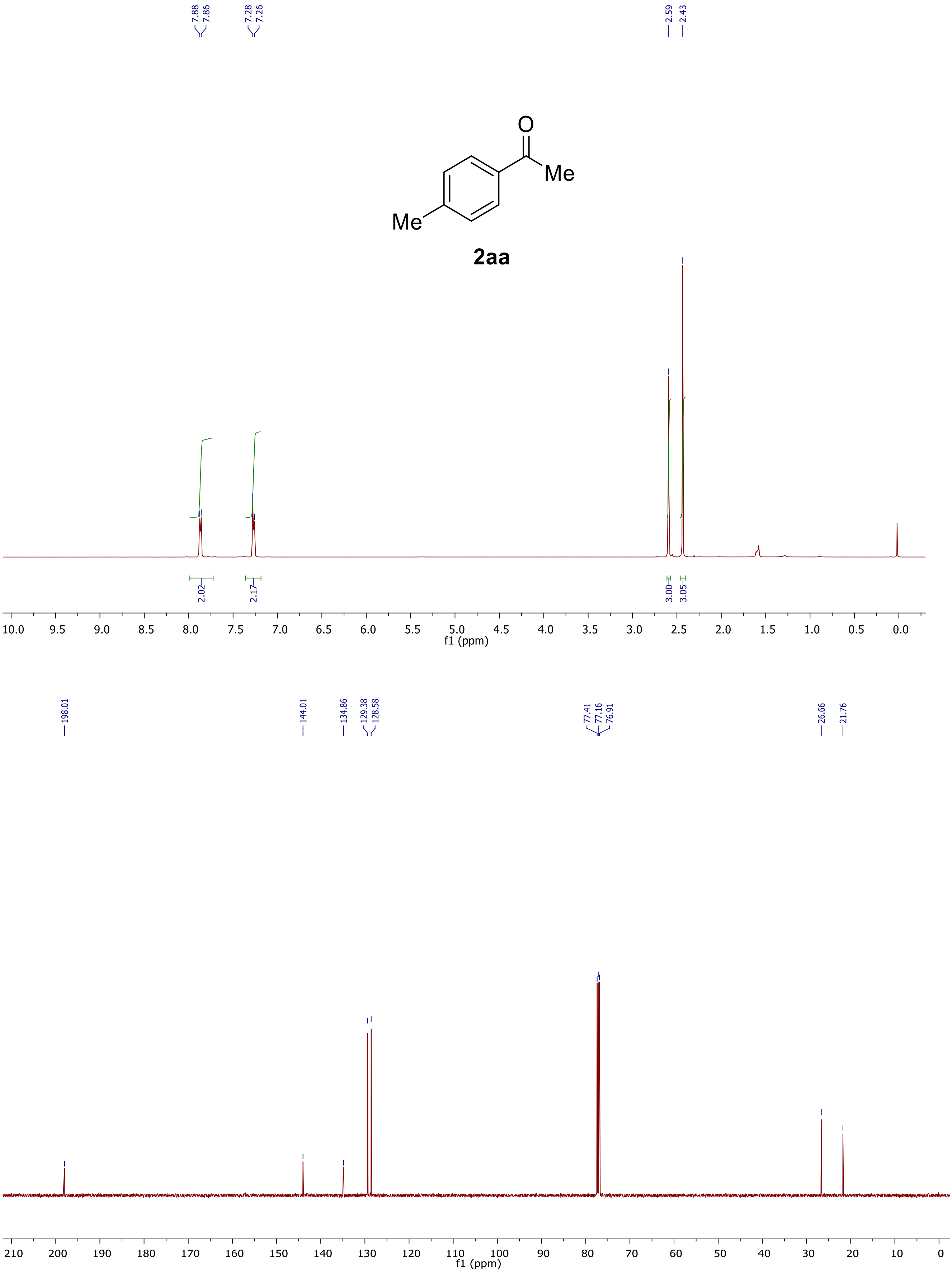
7.88
7.86
7.28
7.26
2.59
2.43
O
Me
Me
2aa
2.02
2.17
3.00
3.05
f1 (ppm)
198.01
144.01
134.86
129.38
128.58
77.41
77.16
76.91
26.66
21.76
f1 (ppm)

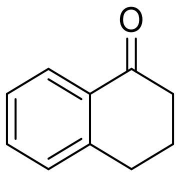
O

2ab

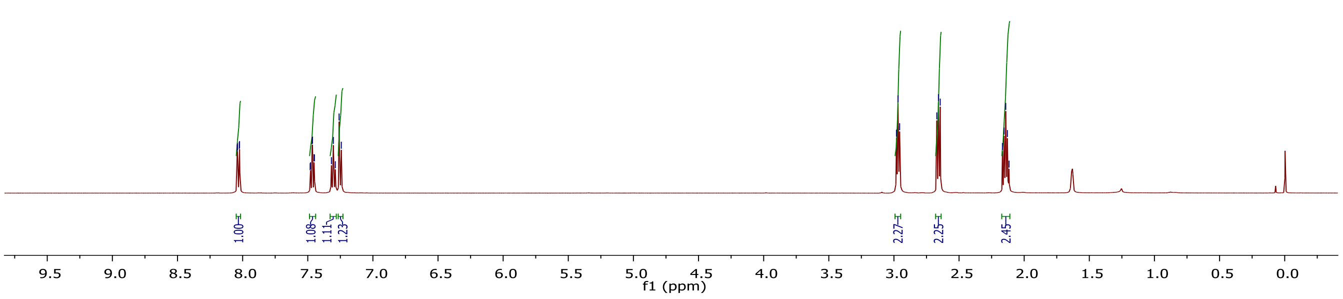
8.04 8.04 8.03 8.02 7.48 7.48 7.47 7.46 7.45 7.45 7.32 7.30 7.29 7.26 7.24
2.98 2.97 2.96 2.67 2.66 2.64 2.17 2.15 2.14 2.13 2.12
1.00 1.08 1.11 1.23 2.27 2.25 2.45
9.5 9.0 8.5 8.0 7.5 7.0 6.5 6.0 5.5 5.0 4.5 4.0 3.5 3.0 2.5 2.0 1.5 1.0 0.5 0.0
f1 (ppm)

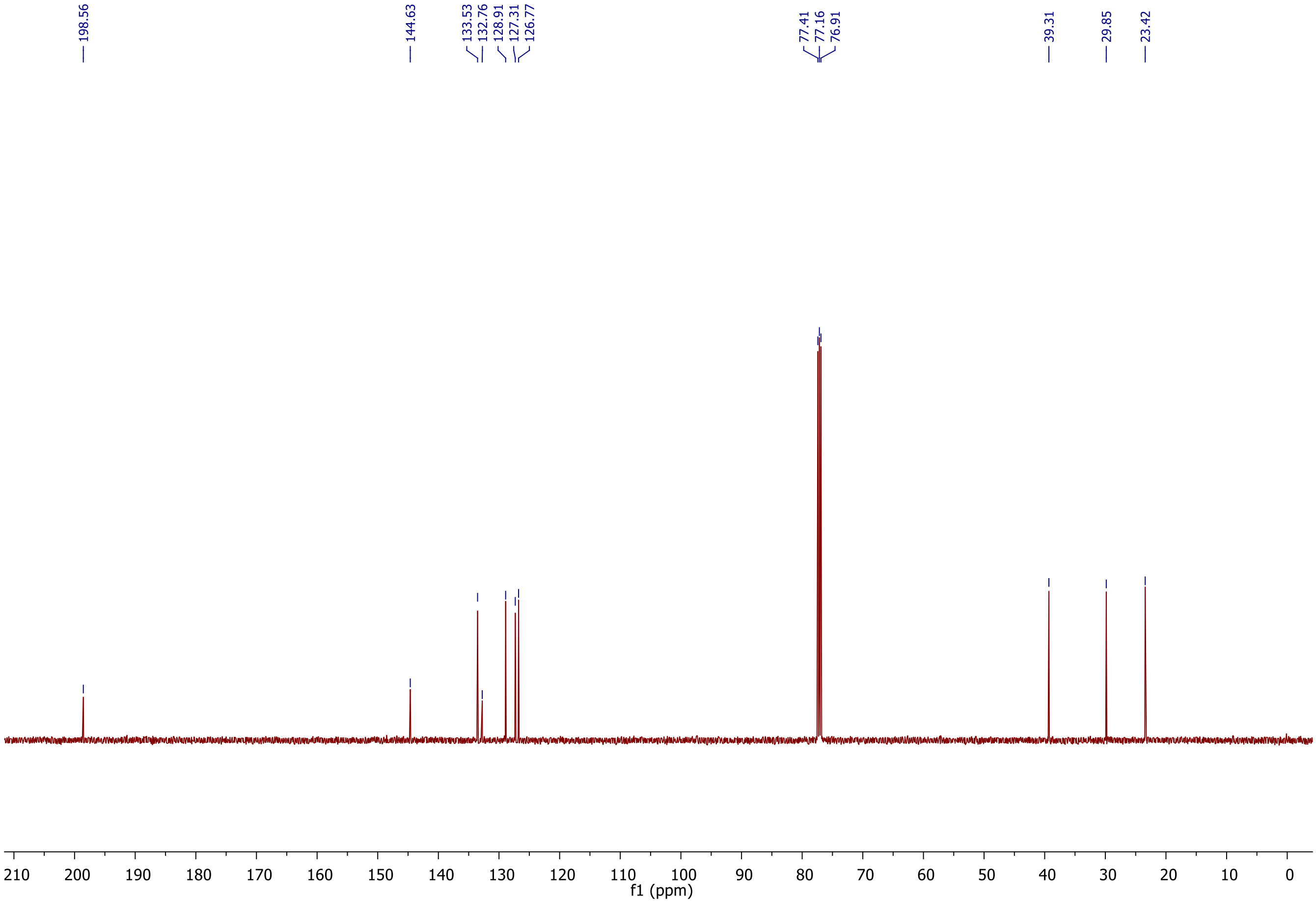
198.56
144.63
133.53 132.76 128.91 127.31 126.77
77.41 77.16 76.91
39.31
29.85
23.42
210 200 190 180 170 160 150 140 130 120 110 100 90 80 70 60 50 40 30 20 10 0
f1 (ppm)

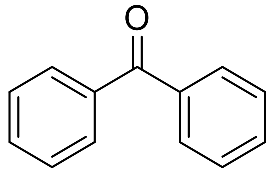
O

2ac

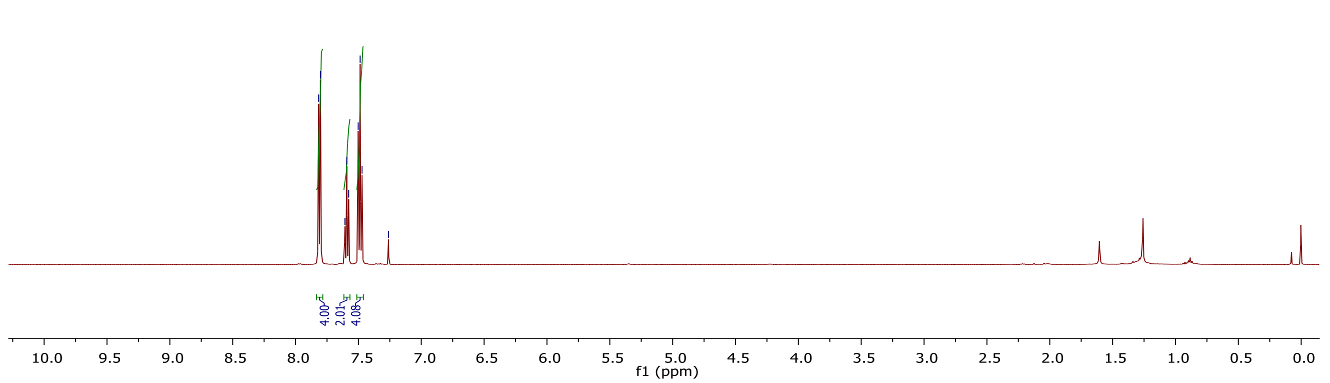
7.82
7.80
7.61
7.59
7.58
7.50
7.49
7.47
7.26
4.00
2.01
4.08
10.0 9.5 9.0 8.5 8.0 7.5 7.0 6.5 6.0 5.5 5.0 4.5 4.0 3.5 3.0 2.5 2.0 1.5 1.0 0.5 0.0
f1 (ppm)

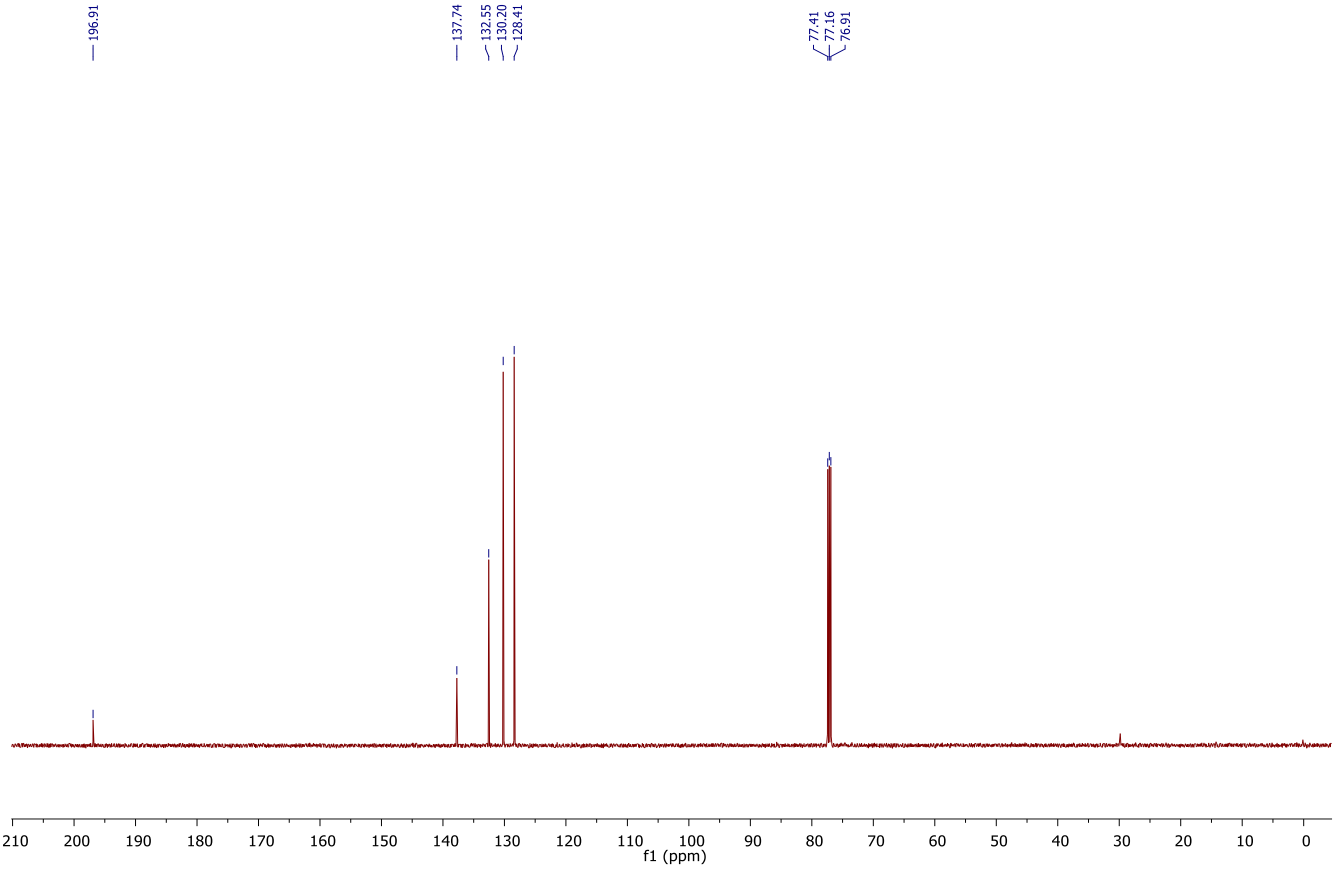
196.91
137.74
132.55
130.20
128.41
77.41
77.16
76.91
210 200 190 180 170 160 150 140 130 120 110 100 90 80 70 60 50 40 30 20 10 0
f1 (ppm)

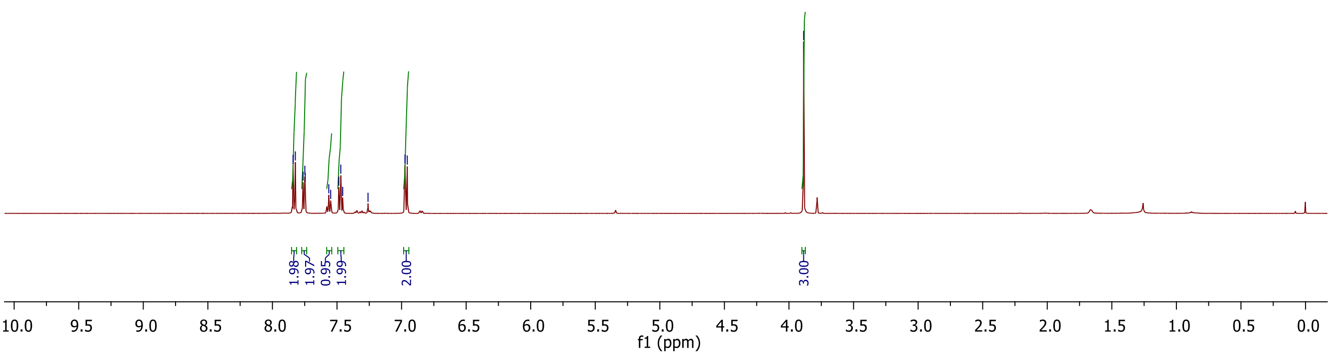

7.84
7.82
7.76
7.75
7.75
7.56
7.55
7.49
7.47
7.46
7.26
6.97
6.96
3.89
1.98
1.97
0.95
1.99
2.00
3.00
10.0 9.5 9.0 8.5 8.0 7.5 7.0 6.5 6.0 5.5 5.0 4.5 4.0 3.5 3.0 2.5 2.0 1.5 1.0 0.5 0.0
f1 (ppm)

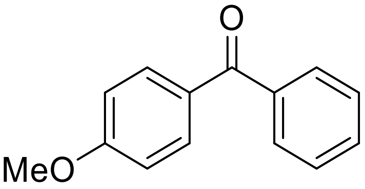

O
MeO

2ad

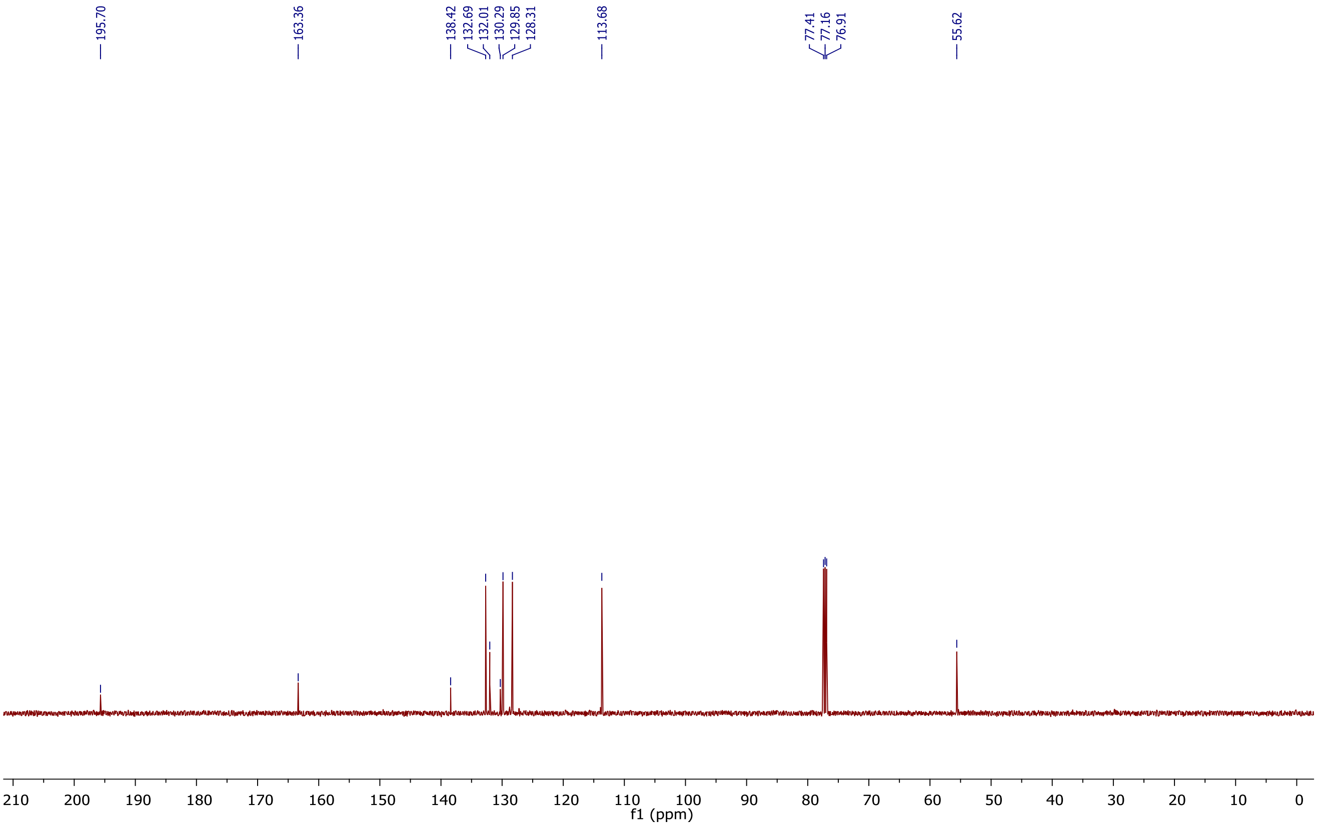

195.70
163.36
138.42
132.69
132.01
130.29
129.85
128.31
113.68
77.41
77.16
76.91
55.62
210 200 190 180 170 160 150 140 130 120 110 100 90 80 70 60 50 40 30 20 10 0
f1 (ppm)

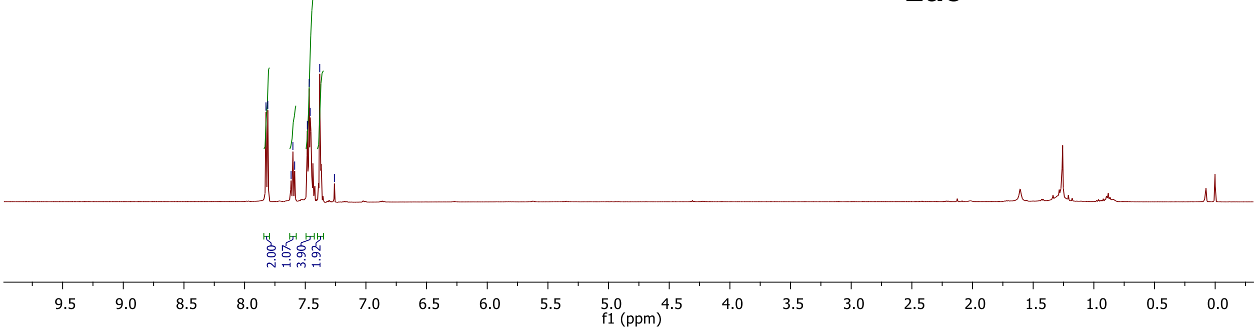
7.82
7.81
7.62
7.60
7.59
7.48
7.47
7.46
7.38
7.26
2.00
1.07
3.90
1.92
9.5 9.0 8.5 8.0 7.5 7.0 6.5 6.0 5.5 5.0 4.5 4.0 3.5 3.0 2.5 2.0 1.5 1.0 0.5 0.0
f1 (ppm)

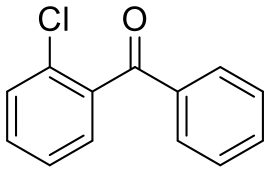
Cl
O

2ae

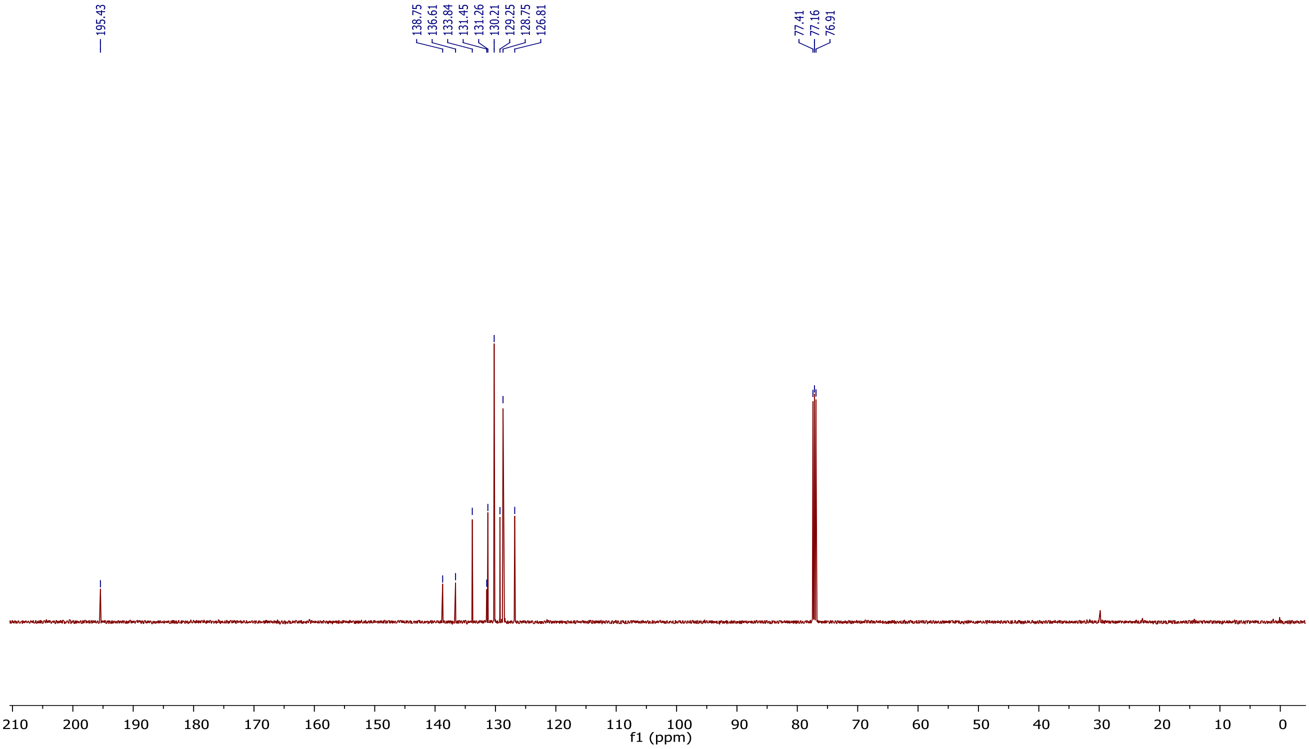
195.43
138.75
136.61
133.84
131.45
131.26
130.21
129.25
128.75
126.81
77.41
77.16
76.91
210 200 190 180 170 160 150 140 130 120 110 100 90 80 70 60 50 40 30 20 10 0
f1 (ppm)
